# Individualistic weight perception from motion on a slope

**DOI:** 10.1038/srep25432

**Published:** 2016-05-13

**Authors:** K. Zintus-art, D. Shin, H. Kambara, N. Yoshimura, Y. Koike

**Affiliations:** 1 Precision and Intelligence Laboratory, Tokyo Institute of Technology, Yokohama, Japan

## Abstract

Perception of an object’s weight is linked to its form and motion. Studies have shown the relationship between weight perception and motion in horizontal and vertical environments to be universally identical across subjects during passive observation. Here we show a contradicting finding in that not all humans share the same motion-weight pairing. A virtual environment where participants control the steepness of a slope was used to investigate the relationship between sliding motion and weight perception. Our findings showed that distinct, albeit subjective, motion-weight relationships in perception could be identified for slope environments. These individualistic perceptions were found when changes in environmental parameters governing motion were introduced, specifically inclination and surface texture. Differences in environmental parameters, combined with individual factors such as experience, affected participants’ weight perception. This phenomenon may offer evidence of the central nervous system’s ability to choose and combine internal models based on information from the sensory system. The results also point toward the possibility of controlling human perception by presenting strong sensory cues to manipulate the mechanisms managing internal models.

The ability to perceive and discern weight is active from birth^1^ and continues to develop with age^2^. A robust weight perception usually requires the combination of somatosensory and visual information. For example, one of the most common ways we interact with an object is by visually locating and then lifting up the object. However, throughout our daily lives, there are situations where somatosensory information is not available, such as when avoiding a falling or incoming object. Since we can only rely on visual information in such circumstances, the effect of visual information on weight perception warrants investigation.

A certain level of weight perception is possible through visual information, though it does not always reflect the true physical weight of the object^3^. This holds true even when only partial^4^,^5^ or no somatosensory information^6^ is available. This led to the conclusion that perception of weight is not a direct result of muscle activation^7^ and that visual cues certainly play a role in weight perception^8^ and estimation^3^. The influence of visual information on weight perception is also evident from a young age; children’s expectations of an object’s weight can be manipulated by altering the shape of the object^9^.

Visual information influences weight perception through two major elements: form and motion. Visually larger objects are usually viewed as heavier^10^,^11^,^12^,^13^. A classic example is the size-weight illusion^10^,^11^,^14^, which refers to the phenomenon when the larger of two objects of equal mass is perceived as weighing less than its smaller counterpart. Additionally, shape^15^, material^16^, and color^17^ have also been found to affect weight perception.

Motion, on the other hand, is the integration of displacement, direction, velocity, acceleration, and time. Therefore, the effects of motion on weight perception should be categorized accordingly. For displacement-related effects, larger objects are expected to exhibit greater downward displacement than smaller objects^18^. Note that the word “displacement” here refers to the participants’ expectation of the shift in location, not the actual displacement of an object. Displacement of objects in collision events can also be used to infer relative mass^19^. For velocity, upward motion of larger objects is perceived as slower than that of smaller objects^20^. For acceleration, decelerating or accelerating video sequences of an object being lifted result in perception of the object as heavier or lighter, respectively^21^.

Whether a motion is natural or not is also a topic of interest for weight perception studies. Newborns are found to have developed their own expectation of natural direction of acceleration at as early as 7 months of age^22^. Additionally, the unnatural difference in timing of load force exertion and when a falling ball hits a hand in a virtual environment can make the ball be perceived as heavier or lighter^23^.

The behavior of a motion is dictated by physics. When observing an object in motion, the factors that affect its qualities are not comprised solely of the physical properties of the moving object but also other factors such as the direction of motion and external actors that interact with the object. This includes the properties of the surrounding environment, such as inclination, surface texture and its friction, and the forces that exist within that environment that cannot be ascertained through vision alone (wind, pressure, air resistance, etc.). The complexity of having to account for all these factors is so substantial that we are often prone to making mistakes when making judgments from visual information^24^,^25^.

Humans tend to share the same opinion on what motion is considered natural. When subjects are asked to identify which motion displays a constant velocity, they choose the motion that closely resembles natural motion: “the motion that begins with a certain amount of acceleration, which later levels off to a constant velocity^26^”. Also, when a group of subjects observed a set of objects sliding down a slope, the preferred natural motion chosen by all observers was the one which briefly accelerated from start and then progressed with constant velocity^27^. Moreover, they made the assumption that faster moving objects are greater in size than slower moving objects. Using mathematical simulations and real-world experiments, we sought to verify whether or not that assumption was consistent with how an object actually slides down a slope (Fig. 1; for detailed calculations and recording of the experiment, see Supplementary Data). Results showed that the natural motion chosen by the observers was partly correct. Objects indeed undergo acceleration and later continue with near-constant or terminal velocity with air resistance in the Earth’s atmosphere. Slope also affects velocity, and friction from the material affects maximum terminal velocity. However, given the same mass and material, larger objects will exhibit lower terminal velocity than smaller objects due to the increased air resistance from the larger cross sectional area.

When comparing objects where the dimensionality of difference is small and does not involve invisible forces (e.g., when size or weight is the differentiating factor), the perception of motion in vertical and horizontal environments has been shown to yield consistent results^18^,^19^,^21^. However, in complex environments such as those with slope, where the effect of gravity, friction, and air resistance are more evident, is it possible to identify distinct motion-weight relationships? Would being exposed to complex environments disable or degrade perception? If such relationships can be identified, what environmental parameters and what magnitudes of difference in those parameters affect such relationships? Finally, if humans share a common concept of natural motion, would it also follow that we share a common perception of weight deduced from motion? To answer these questions, we used a tablet device to display a virtual environment simulating the sliding motion of two objects identical in shape and size (Fig. 2). Participants observed the objects’ motion in different environments and judged the relative heaviness of the two objects without haptic information. We sought to confirm whether a distinct relationship exists between perceived heaviness and motion towards Earth in complex environments. We also attempted to verify if humans share the same weight perception when observing objects sliding down a slope. Finally, we observed how perception of weight is affected by changes in environmental parameters.

## Results

**Figure 1:**
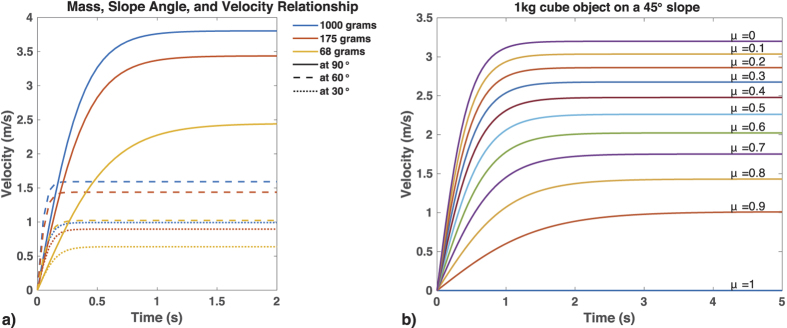
Relationship between mass, slope, and velocity. With air resistance, acceleration approaches zero as time passes. Given adequate time, objects in motion reach terminal velocity, depicted by the flat lines. (**a**) Objects with different mass reach their terminal velocity over time, given different slope angles. Line colors denote mass, and line types denote slope angle. The plot clearly shows that greater mass allows for greater terminal velocity. In this simulation, air density = 1.29 kg/m^3^, cross sectional area of the object = 1 m^3^, friction = 0.1, and drag coefficient = 1.05. (**b**) How different friction forces affect a 1-kg object placed on a 45° inclined slope. Apart from mass of an object, friction also affects terminal velocity. The plot shows a negative correlation between friction and velocity, where larger friction results in decreased terminal velocity, and vice versa.

**Figure 3:**
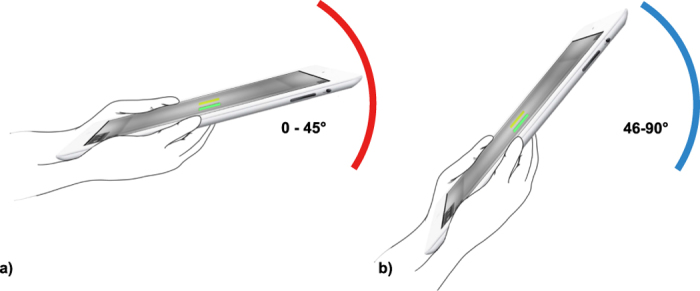
Virtual environments used in this study. We simulated a box containing two virtual objects: one yellow cube and one red cube. Participants tilted the environment at different angles and observe the objects’ motion in order to determine their relative weights. Three different environments were designed to study participants’ weight perception: (**a**) tilt angle restricted to less than or equal to 45°, (**b**) tilt angle restricted to greater than 45° but not exceeding 90°.

Forty-seven participants performed judgment tasks to select the heavier of two cubes presented in virtual environments (Fig. 3). Participants observed motion of the objects by tilting a tablet device under two different environments: tilt angle restricted to 45° or less (Environment 1, Fig. 1a) and tilt angle restricted to greater than 45° but not exceeding 90° (Environment 2, Fig. 1b). To control for order effects, the presentation order of the environments was counterbalanced, with twenty-four subjects presented Environment 1 followed by 2, and the remaining twenty-three subjects presented the opposite order. Participants performed 90 trials for each environment and, after every trial, made a judgment on which of the two cubes was heavier. They were then given a set of questions to confirm their selections and overall experience. Answers given by some of the participants were inconsistent with their selections, likely due to their having forgotten their choices. Since participants may have relied on inference when answering our questions, we excluded the questionnaire from our analysis.

**Figure 4:**
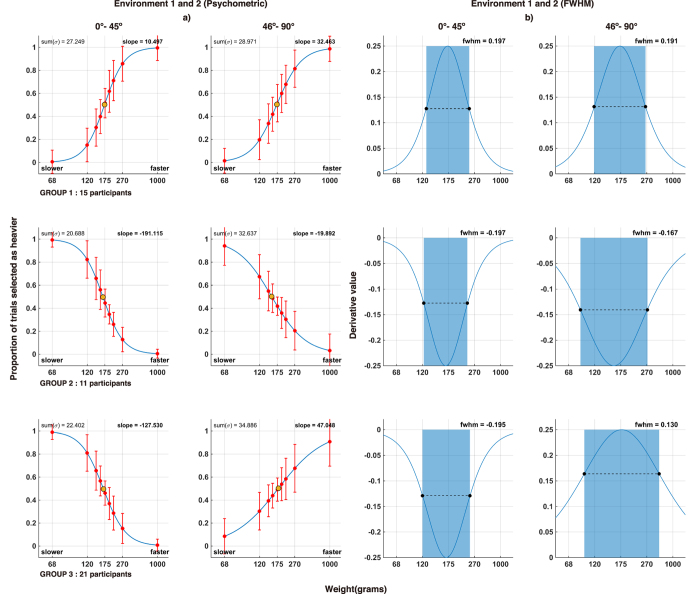
Group-average measurements showing (**a**) psychometric functions and (**b**) derivatives of the psychometric functions. The abscissas represent different weight values used in the experiment. The ordinates in (**a**) denote the proportion of trials (normalized percentage) in which the group perceived the comparison stimulus as heavier than the base stimulus. Red circles represent the proportion of trials for each weight value where participants perceive the comparison stimulus as heavier than the base stimulus. Orange circles represent the average just noticeable difference, where the participants started to notice the difference in motion. Error bars represent standard deviation for each weight value. The ordinates in (**b**) denote derivative values. The position of the peak indicates just noticeable difference similar to (**a**). The dashed lines and blue highlights depict full width at half maximum (FWHM). Note that the FWHM corresponds directly to the weight selection performance. The higher the performance in distinguishing the weight from motion, the smaller the FWHM.

We assumed two possible relationships for perceived heaviness and motion: 1) the heavier object should be faster or 2) the heavier object should be slower. We designed two representative psychometric functions (Supplementary Fig. 1) corresponding to the two relationships as the base models to determine which relationship type a behavior belongs to. Next, we modeled psychometric functions for all participants and for all conditions performed. We then computed the slopes at just noticeable difference (JND) and full width at half maximum (FWHM, Equation 6) for all psychometric functions and their derivatives, respectively. We compared each of the two FHWMs with that of the base models using Euclidean distance and were able to separate the participants into three different perception groups (Fig. 4), with ANOVA showing significant differences in each environment (F(2,44) = 315.4089, p < 0.0001 for Environment 1 and F(2,44) = 79.7627, p < 0.0001 for Environment 2). We then conducted a post hoc analysis using Tukey’s test to compare between the groups.

**Figure 5:**
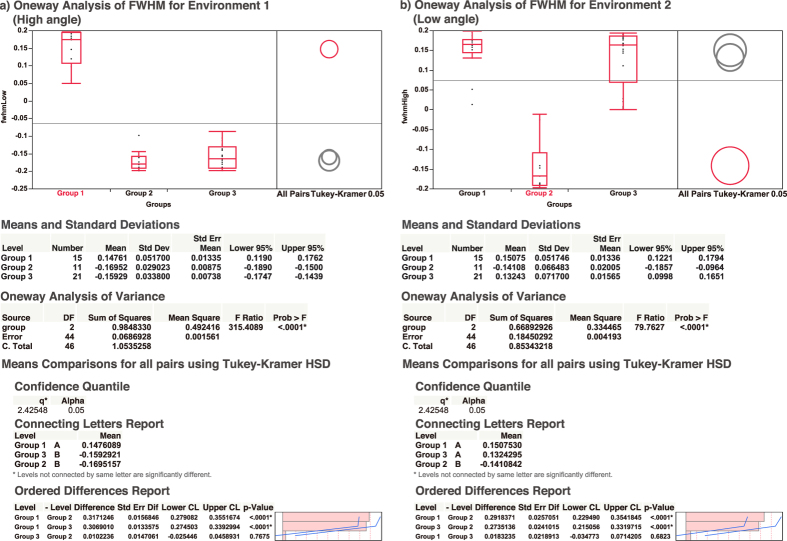
Detailed comparisons of the three participant groups. (**a**) Low tilt angle experiment (Environment 1). (**b**) High tilt angle experiment (Environment 2). The air resistance group is indicated by positive FWHMs for both environments. The friction group is indicated by negative FWHMs for both environments. The inverse group, however, has a negative FWHM for Environment 1 and a positive FWHM for Environment 2. The three groups are significantly different for both environments (F(2,44) = 315.408, p < 0.0001 for Environment 1, F(2,44) = 79.7627, p < 0.0001) for Environment 2). Group 1 and Group 2 are significantly different (p < 0.0001 for Environment 1, and p < 0.0001 for Environment 2). Group 3 is similar to the Group 2 in Environment 1 (p = 0.768) but significantly different from Group 1 (p < 0.0001). For Environment 2, however, Group 3 shows an opposite trend by being similar to Group 1 (p = 0.682) but different from Group 2 (p < 0.0001).

**Figure 6:**
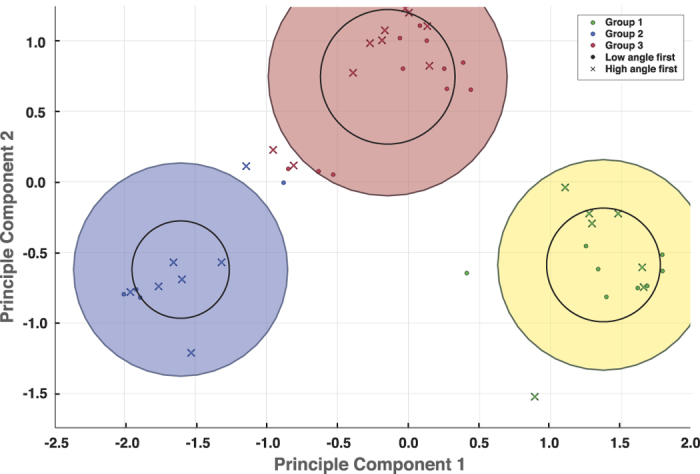
Biplot showing the result of K-means clustering analysis with 90% confidence interval. Each color represents each group of participants, where Group 1 judges faster moving objects as heavier, Group 2 judges slower moving objects as heavier, and Group 3 judges slower moving objects as heavier in Environment 1 but faster moving objects as heavier in Environment 2. No participants showed behavior opposite to Group 3. Comparison with our model matching classification method showed no misclassifications.

Group 1 showed strong bias in their perception, judging faster moving objects as heavier in Environment 1, where the virtual environment was restricted to a low angle range. Switching to higher tilting angle in Environment 2 did not overturn their judgment. Group 2 also showed strong perception bias, but in the exact opposite of Group 1 (p < 0.0001 for Environment 1, p < 0.0001 for Environment 2); they instead perceived faster moving objects as lighter in all environments. Group 3, on the other hand, exhibited a different perception trait from both Group 1 and Group 2. Group 3 perceived slower moving objects as heavier in Environment 1 (p = 0.768), similar to Group 2. However, Group 3’s perception was overturned in Environment 2 such that they perceived faster moving objects as heavier (p = 0.682), similar to Group 1. This is reflected by the change in slope and FWHM from negative to positive for Group 3’s psychometric functions (Fig. 4). No participants perceived faster moving objects as heavier in Environment 1 but then perceived slower objects as heavier in Environment 2. Figure 5 shows detailed comparisons of participant groups where both ANOVA and Tukey’s test confirms that the three participant groups are significantly different. We also used K-means to confirm if clustering without predefined labels would yield similar results to our model matching method. For the clustering features, we used two principal component vectors computed by running the FWHMs from both environments through principal component analysis. The clustering given by K-means showed identical results to our model matching method, with no misclassifications between the two (Fig. 6).

Looking at the psychometric functions of the three subject groups in terms of individual environment, we determined that 36 out of 47 participants perceived faster moving objects as heavier than slower moving objects for motion at high tilt angles. Additionally, 32 participants perceived slow moving objects as heavier for motion at low tilt angles. Individual and trial classification results can be found in the Supplementary Data.

## Discussion

The first question we sought to answer in this study was whether we can perceive weight from sliding motion on a slope. Based on existing findings on the relationship between form, motion, and weight perception^10^,^11^,^12^, as well as the understanding that downward displacement is a subjective experience which draws upon a lifetime spent in Earth’s gravitational field^28^ and assumes air resistance effects on motion^26^,^27^, it is within reason for the participants to assume that motion towards Earth with greater velocity, acceleration, or other related parameters would imply more weight. Therefore, we expected that our participants’ answers would reflect a well-established relationship between slope motion and weight. Our results (Fig. 4) confirmed our expectation and answered the first question. The modeled psychometric functions portray an established relationship where the participants see motion as a critical deciding factor of an object’s weight. As the comparative difference in motion increases, the participants’ tendency to perceive an object as heavier also increases.

**Figure 7:**
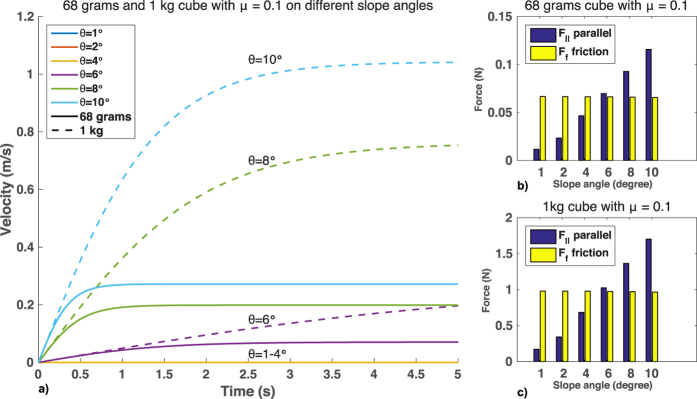
Relationship between slope angle, friction force, and velocity. (**a**) Velocity against time for a 1-kg and a 68-g cube with friction coefficient of 0.1 is plotted for 1° to 10° slope angles. No sliding occurred (velocity = 0 m/s at all times) at 1°, 2°, and 4° slopes. (**b**) Comparison of F_p_ and F_f_ for a 68-g cube with 0.1 friction coefficient. As slope angle declined, the cube required more force to overcome friction and start moving. (**c**) Comparison of F_p_ and F_f_ for a 1-kg cube with 0.1 friction coefficient. The angle where F_p_ overcomes F_f_ is the same for both the 68-g cube and the 1-kg cube.

Surprisingly, we also found that some participants’ perceptions of motion were not always consistent across environments. This finding is in contrast to previous works where the same direction of motion resulted in similar perception^18^,^20^,^21^,^22^. In our results, while Group 1 and Group 2’s perceptions remained relatively consistent across both the low and high tilt angle environments, Group 3’s perception did not. Physics dictates that if two objects of identical shape and size are on a downward motion due to gravity, with the presence of air resistance, the object with more mass will have a higher terminal velocity. However, in primary physics, sliding down a slope is explained using free fall mechanics that assume no drag or resistance force. Sliding will not occur if the force parallel to the slope (F_p_) is smaller than the friction force (F_f_), and thus a force additional to gravity is required to move the object. This situation is usually linked to either a leveled plane or high friction coefficient. Our simulations showed that this can also occur for an object with a friction coefficient as low as 0.1 given a very gradual slope (Fig. 7). Participants may have interpreted faster moving objects as receiving greater influence from the tilt angle. This provided us with a possible clue as to why the low tilt angle in Environment 1 made Group 3 perceive faster moving objects as lighter.

It could be argued that participants may have made weight judgments based on inferences regarding heaviness rather than perception of weight from motion. Inference occurs when an observer judges an event based on what he or she thinks the outcome ought to be rather than what is perceived^26^. We believe our protocol adequately ruled out inference, as we instructed the participants to choose the color of the cube whose movement *looked* heavy. The experiment was also designed to have participants make quick decisions and not overthink them, with selection user interface elements presented on the screen and the next trial starting immediately after each selection (Supplementary Fig. 2). This encouraged participants to make their selections based on perceptions of motion rather than inferences.

The second question we sought to answer was how changes in the parameters of an environment affect weight perception from motion. As stated earlier, for an object to slide under gravitational force, force parallel to the slope (F_p_) must be greater than friction force (F_f_). Higher slope angle brings F_f_ closer to zero, minimizing its effect. Therefore, Group 3 may have interpreted Environment 2 as similar to free fall, where air resistance has more influence. These findings supported our expectation that using different environmental parameters can affect perception and answered our second question: changes in slope inclination can affect how individuals perceive weight from motion.

Applying the aforementioned explanations to Group 1 and Group 2, we can infer that Group 1 perceived the weight under the presumption of air resistance, while Group 2 perceived the weight under the presumption of friction. Consistent perception in Group 1 and Group 2 even after the task order was counterbalanced further supports this interpretation.

Finding the three participant groups to have different presumptions about motion, what computational mechanism in the brain could be responsible for this difference? One possibility is the internal models^29^,^30^ for the neural mechanisms and computational principles behind predictive and learning abilities^31^,^32^. These models are acquired through experiences with objects and environments such as new tools^33^,^34^,^35^ and gravity^36^,^37^. Therefore, participants may have used their experiences to predict the object’s weight through the combination of the environments and their own notions of how mass should affect motion in those environments.

Our daily life experiences teach us the dynamics of various environments and how objects behave in them. Numerous phenomena involving either horizontal or vertical motion help us form respective internal models. These internal models form our awareness of what pairing of weight and motion is deemed *natural*. This explains why we have common weight perception for moving objects in vertical and horizontal environments: we judge faster falling objects in water as heavier and objects gliding faster across a leveled surface as lighter. However, our exposure to phenomena for tilted surfaces is typically less and varies from person to person. This results in different or underdeveloped internal models for such environments. An internal model study using ball catching under microgravity^38^ showed a very relevant result. Their participants anticipated the effect of gravity due to their familiarity with Earth’s gravity and started to reach out for the falling ball too early. The involvement of experiences in the formation and modification of internal models has also been demonstrated. A study on the effect of temporal perception on weight perception showed that gradual training can help with acquisition of internal models for modified load contact timing^23^. Furthermore, exposure to the reverse relationship of size and weight for an extended period of time can alter internal models and thus reverse perception of the size-weight illusion^39^.

**Figure 8:**
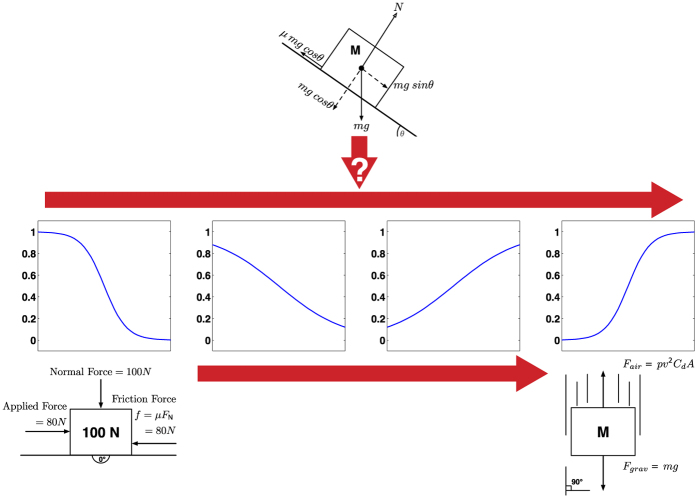
Model of the participants’ behavior when perceiving weight within unfamiliar environments with complex physics. The extreme ends represent the basis models containing physics encountered in real life, but in completely different environments: friction (0°) and air resistance (90°). The psychometric functions exhibit sinusoidal shapes corresponding to the presumption or weight assigned to each extreme. The weight decision for unfamiliar environments can be seen as the combination of inputs from the two extremes. The influence of each internal model used depended on the environment, expectations, and individual experiences^48^,^49^.

In a similar manner, participants combined their existing internal models to assess object motion in unfamiliar environments^40^ to cope with the lack of a reference model. The respective influence of each internal model used depended on the state of the environment the participants interacted with, their expectations of the object’s motion, and their individual experiences with slope sliding motion (Fig. 8).

In conclusion, our findings showed that humans can perceive weight from slope sliding motion without somatosensory information. The results suggest that weight perception can be subjective and that differences in environmental parameters, such as incline or surface texture, when combined with subjective experience, affect weight perception from motion. This highlights the possibility that weight perception can be controlled if these causal differences are properly configured. Understanding these perception behaviors could also benefit technological innovation and development in human-computer interaction.

## Methods

Forty-seven adults (25 males and 22 females; 25.85 ± 5.7 years of age) participated in the experiment. All participants were recruited from our institute and gave their informed consent before participating. Participants had normal or corrected to normal vision and reported no cognitive or motor disorders. No prior information about design or purpose was revealed to the participants before the experiment. The protocol and procedures used in this experiment were approved by the Ethical Review Board for Epidemiological Studies of the Tokyo Institute of Technology (approval number: 2012010). The methods were carried out in accordance with the approved guidelines.

To simulate sliding on a slope in a tablet device, we created a virtual environment consisting of a box with two cube objects inside. A 9.7-inch high-resolution display tablet with an up to 16.4 LSB/°/s gyro sensitivity built-in gyroscope was used in this experiment. Providing only visual information, this method inhibited the participants’ ability to differentiate between stimuli using haptic information. We reproduced the dynamics using the Unity3D physics engine (Unity Technologies, San Francisco, CA). We allowed the objects to move in response to the participants’ interactions with the tablet device by applying a Hamilton product of rotation quaternions to the gravity vector such that:


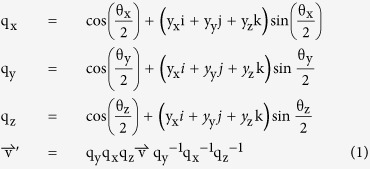


where q_x_, q_y_, and q_z_ are rotation quaternions for the three axes, x, y, and z are unit vectors corresponding to the three axes, θ_x_, θ_y_, and θ_z_ are the gyroscope readings corresponding to their respective axes in radians, 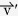 is the updated gravity vector, 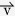 is the original gravity vector, and q_y_^−1^, q_x_^−1^, and q_z_^−1^ are the inverse of their quaternion counterparts. We also applied visual techniques, including off-axis projection^41^,^42^, to simulate glassless 3D effects and real-time lighting control to increase visual fidelity.

Two virtual cube objects with two different colors, yellow and green, were displayed at the upper part of the screen. The volume of each virtual box in the miniaturized virtual environment was 1 unit and started at 0 m/s initial velocity. Since mass cannot be specified in metric units in Unity3D, we looked to our mathematical simulation for the relationship between terminal velocity and mass (Supplementary Data). Greater mass contributes to higher velocity. Since we specified all physics values to be the same for both objects, excluding their masses, terminal velocity could be used to calculate object mass on a metric scale using:


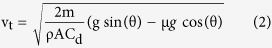


where v_t_ is the terminal velocity, m is the object mass, *ρ* is the air density, A is the reference area, C_d_ is the drag coefficient of the object, g is gravity, and *μ* is the friction coefficient. All variables except mass were shared between the two objects, allowing simplification to:


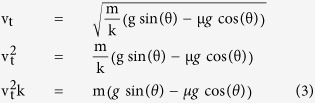


To study the participants’ perception when observing motion in different virtual environments, two experiment sessions with the following environments (Fig. 1) were presented to the participants:

- Environment 1: Tilt angle restricted to 45° or less.
- Environment 2: Tilt angle restricted to greater than 45° but not exceeding 90°.

Ninety trials were performed for each environment, with each trial consisting of a two-alternative forced choice on whether the yellow or green cube was heavier. Prior to the experiment, we had our participants read written instructions on how they should interact with the two virtual environments. We then described the task, environments, user interface elements, and how to make a selection on which cube was heavier. During the experiment, participants sat in a relaxed posture on a chair while holding the tablet device with both hands. To address possible order effect, we separated the participants into two groups consisting of 24 and 23 people, respectively. The two groups performed the two sessions, with the first group starting from Environment 1 and the second starting from Environment 2. Each environment took approximately 30 minutes. An automated tilt angle detection mechanism was implemented in the virtual environment. If the participants tilted the device to an angle larger than the specified range, an alert message would be displayed on the screen to notify the participants. Nine different weight values ranging from 68 to 1000 g were selected based on their differences in resultant terminal velocity, with 175 g chosen as the base stimulus. Previous research has shown that participants link object weight to color^17^. To determine if such influence existed in our data, we randomly assigned a weight value to the comparison stimulus in each trial. Furthermore, the base stimulus was also randomly switched between the two cubes. The participants performed tests to choose the object they perceived to be heavier. To ensure consistency of the decisions made by the participants, each weight value appeared 10 times. While the sliding of the objects was not constrained to only one axis (to maintain realism), we stressed that the participants should tilt their tablet only in the x-axis to retain uniformity between participants throughout the experiment. The selection data and the gyroscope readings from the start of each trial to selection were stored for subsequent analysis. The average time each participant spent in the experiment was approximately 1 h.

### Data analysis

All experiment data were analyzed using MATLAB and its Statistics Toolbox (Mathworks, Natick, MA) and JMP (SAS Institute Inc., Cary, NC). From the obtained judgment data, we checked the gyroscope recordings to ensure that the tilting done by the participants was in accordance with the written instructions for each environment. Then, psychometric functions for each participant were modeled using generalized linear model regression. The probability of judging that “the comparison stimulus is heavier than the base stimulus” was acquired by applying a logistic regression model^43^,^44^ to the participants’ judgments:


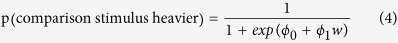


where w is the weight of the comparison stimulus and *ϕ*_0_ and *ϕ*_1_ are the regression coefficients. With the psychometric functions for all subjects and all experiments modeled, we categorized the participants according to their differences and similarities. We then created psychometric functions for the resultant groups. The point of subjective equality (PSE), where w gives p = 0.5, can be calculated as PSE = 
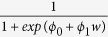
.

We then performed a slope analysis^45^ to find the slope at PSE by computing the first derivative of the psychometric function. The sum of standard deviations (*σ*) from each weight value for each group were also calculated to evaluate variance in the psychometric functions. If the participants had associated object weight to color and did not observe object motion in the virtual environments, the resulting psychometric functions would be random and not convey any weight-motion relationship. This was not the case in our experiment. However, we later realized that the slope value from the linear predictor was limitless, as we observed very high variance between the participants’ slopes. Since the psychometric function was essentially a sigmoid function, we therefore looked at its first derivative, which returns a normal distribution function with 0.25 as the highest value^46^:


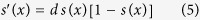


To find a measurement that indicates the overall performance of an experiment task, we calculated the full width at half maximum (FWHM) of the sigmoid derivative:


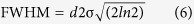


where *d* is slant direction of the psychometric function. A *d* value of 1 indicates that the psychometric function slants to the right, whereas −1 indicates the that psychometric function slants to the left. This resulted in a distribution with its peak pointing upward if the participant judged the faster moving object as heavier, and vice versa. A smaller width in the distribution would indicate higher performance and consistency in judging weight from motion.

We designed two psychometric functions to represent two motion-weight relationships: faster moving objects are heavier and slower moving objects are heavier. To determine which relationship type a participant belonged to, we compared the participant’s FHWMs from the two environments with that of the representing psychometric functions’ using Euclidean distance. We performed a one-way ANOVA to determine if the difference between groups for each environment was significant. We then compared the means for each pair of groups using a post hoc Tukey’s test. K-means clustering was applied for visualization and detection of any inconsistency with our grouping.

### Classification Model Construction with SVM

We constructed a classification model using LIBSVM^47^ to test on our judgment data. Dummy data simulating inversion from judgment of faster objects as heavier in the low angle environment to faster objects as lighter in the high angle environment were added because no participants exhibited such behavior. For the classification features, we use the principal component vectors computed by running the FWHMs from both environments through principal component analysis. Cost and gamma parameters were set to 50 and 1, respectively, using 3-fold cross validation. Classification results and the predicted boundaries are shown in Supplementary Fig. 3. Observing the distribution, we determined that the boundary for each group could be simplified, as the slant of the psychometric function can be decided from the sign at FWHM. Since K-means clustering provided satisfactory results, we decided to use that method for our primary analysis.

## Additional Information

**How to cite this article**: Zintus-art, K. *et al*. Individualistic weight perception from motion on a slope. *Sci. Rep.* **6**, 25432; doi: 10.1038/srep25432 (2016).

## Supplementary Material

Supplementary Information

Supplementary video

## Figures and Tables

**Figure 2:**
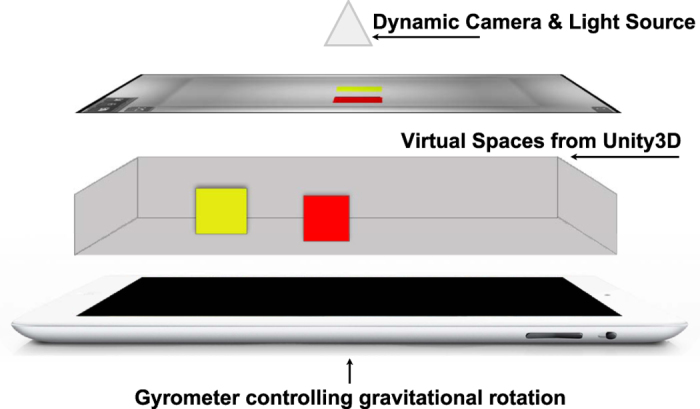
Virtual environment used in this study. Four primary components of the interface: camera, virtual space, stimulus, and gyro sensor. All the components act in coordination to create a convincing sliding motion simulation. To provide the participants with a means to interact with the virtual environments, we mapped the rotation of the virtual environment to the tablet’s gyro sensor, providing real-time rotational control of the virtual environment. We also implemented off-axis projection techniques to create a realistic projection of the box interior. The technique allows users to view the screen as if they are looking through a window and perceive more depth. The lighting was dynamically controlled to provide more visual fidelity.
