# Supplementary material for: Individualistic weight perception from motion on a slope
Source: Sci Rep. 2016 May 13;6:25432. doi: 10.1038/srep25432 (PMC4865871; doi:10.1038/srep25432)
Supplement: Supplementary Information [file srep25432-s1.pdf]

Individualistic weight perception from motion on a slope

K. Zintus-art, D. Shin, H. Kambara, N. Yoshimura, Y. Koike

koike@pi.titech.ac.jp, kalanyuz@cns.pi.titech.ac.jp

Precision and Intelligence Laboratory, Tokyo Institute of Technology, Yokohama, Japan

Supplementary Methods: Mathematical simulations of objects sliding on a slope.

Air-resistance is described as:

$$F_d = \frac{1}{2}\rho v^2 A C_d \tag{1}$$

where ρ is the density of the medium (air), v is the speed of the object relative to the medium, A is the cross-sectional area, and C_d is the drag coefficient.

Net force on an inclined plane with air-resistance is calculated as:

$$F_{net} = F_g - F_f - F_d = mg\sin(\theta) - \mu\, mg\cos(\theta) - \frac{1}{2}\rho v^2 A C_d \tag{2}$$

where F_g is the gravitational force, F_f is the friction force, and F_d is the drag force. The buoyant force is ignored because it is relatively smaller than other forces in the air. To simplify, we rewrite the above equation using Newton's second law with a coefficient, $k = \frac{1}{2}\rho A C_d$:

$$F_{net} = m\frac{dv}{dt} = mg\sin\theta - \mu mg\cos\theta - kv^2 \tag{3}$$

$$\frac{dv}{dt} = (g\sin\theta - \mu g\cos\theta) - \frac{k}{m}v^2 \tag{4}$$

Terminal Velocity $\frac{dv}{dt} = 0$ where friction and air-resistance exists:

$$v_t = \sqrt{\frac{m}{k}(g\sin\theta - \mu g\cos\theta)} \tag{5}$$

In order to integrate this system, we rearrange the equation (3) for modeling the *v(t)* function:

$$dv = \left(g\sin\theta - \mu g\cos\theta - \frac{k}{m}v^2\right)dt$$

$$dv = \frac{k}{m}(v_t^2 - v^2)dt \tag{6}$$

$$\frac{dv}{v_t^2 - v^2} = \frac{k}{m}dt$$

$$\int_0^{v(t)} \frac{dv}{v_t^2 - v^2} = \int_0^t \frac{k}{m}dt \tag{7}$$

$$\frac{\tanh^{-1}(\frac{v(t)}{v_t})}{v_t} = \frac{k}{m}t \quad (8)$$

$$v(t) = v_t \tanh(v_t \frac{k}{m} t) \quad (9)$$

In simulation, the values assigned to ρ, A, C_d are 1.29kg/m^3, 1m^3, and 1.05, respectively. For simulation results, see Figure 1 and Figure 6.

Supplementary Figure 1

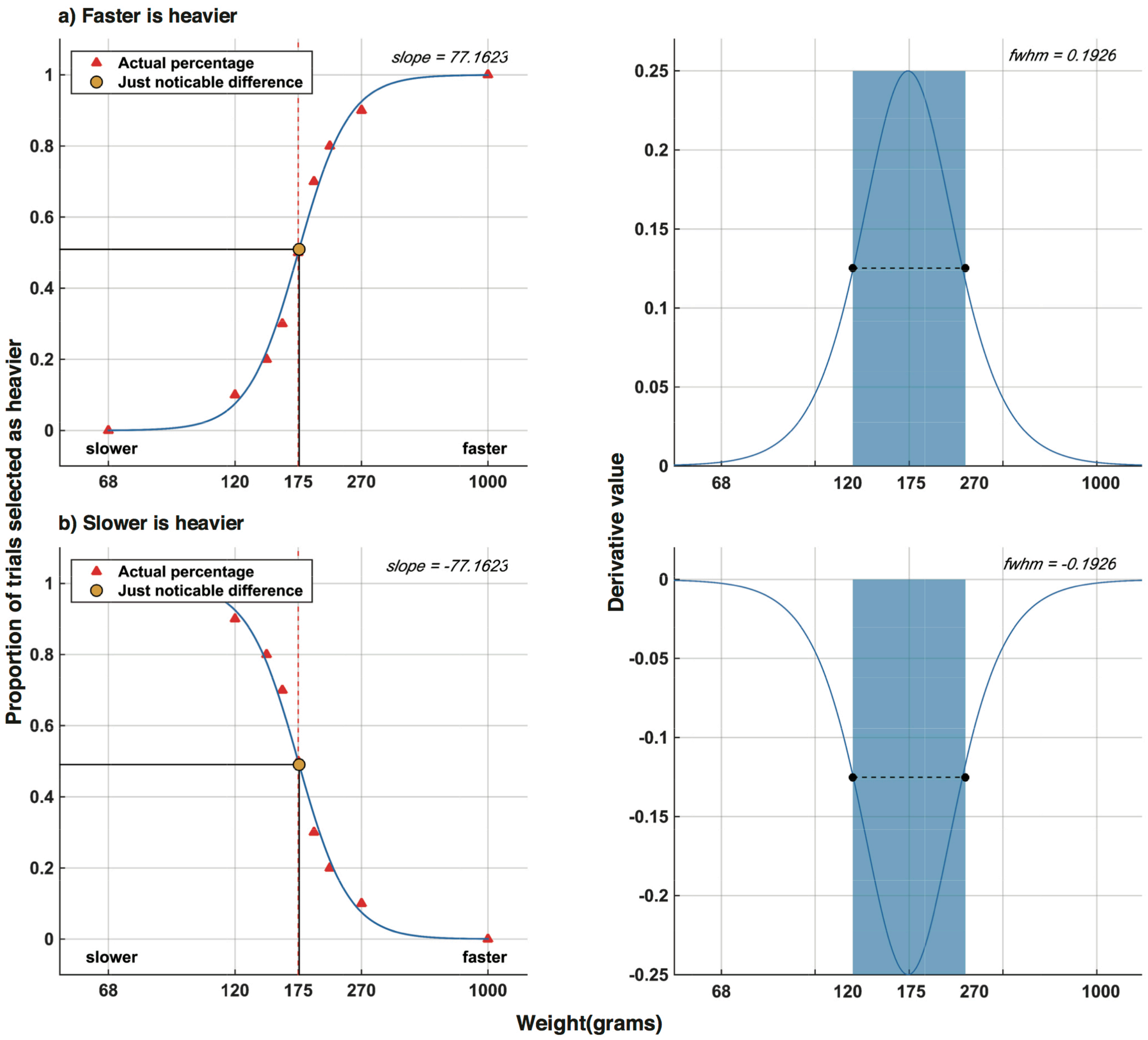

Supplementary Figure 2

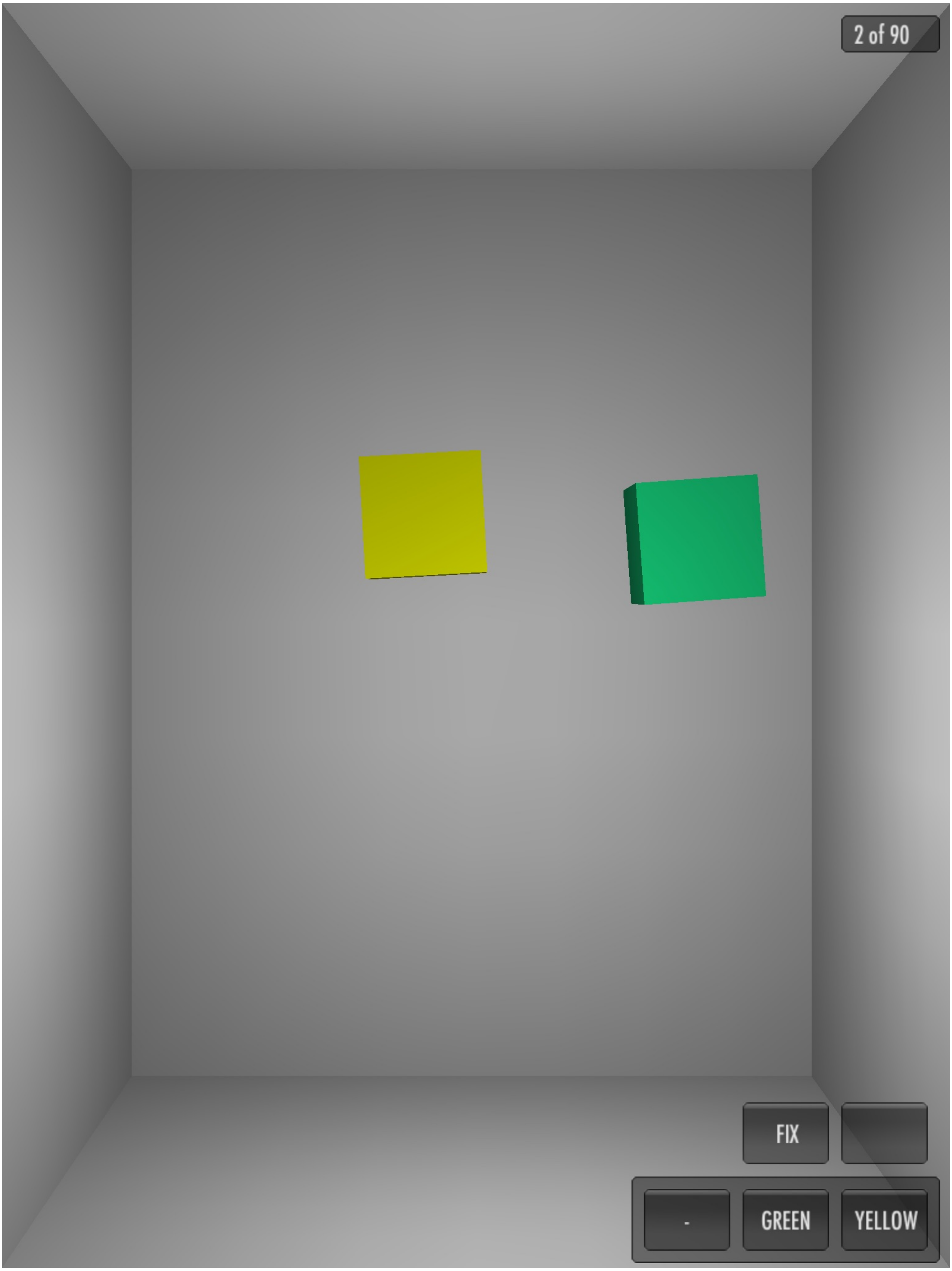

Supplementary Figure 3

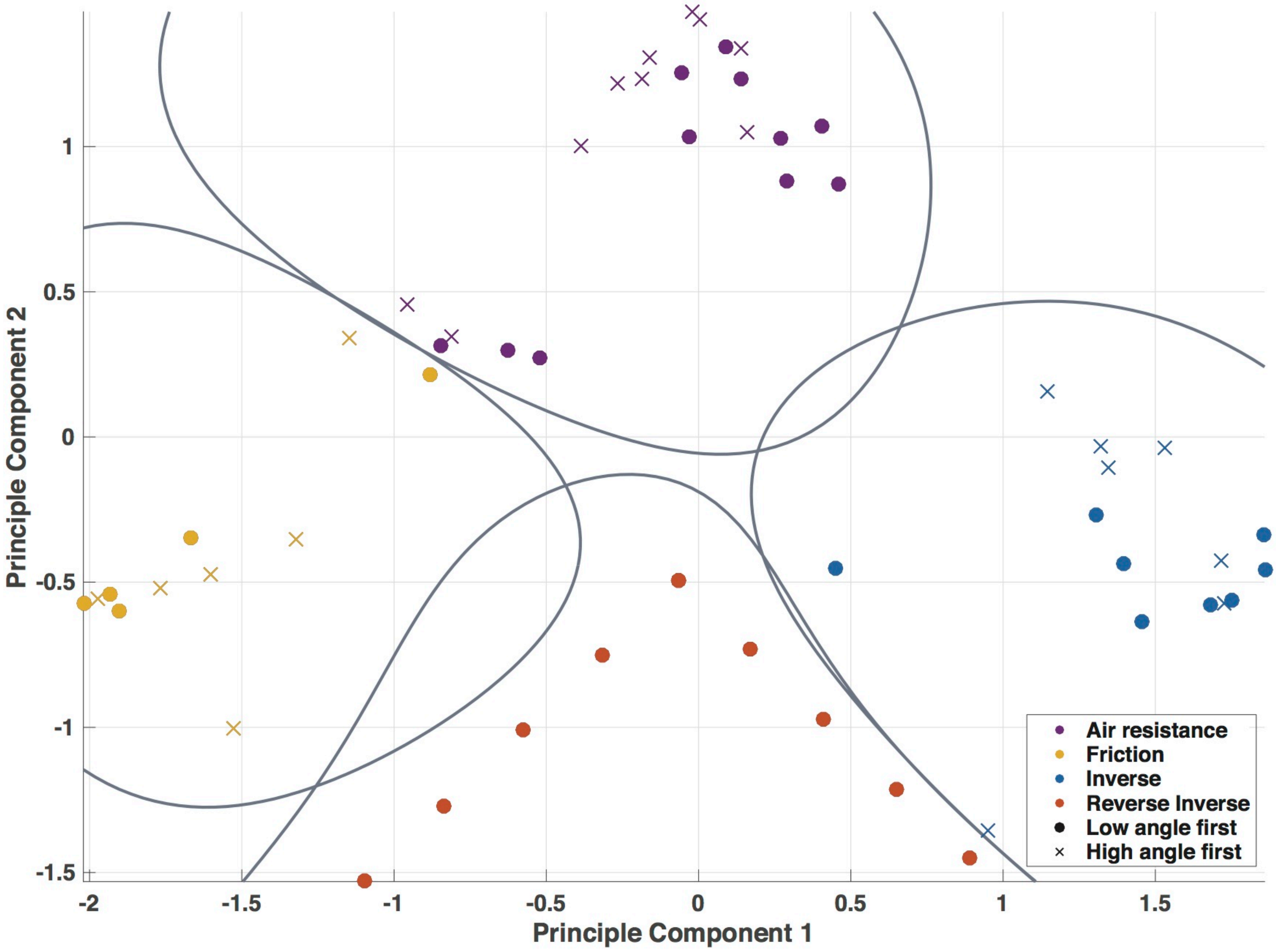

Supplementary Figure Legends

Supplementary Figure 1) Models for two different motion-weight relationships and their derivatives: (a) faster moving objects are heavier and (b) slower moving objects are heavier. The models are used to determine which group a participant belongs to. Other model variations (e.g., decreasing the ability to discern weight from motion to just above chance level or increasing it to 100% correct for all weight values) had no effect on the results.

Supplementary Figure 2) Virtual environments used in this study. Participants had to select whether the green cube or the yellow cube was heavier by observing their motion. The user interface allows easy access and fast responses. This in turn helps maintain focus and encourages selection based on perception rather than inference.

Supplementary Figure 3) Visualization of classification model with SVM (cost = 50, gamma = 1). *Reverse Inverse* represents simulated data that inverts from judging faster objects as the heavier in the low angle environment to judging faster objects as lighter in the high angle environment.

Supplementary Video 1) Recording of objects sliding on an angled plane. The video compares the sliding on an angled plane of two objects with the same material but different weight (995 g and 105 g, respectively). When the two objects were set in motion at the same time, the heavier object slid down the plane faster than the lighter object.

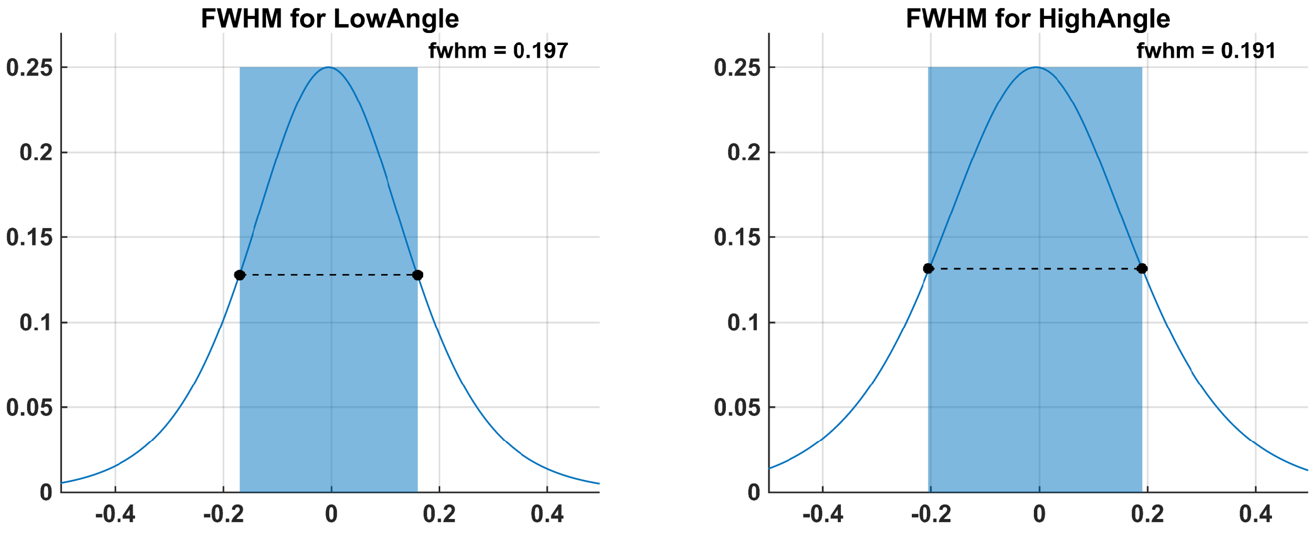

Individual participant data for air resistance group

Individual data for each participant from the air resistance group, namely the group which believes faster objects to be the heavier in both environments. This page is the group average. From page 8-22, each page corresponds to the data from each participant.

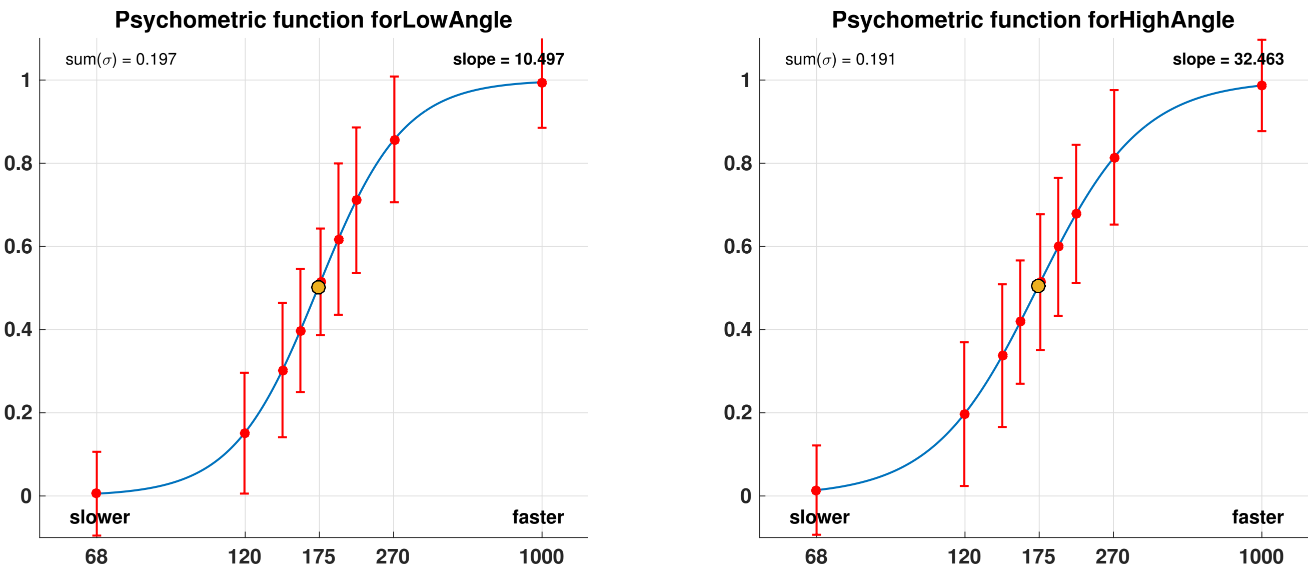

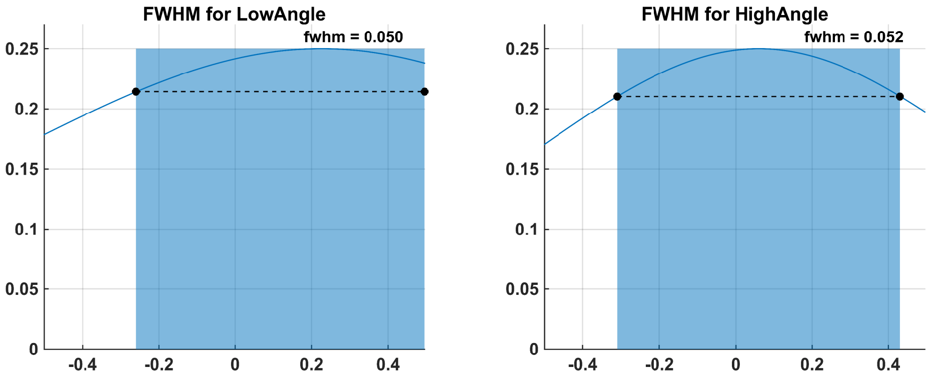
FWHM for LowAngle
fwhm = 0.050
FWHM for HighAngle
fwhm = 0.052
0.25
0.2
0.15
0.1
0.05
0
-0.4
-0.2
0
0.2
0.4

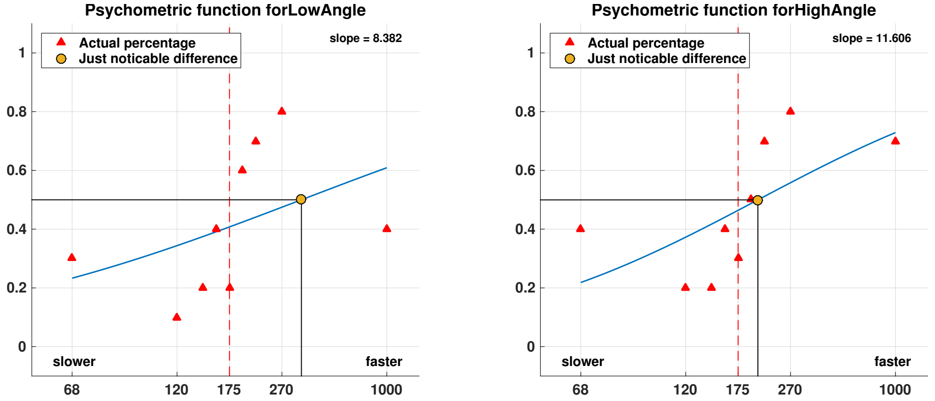
Psychometric function forLowAngle
Actual percentage
Just noticable difference
slope = 8.382
Psychometric function forHighAngle
slope = 11.606
slower
faster
68
120
175
270
1000

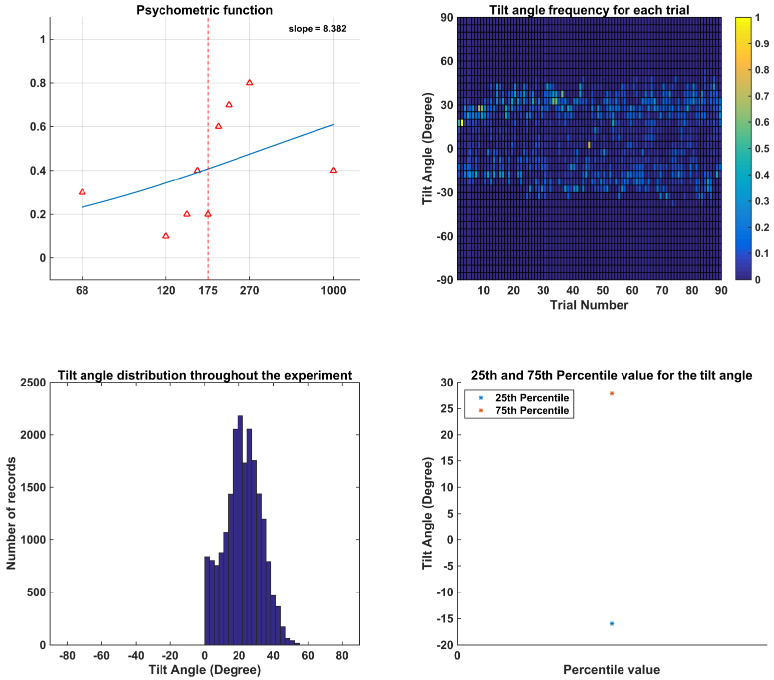
Psychometric function
slope = 8.382
Tilt angle frequency for each trial
Tilt Angle (Degree)
Trial Number
Tilt angle distribution throughout the experiment
Number of records
Tilt Angle (Degree)
25th and 75th Percentile value for the tilt angle
25th Percentile
75th Percentile
Percentile value

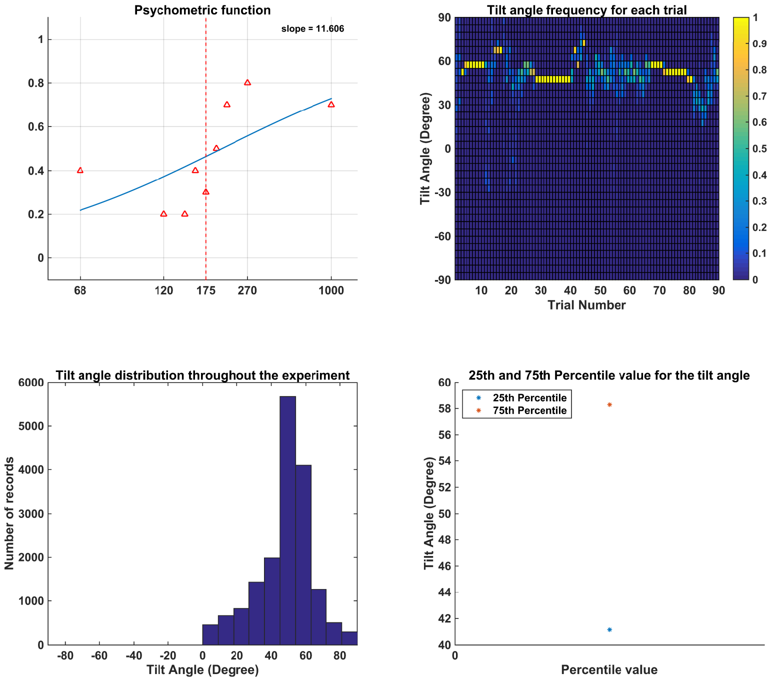
Psychometric function
slope = 11.606
Tilt angle frequency for each trial
Tilt Angle (Degree)
Trial Number
Tilt angle distribution throughout the experiment
Number of records
Tilt Angle (Degree)
25th and 75th Percentile value for the tilt angle
25th Percentile
75th Percentile
Percentile value

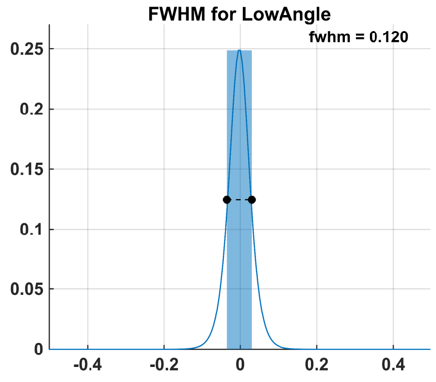
FWHM for LowAngle
fwhm = 0.120

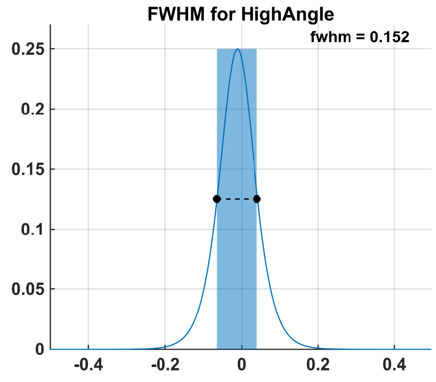
FWHM for HighAngle
fwhm = 0.152

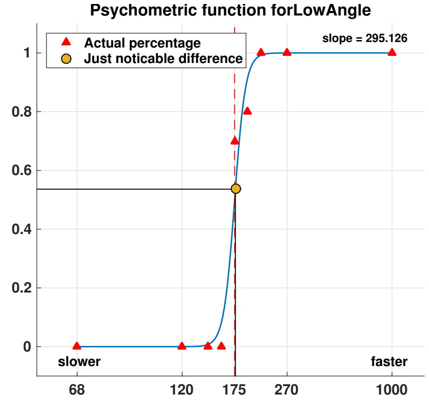
Psychometric function forLowAngle
Actual percentage
Just noticable difference
slope = 295.126
slower
faster

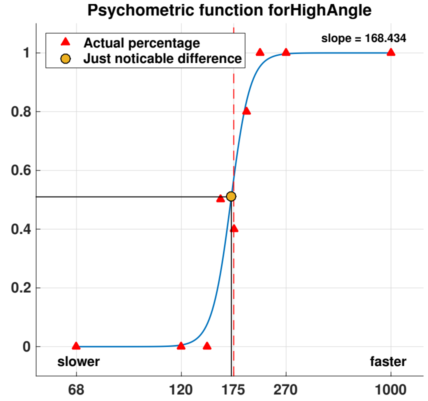
Psychometric function forHighAngle
Actual percentage
Just noticable difference
slope = 168.434
slower
faster

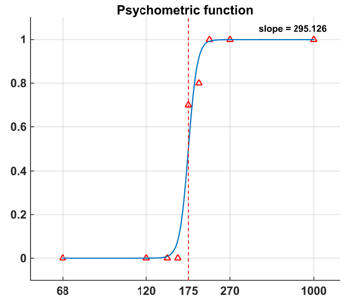
Psychometric function
slope = 295.126

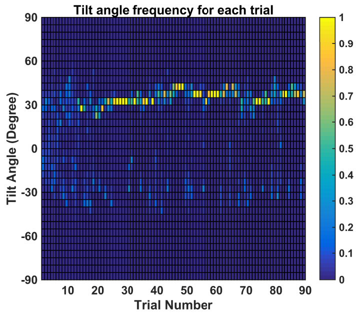
Tilt angle frequency for each trial
Tilt Angle (Degree)
Trial Number

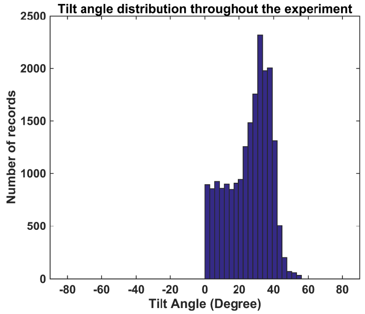
Tilt angle distribution throughout the experiment
Number of records
Tilt Angle (Degree)

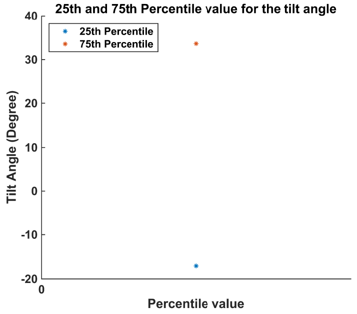
25th and 75th Percentile value for the tilt angle
25th Percentile
75th Percentile
Tilt Angle (Degree)
Percentile value

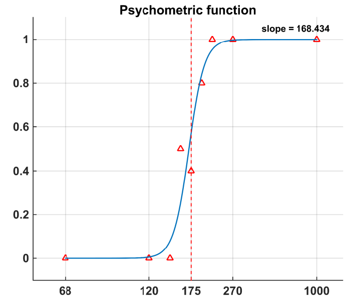
Psychometric function
slope = 168.434

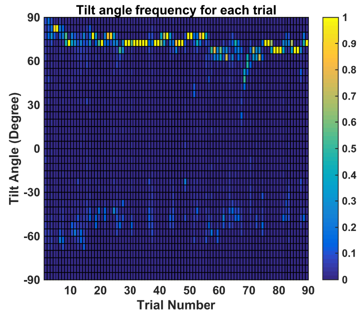
Tilt angle frequency for each trial
Tilt Angle (Degree)
Trial Number

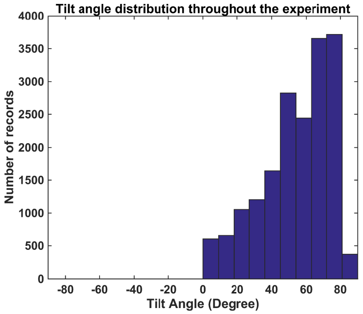
Tilt angle distribution throughout the experiment
Number of records
Tilt Angle (Degree)

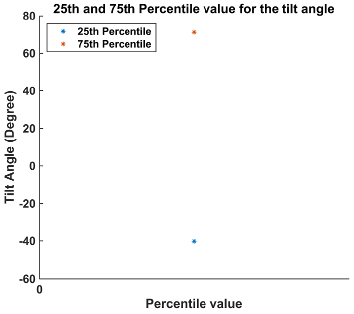
25th and 75th Percentile value for the tilt angle
25th Percentile
75th Percentile
Tilt Angle (Degree)
Percentile value

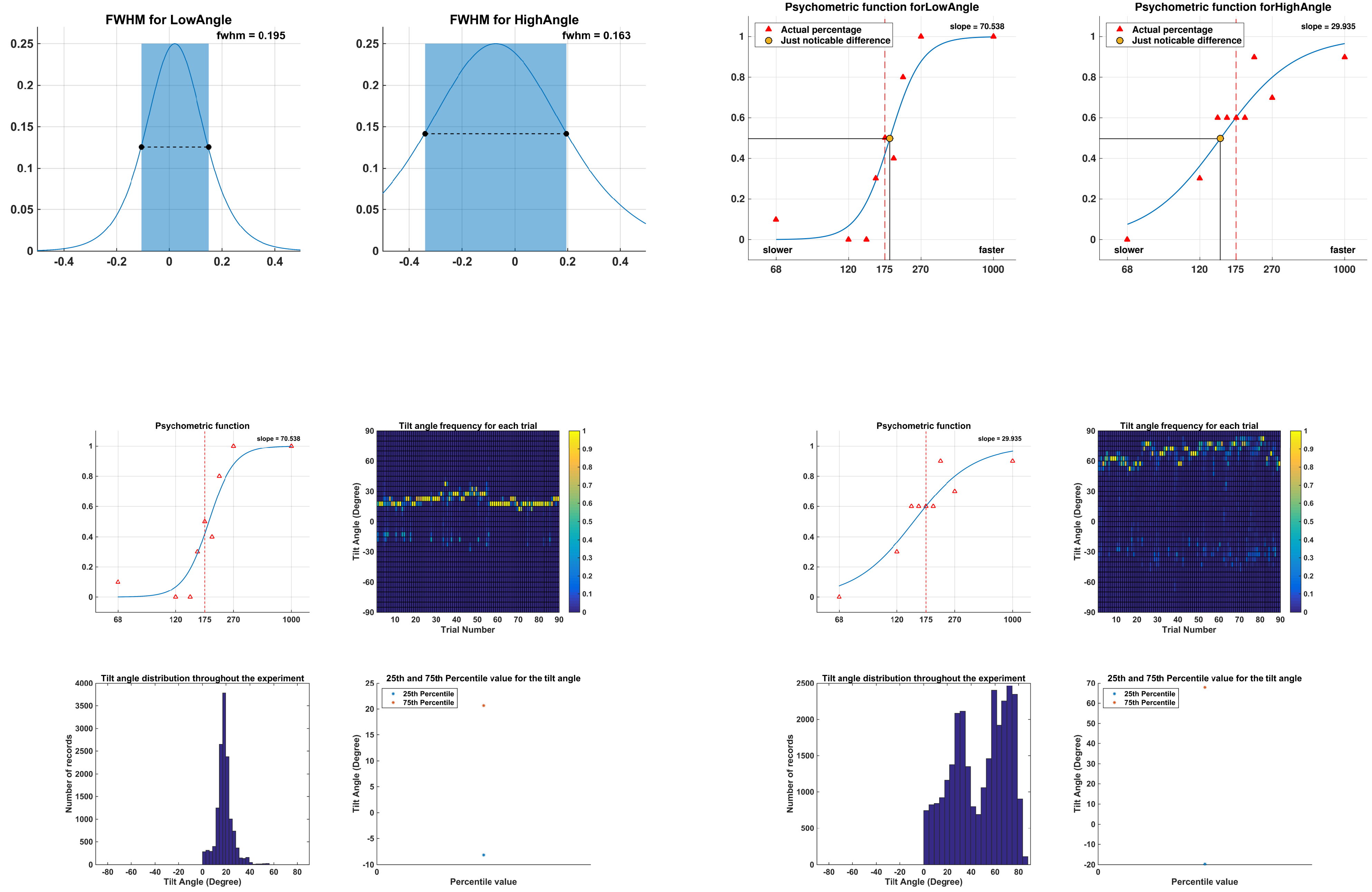
FWHM for LowAngle
fwhm = 0.195
FWHM for HighAngle
fwhm = 0.163
Psychometric function forLowAngle
Actual percentage
Just noticable difference
slope = 70.538
slower
faster
Psychometric function forHighAngle
Actual percentage
Just noticable difference
slope = 29.935
slower
faster
Psychometric function
slope = 70.538
Tilt angle frequency for each trial
Tilt Angle (Degree)
Trial Number
Psychometric function
slope = 29.935
Tilt angle frequency for each trial
Tilt Angle (Degree)
Trial Number
Tilt angle distribution throughout the experiment
Number of records
Tilt Angle (Degree)
25th and 75th Percentile value for the tilt angle
25th Percentile
75th Percentile
Tilt Angle (Degree)
Percentile value
Tilt angle distribution throughout the experiment
Number of records
Tilt Angle (Degree)
25th and 75th Percentile value for the tilt angle
25th Percentile
75th Percentile
Tilt Angle (Degree)
Percentile value

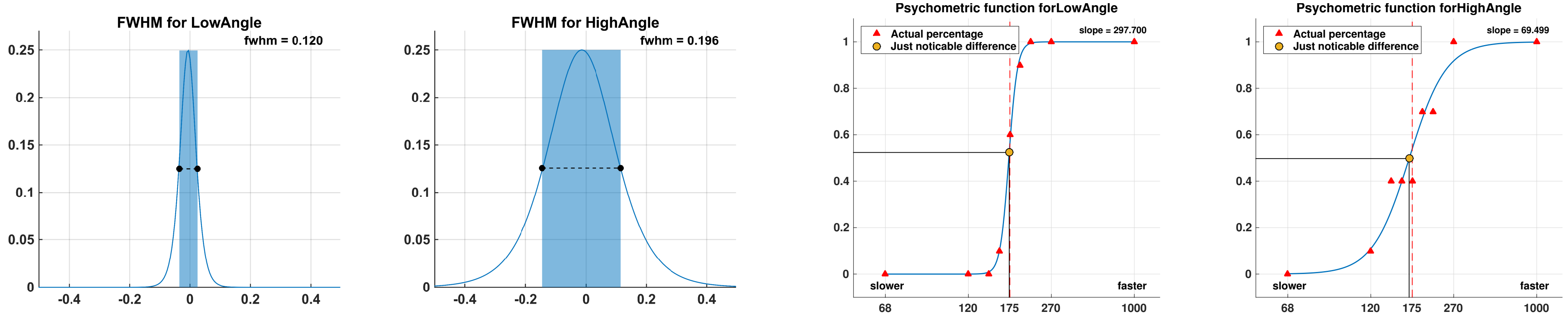
FWHM for LowAngle
fwhm = 0.120
FWHM for HighAngle
fwhm = 0.196
Psychometric function forLowAngle
Actual percentage
Just noticable difference
slope = 297.700
slower
faster
Psychometric function forHighAngle
slope = 69.499

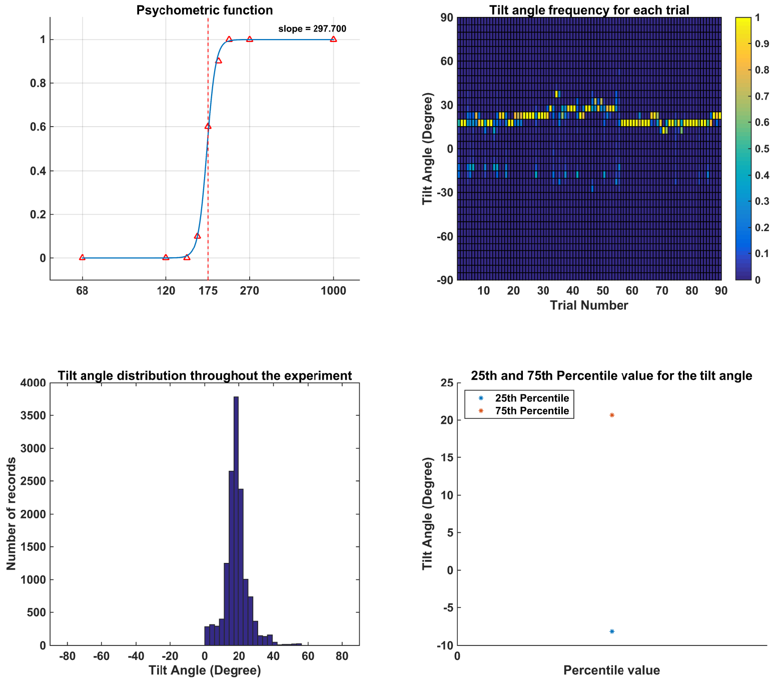
Psychometric function
slope = 297.700
Tilt angle frequency for each trial
Tilt Angle (Degree)
Trial Number
Tilt angle distribution throughout the experiment
Number of records
Tilt Angle (Degree)
25th and 75th Percentile value for the tilt angle
25th Percentile
75th Percentile
Tilt Angle (Degree)
Percentile value

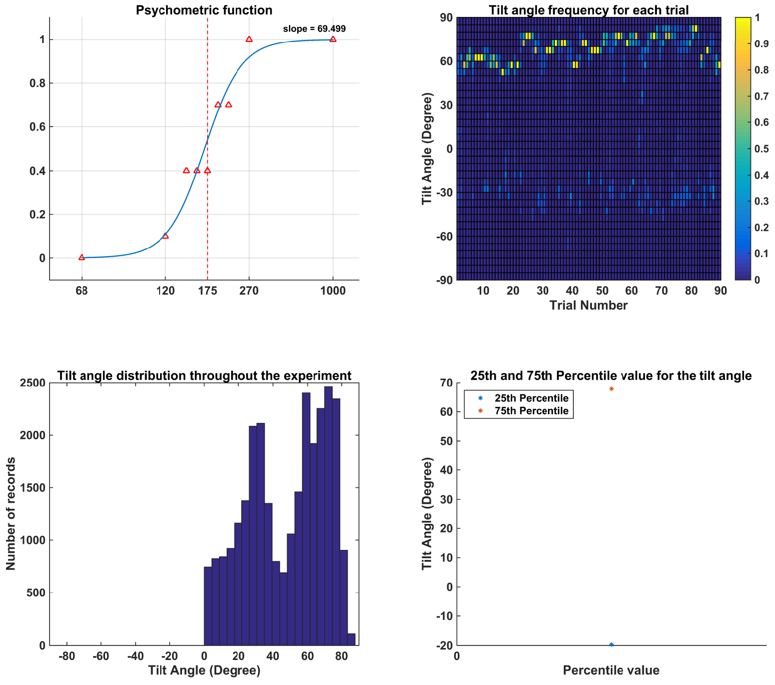
Psychometric function
slope = 69.499
Tilt angle frequency for each trial
Tilt Angle (Degree)
Trial Number
Tilt angle distribution throughout the experiment
Number of records
Tilt Angle (Degree)
25th and 75th Percentile value for the tilt angle
25th Percentile
75th Percentile
Tilt Angle (Degree)
Percentile value

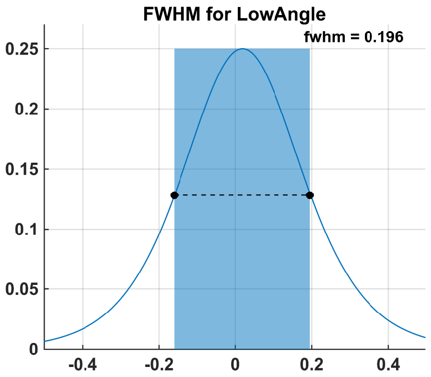
FWHM for LowAngle
fwhm = 0.196

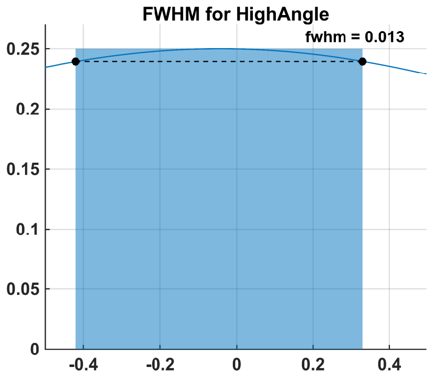
FWHM for HighAngle
fwhm = 0.013

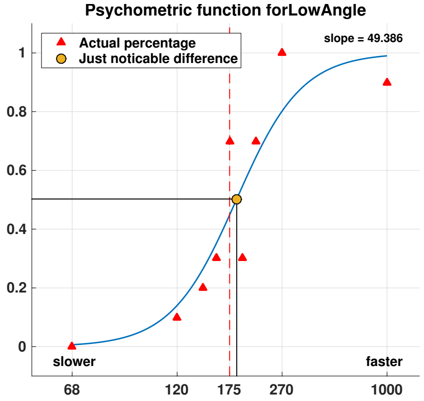
Psychometric function forLowAngle
Actual percentage
Just noticable difference
slope = 49.386
slower
faster

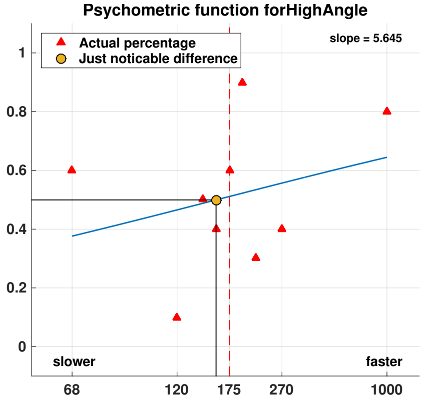
Psychometric function forHighAngle
Actual percentage
Just noticable difference
slope = 5.645
slower
faster

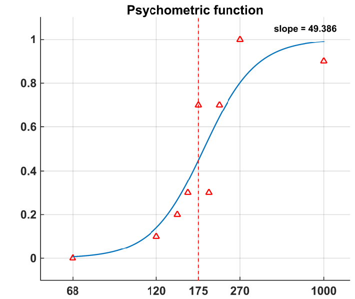
Psychometric function
slope = 49.386

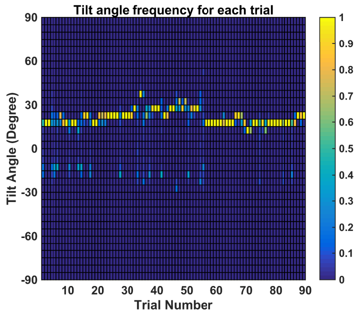
Tilt angle frequency for each trial
Tilt Angle (Degree)
Trial Number

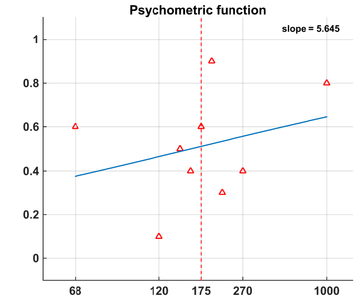
Psychometric function
slope = 5.645

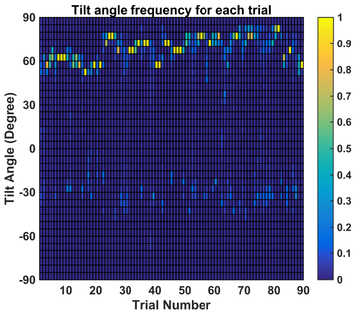
Tilt angle frequency for each trial
Tilt Angle (Degree)
Trial Number

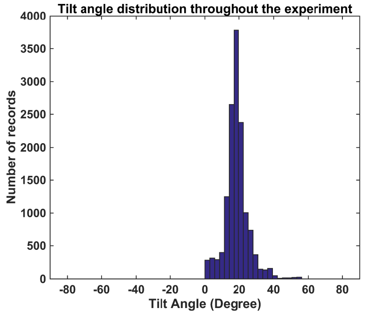
Tilt angle distribution throughout the experiment
Number of records
Tilt Angle (Degree)

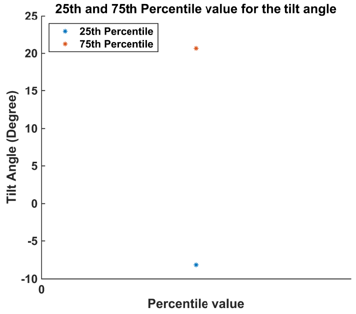
25th and 75th Percentile value for the tilt angle
25th Percentile
75th Percentile
Tilt Angle (Degree)
Percentile value

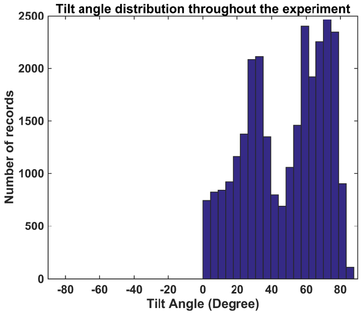
Tilt angle distribution throughout the experiment
Number of records
Tilt Angle (Degree)

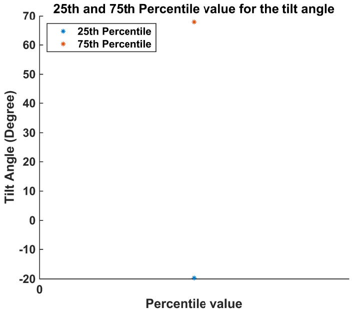
25th and 75th Percentile value for the tilt angle
25th Percentile
75th Percentile
Tilt Angle (Degree)
Percentile value

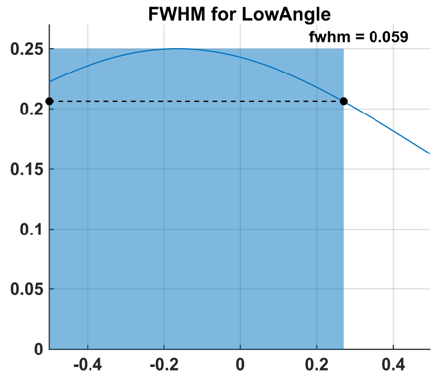
FWHM for LowAngle
fwhm = 0.059

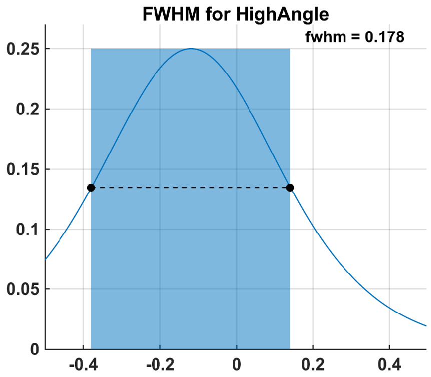
FWHM for HighAngle
fwhm = 0.178

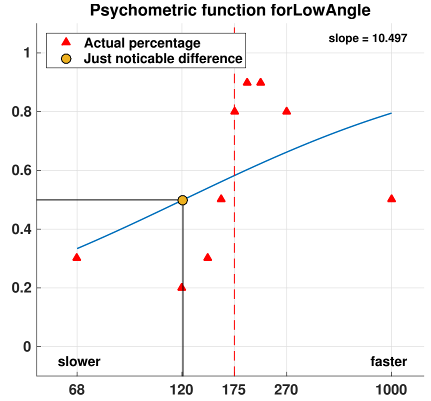
Psychometric function forLowAngle
Actual percentage
Just noticable difference
slope = 10.497
slower
faster

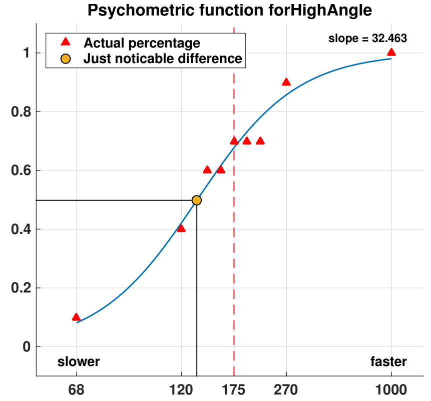
Psychometric function forHighAngle
Actual percentage
Just noticable difference
slope = 32.463
slower
faster

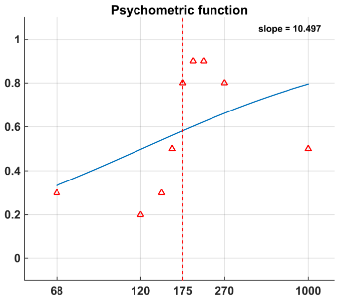
Psychometric function
slope = 10.497

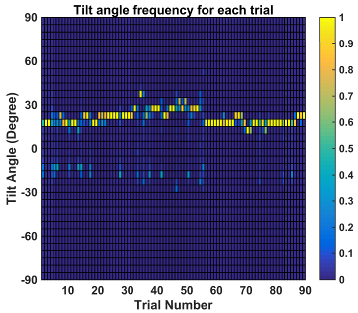
Tilt angle frequency for each trial
Tilt Angle (Degree)
Trial Number

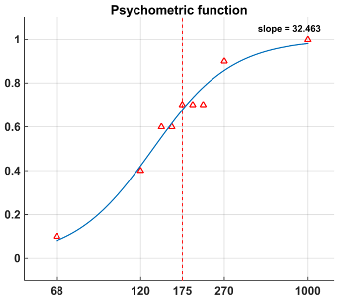
Psychometric function
slope = 32.463

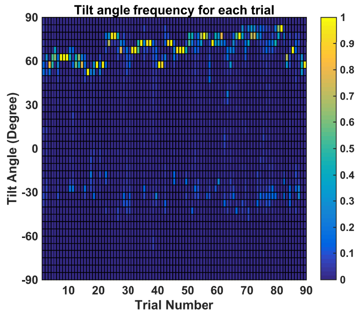
Tilt angle frequency for each trial
Tilt Angle (Degree)
Trial Number

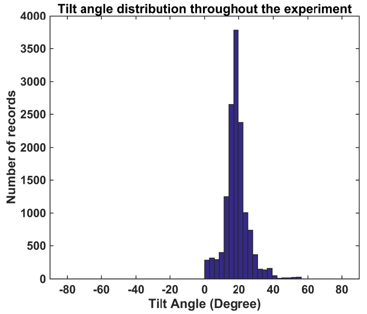
Tilt angle distribution throughout the experiment
Number of records
Tilt Angle (Degree)

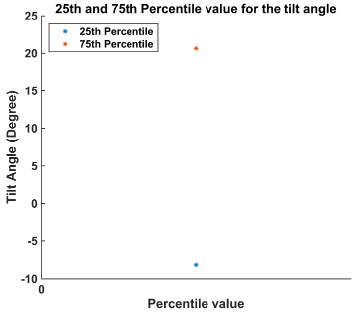
25th and 75th Percentile value for the tilt angle
25th Percentile
75th Percentile
Tilt Angle (Degree)
Percentile value

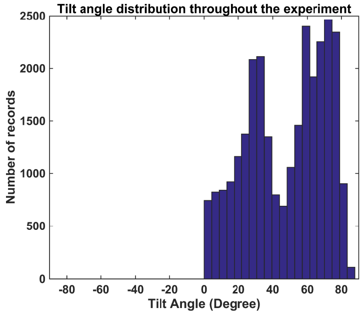
Tilt angle distribution throughout the experiment
Number of records
Tilt Angle (Degree)

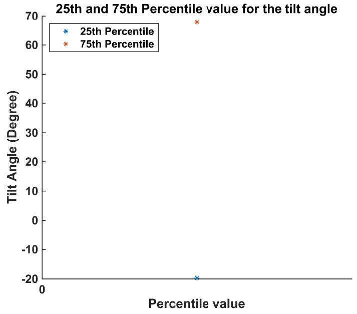
25th and 75th Percentile value for the tilt angle
25th Percentile
75th Percentile
Tilt Angle (Degree)
Percentile value

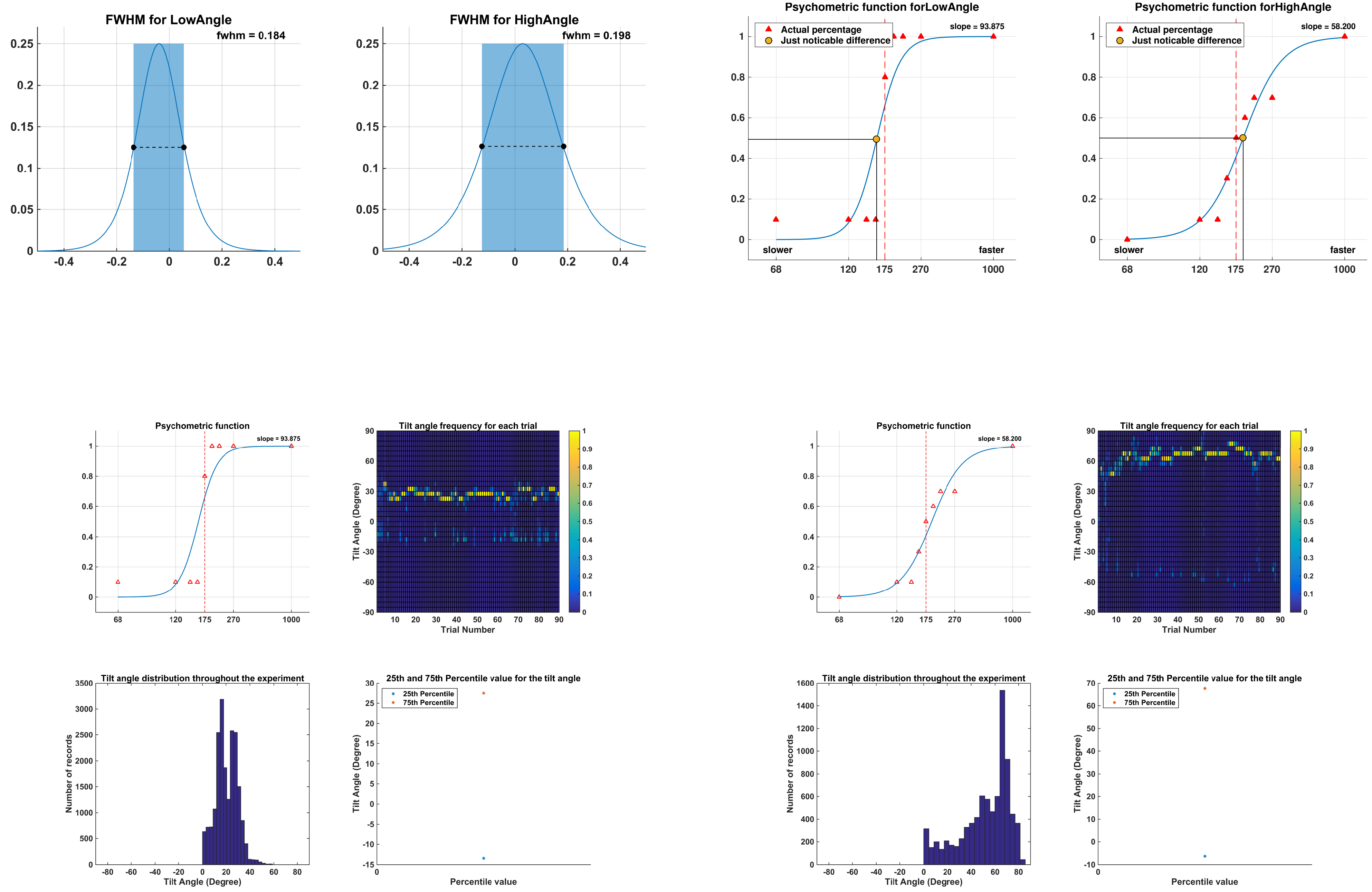

FWHM for LowAngle
fwhm = 0.184
FWHM for HighAngle
fwhm = 0.198
Psychometric function forLowAngle
Actual percentage
Just noticable difference
slope = 93.875
slower
faster
Psychometric function forHighAngle
Actual percentage
Just noticable difference
slope = 58.200
slower
faster
Psychometric function
slope = 93.875
Tilt angle frequency for each trial
Tilt Angle (Degree)
Trial Number
Psychometric function
slope = 58.200
Tilt angle frequency for each trial
Tilt Angle (Degree)
Trial Number
Tilt angle distribution throughout the experiment
Number of records
Tilt Angle (Degree)
25th and 75th Percentile value for the tilt angle
25th Percentile
75th Percentile
Tilt Angle (Degree)
Percentile value
Tilt angle distribution throughout the experiment
Number of records
Tilt Angle (Degree)
25th and 75th Percentile value for the tilt angle
25th Percentile
75th Percentile
Tilt Angle (Degree)
Percentile value

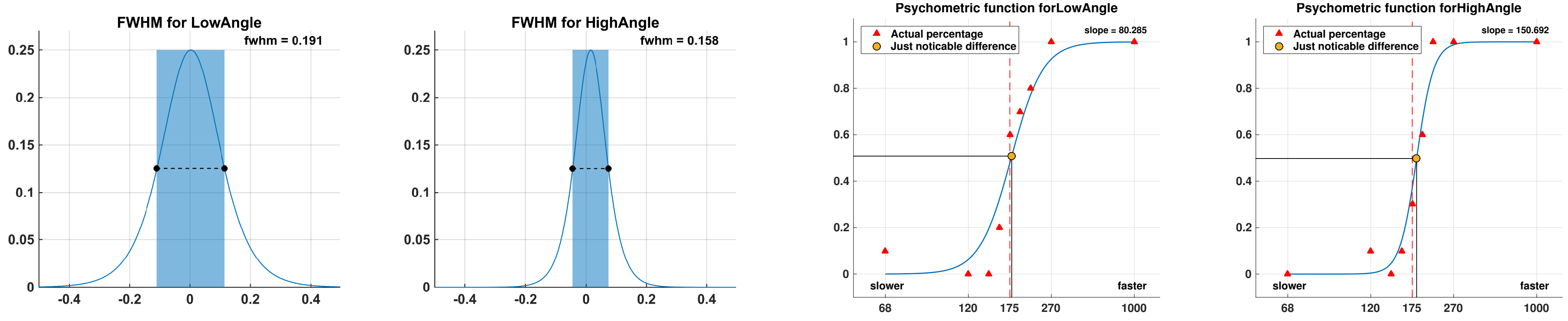
FWHM for LowAngle
fwhm = 0.191
FWHM for HighAngle
fwhm = 0.158
Psychometric function forLowAngle
Actual percentage
Just noticable difference
slope = 80.285
slower
faster
Psychometric function forHighAngle
slope = 150.692

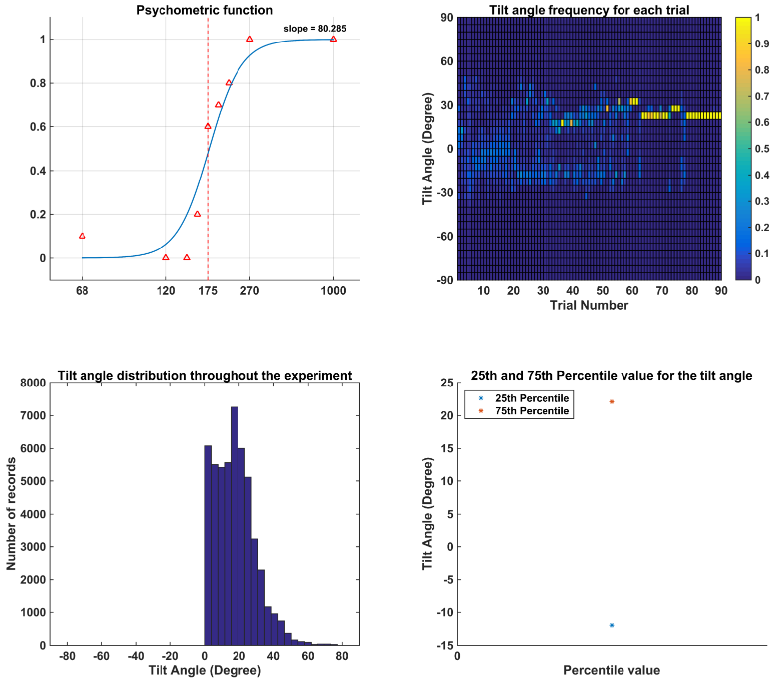
Psychometric function
slope = 80.285
Tilt angle frequency for each trial
Tilt Angle (Degree)
Trial Number
Tilt angle distribution throughout the experiment
Number of records
Tilt Angle (Degree)
25th and 75th Percentile value for the tilt angle
25th Percentile
75th Percentile
Percentile value

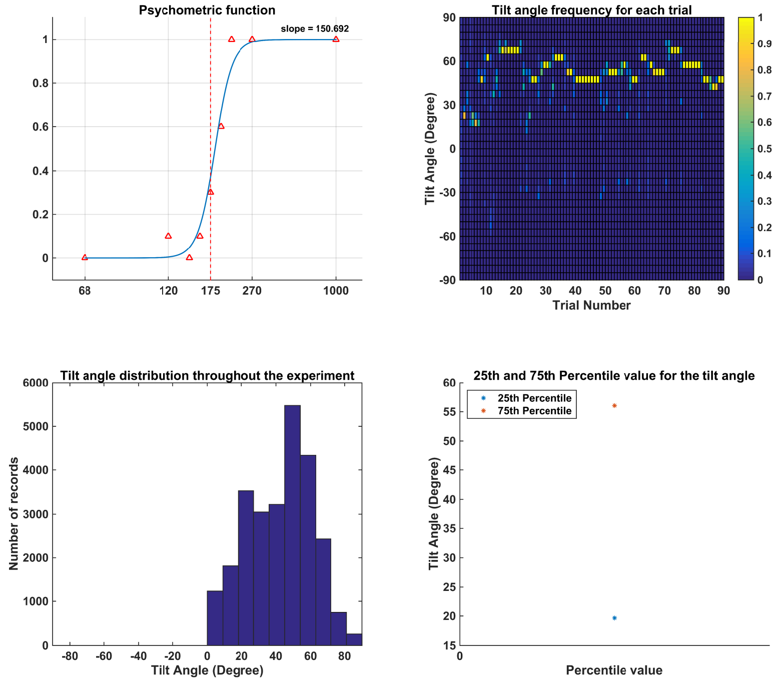
Psychometric function
slope = 150.692
Tilt angle frequency for each trial
Tilt Angle (Degree)
Trial Number
Tilt angle distribution throughout the experiment
Number of records
Tilt Angle (Degree)
25th and 75th Percentile value for the tilt angle
25th Percentile
75th Percentile
Percentile value

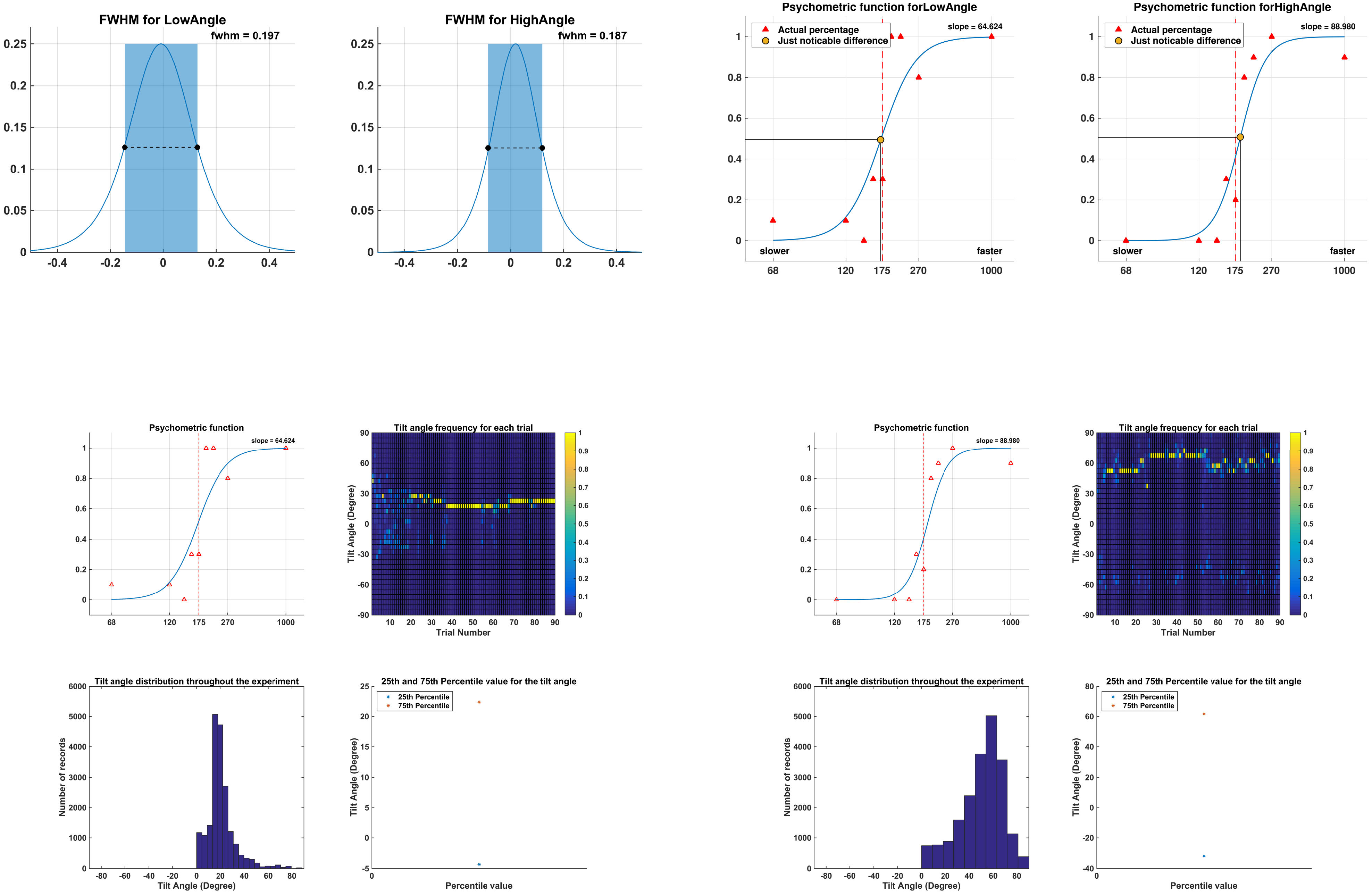
FWHM for LowAngle
fwhm = 0.197
FWHM for HighAngle
fwhm = 0.187
Psychometric function forLowAngle
Actual percentage
Just noticable difference
slope = 64.624
slower
faster
Psychometric function forHighAngle
slope = 88.980
Psychometric function
Tilt angle frequency for each trial
Tilt Angle (Degree)
Trial Number
Tilt angle distribution throughout the experiment
Number of records
25th and 75th Percentile value for the tilt angle
25th Percentile
75th Percentile
Percentile value

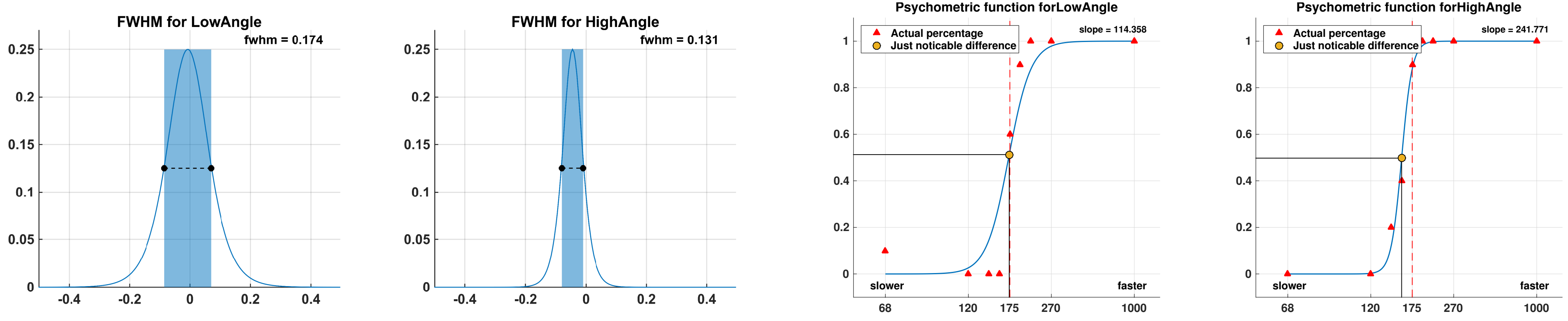
FWHM for LowAngle
fwhm = 0.174
FWHM for HighAngle
fwhm = 0.131
Psychometric function forLowAngle
Actual percentage
Just noticable difference
slope = 114.358
slower
faster
Psychometric function forHighAngle
slope = 241.771

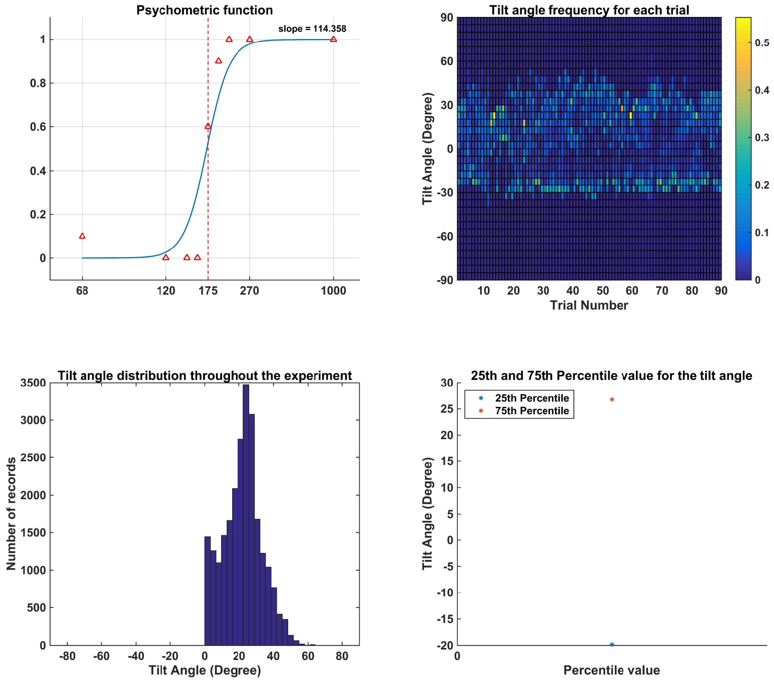
Psychometric function
slope = 114.358
Tilt angle frequency for each trial
Tilt Angle (Degree)
Trial Number
Tilt angle distribution throughout the experiment
Number of records
Tilt Angle (Degree)
25th and 75th Percentile value for the tilt angle
25th Percentile
75th Percentile
Tilt Angle (Degree)
Percentile value

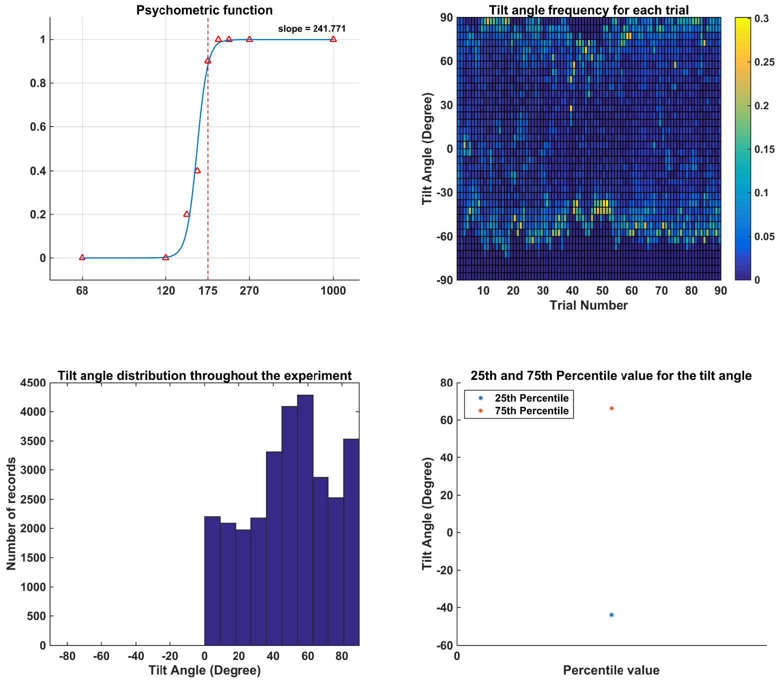
Psychometric function
slope = 241.771
Tilt angle frequency for each trial
Tilt Angle (Degree)
Trial Number
Tilt angle distribution throughout the experiment
Number of records
Tilt Angle (Degree)
25th and 75th Percentile value for the tilt angle
25th Percentile
75th Percentile
Tilt Angle (Degree)
Percentile value

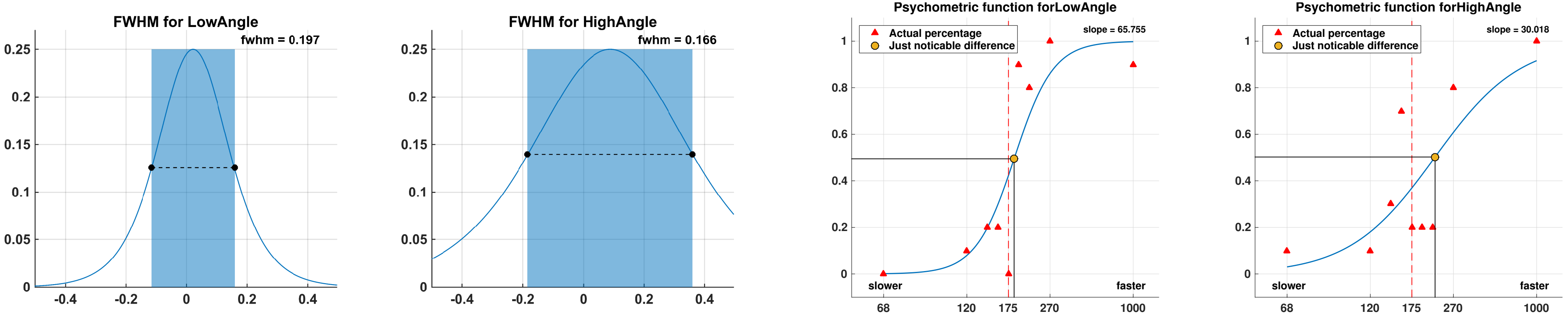
FWHM for LowAngle
fwhm = 0.197
FWHM for HighAngle
fwhm = 0.166
Psychometric function forLowAngle
Actual percentage
Just noticable difference
slope = 65.755
slower
faster
68
120
175
270
1000
Psychometric function forHighAngle
Actual percentage
Just noticable difference
slope = 30.018

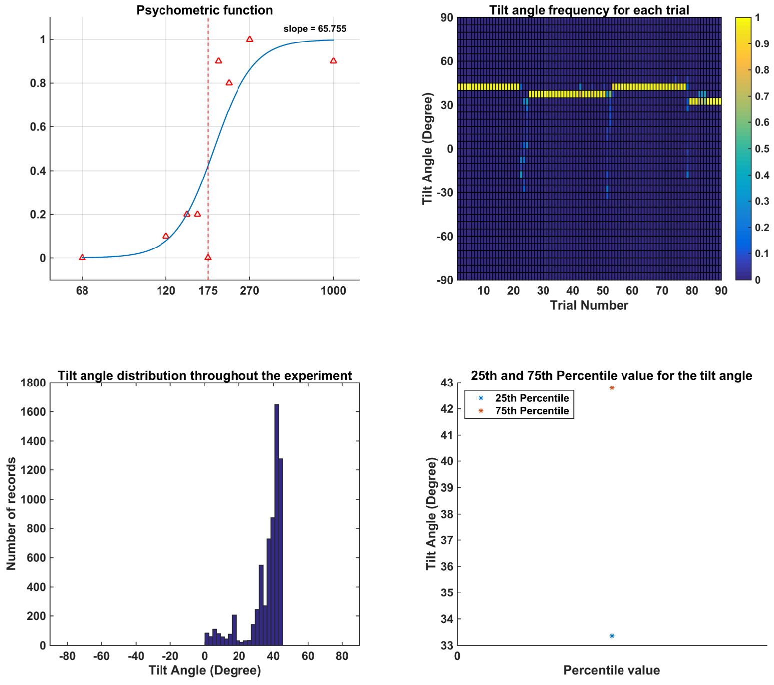
Psychometric function
slope = 65.755
Tilt angle frequency for each trial
Tilt Angle (Degree)
Trial Number
Tilt angle distribution throughout the experiment
Number of records
Tilt Angle (Degree)
25th and 75th Percentile value for the tilt angle
25th Percentile
75th Percentile
Tilt Angle (Degree)
Percentile value

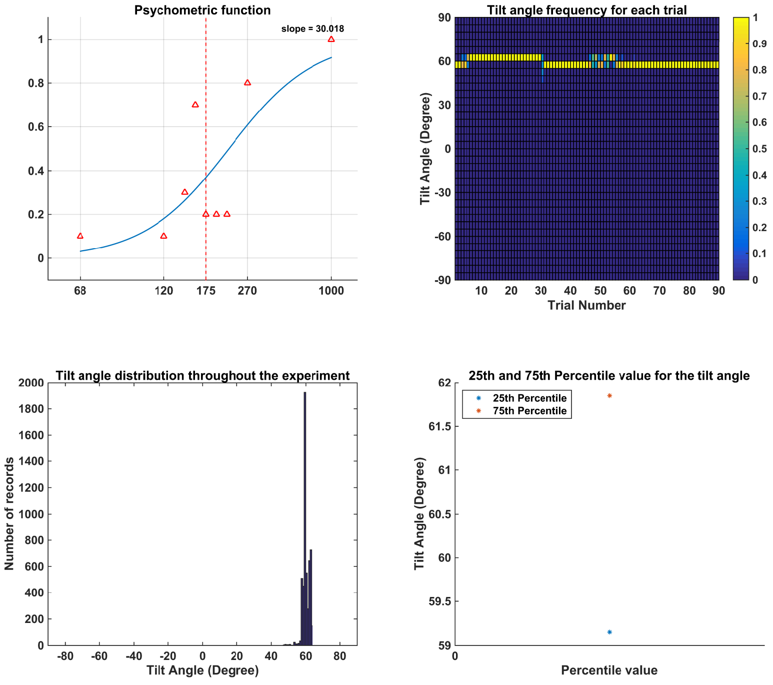
Psychometric function
slope = 30.018
Tilt angle frequency for each trial
Tilt Angle (Degree)
Trial Number
Tilt angle distribution throughout the experiment
Number of records
Tilt Angle (Degree)
25th and 75th Percentile value for the tilt angle
25th Percentile
75th Percentile
Tilt Angle (Degree)
Percentile value

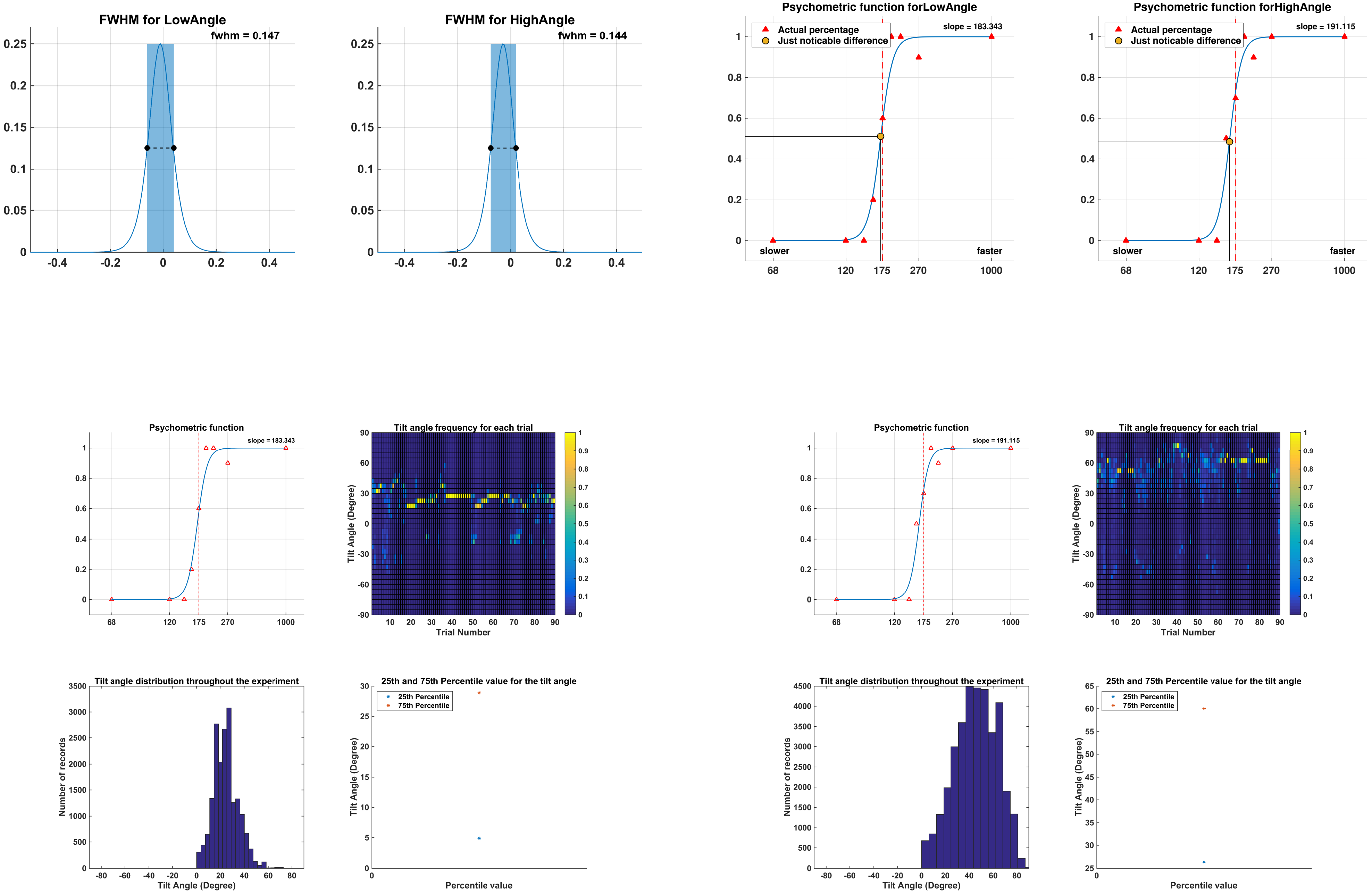
FWHM for LowAngle
fwhm = 0.147
FWHM for HighAngle
fwhm = 0.144
Psychometric function forLowAngle
Actual percentage
Just noticable difference
slope = 183.343
slower
faster
68
120
175
270
1000
Psychometric function forHighAngle
slope = 191.115
Psychometric function
slope = 183.343
Tilt angle frequency for each trial
Tilt Angle (Degree)
Trial Number
Psychometric function
slope = 191.115
Tilt angle frequency for each trial
Tilt angle distribution throughout the experiment
Number of records
Tilt Angle (Degree)
25th and 75th Percentile value for the tilt angle
25th Percentile
75th Percentile
Percentile value
Tilt angle distribution throughout the experiment
25th and 75th Percentile value for the tilt angle

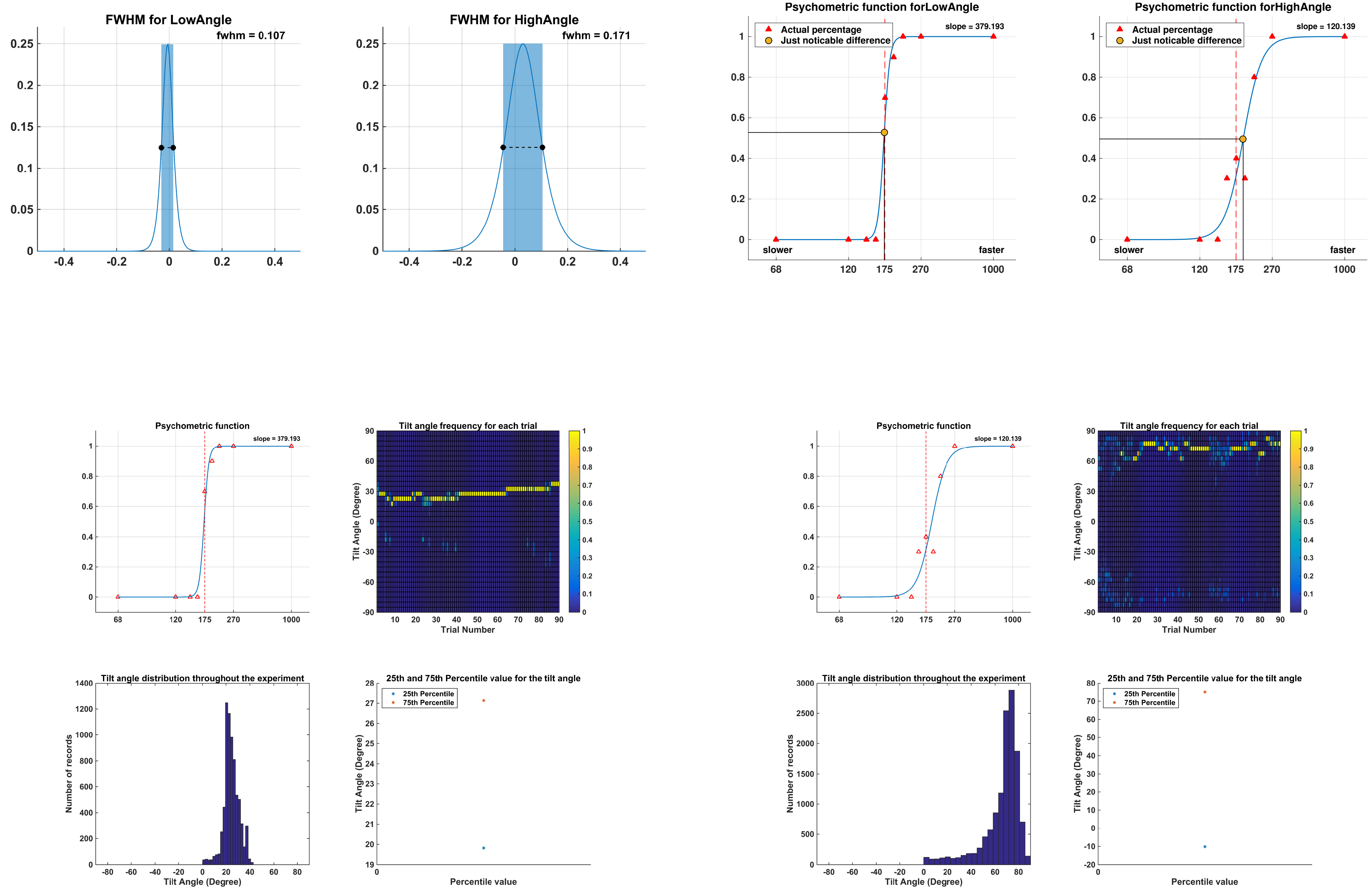
FWHM for LowAngle
fwhm = 0.107
FWHM for HighAngle
fwhm = 0.171
Psychometric function forLowAngle
Actual percentage
Just noticable difference
slope = 379.193
slower
faster
Psychometric function forHighAngle
slope = 120.139
Psychometric function
Tilt angle frequency for each trial
Tilt Angle (Degree)
Trial Number
Tilt angle distribution throughout the experiment
Number of records
25th and 75th Percentile value for the tilt angle
25th Percentile
75th Percentile
Percentile value

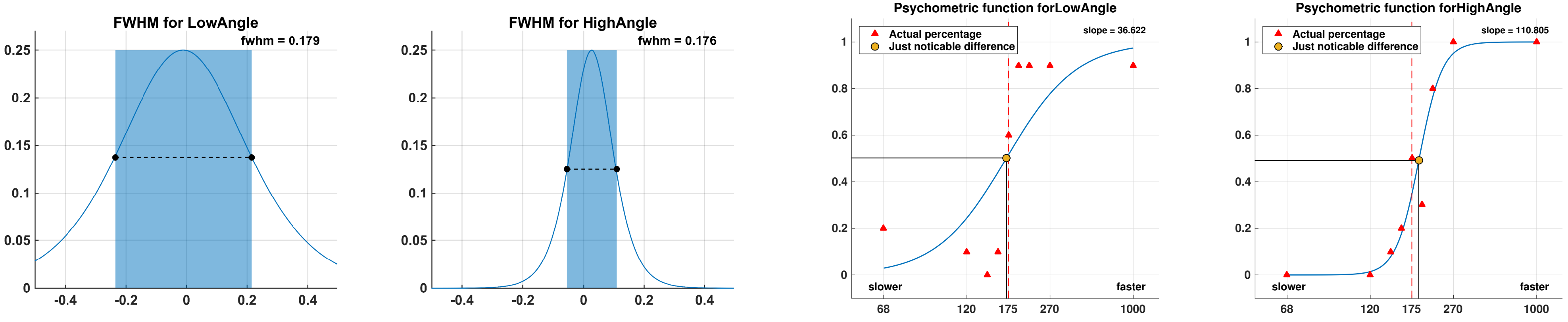

FWHM for LowAngle
fwhm = 0.179
FWHM for HighAngle
fwhm = 0.176
Psychometric function forLowAngle
slope = 36.622
Actual percentage
Just noticable difference
slower
faster
Psychometric function forHighAngle
slope = 110.805
Actual percentage
Just noticable difference
slower
faster

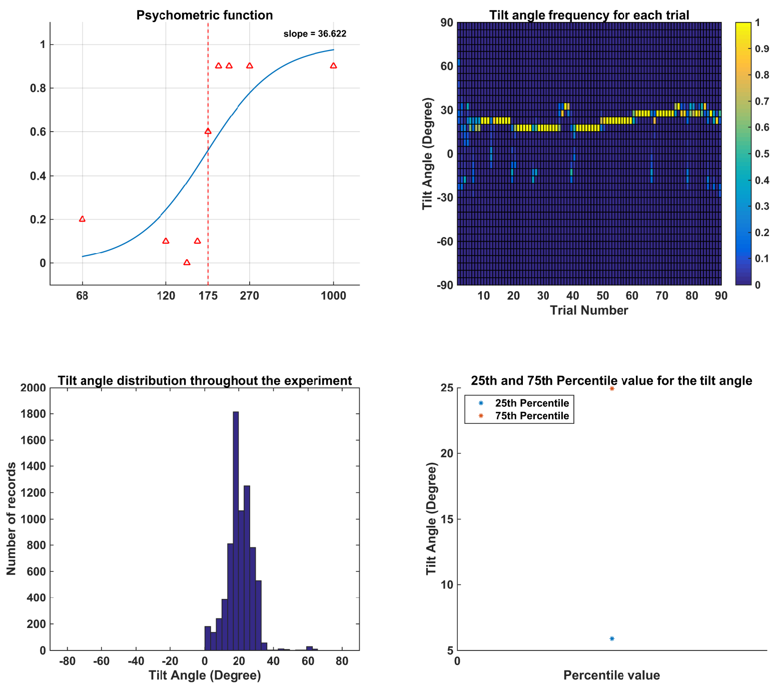

Psychometric function
slope = 36.622
Tilt angle frequency for each trial
Tilt Angle (Degree)
Trial Number
Tilt angle distribution throughout the experiment
Number of records
Tilt Angle (Degree)
25th and 75th Percentile value for the tilt angle
25th Percentile
75th Percentile
Tilt Angle (Degree)
Percentile value

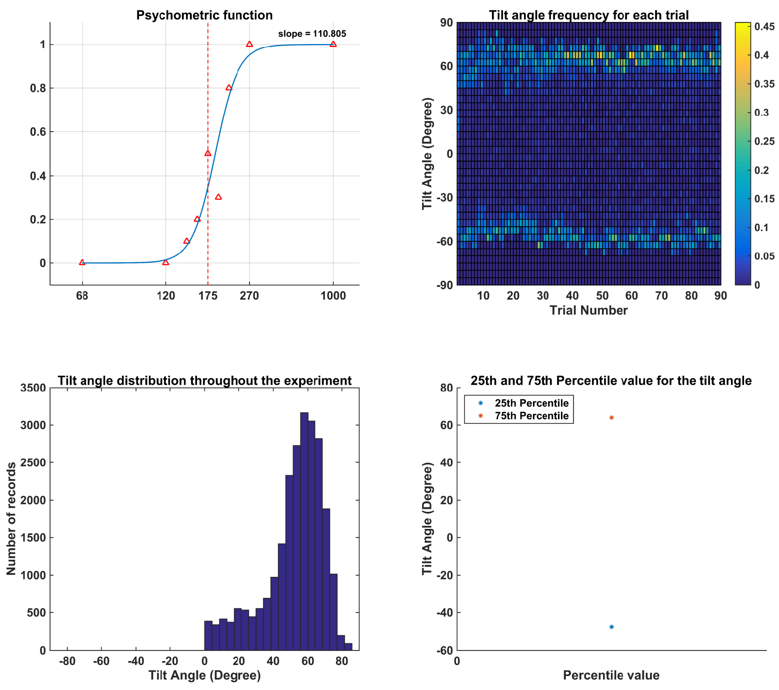

Psychometric function
slope = 110.805
Tilt angle frequency for each trial
Tilt Angle (Degree)
Trial Number
Tilt angle distribution throughout the experiment
Number of records
Tilt Angle (Degree)
25th and 75th Percentile value for the tilt angle
25th Percentile
75th Percentile
Tilt Angle (Degree)
Percentile value

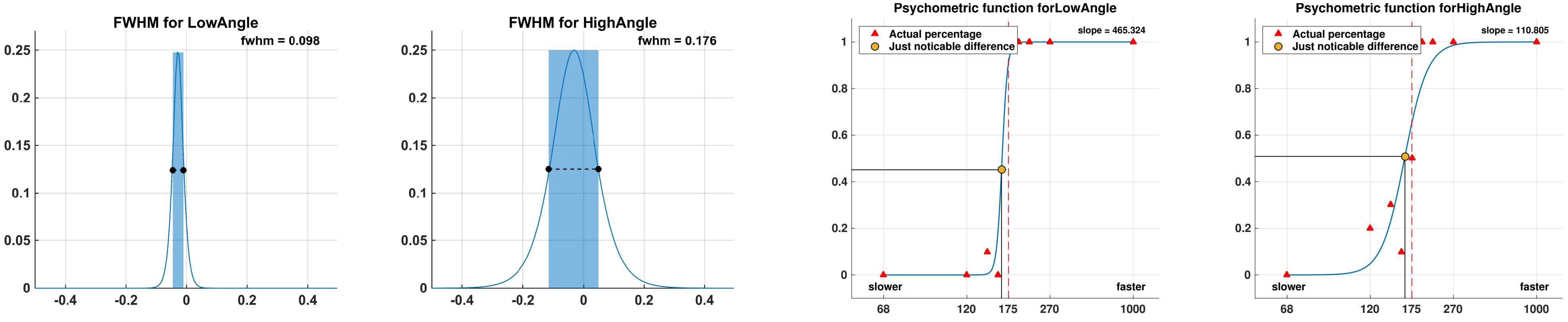

FWHM for LowAngle
fwhm = 0.098
FWHM for HighAngle
fwhm = 0.176
Psychometric function forLowAngle
Actual percentage
Just noticable difference
slope = 465.324
slower
faster
Psychometric function forHighAngle
slope = 110.805

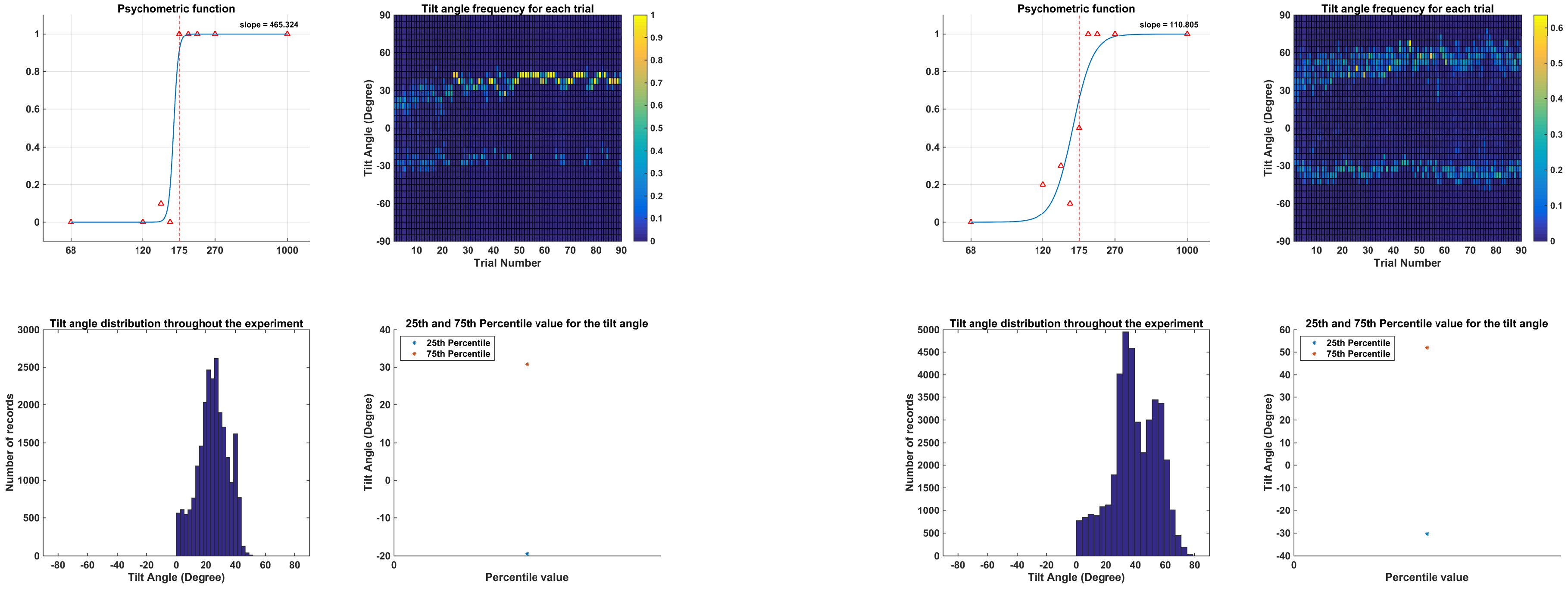

Psychometric function
slope = 465.324
Tilt angle frequency for each trial
Tilt Angle (Degree)
Trial Number
Psychometric function
slope = 110.805
Tilt angle frequency for each trial
Tilt Angle (Degree)
Trial Number
Tilt angle distribution throughout the experiment
Number of records
Tilt Angle (Degree)
25th and 75th Percentile value for the tilt angle
25th Percentile
75th Percentile
Tilt Angle (Degree)
Percentile value
Tilt angle distribution throughout the experiment
Number of records
Tilt Angle (Degree)
25th and 75th Percentile value for the tilt angle
25th Percentile
75th Percentile
Tilt Angle (Degree)
Percentile value

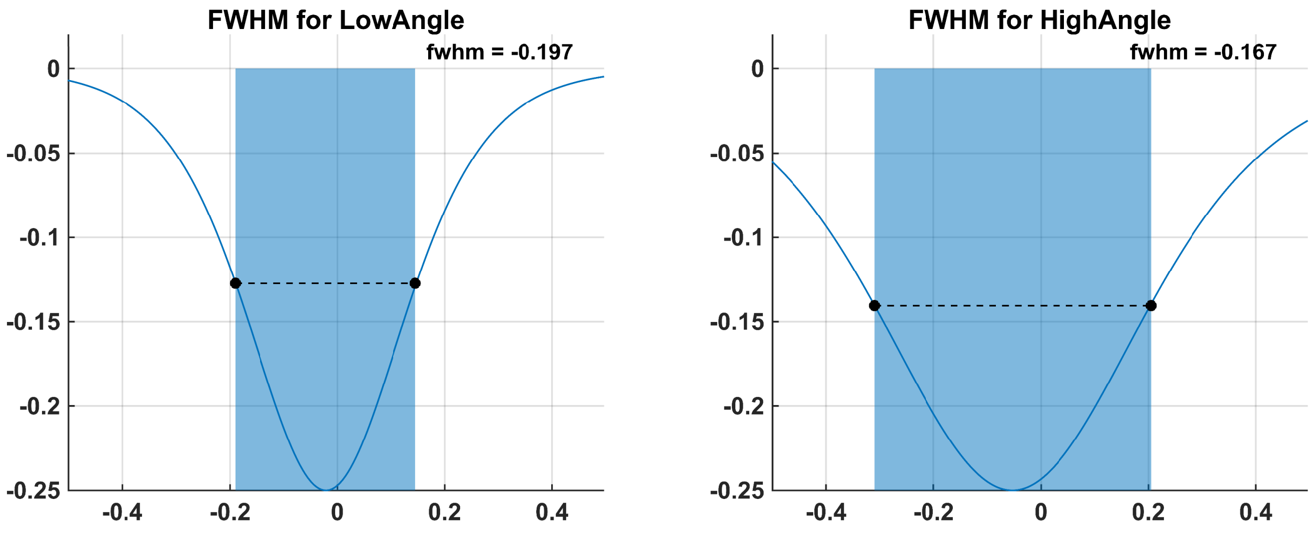

Individual participant data for friction group

Individual data for each participant from the Inverse group, namely the group which believes slower objects are heavier in both environments. This page is the group average. From page 24-34, each page corresponds to the data from each participant.

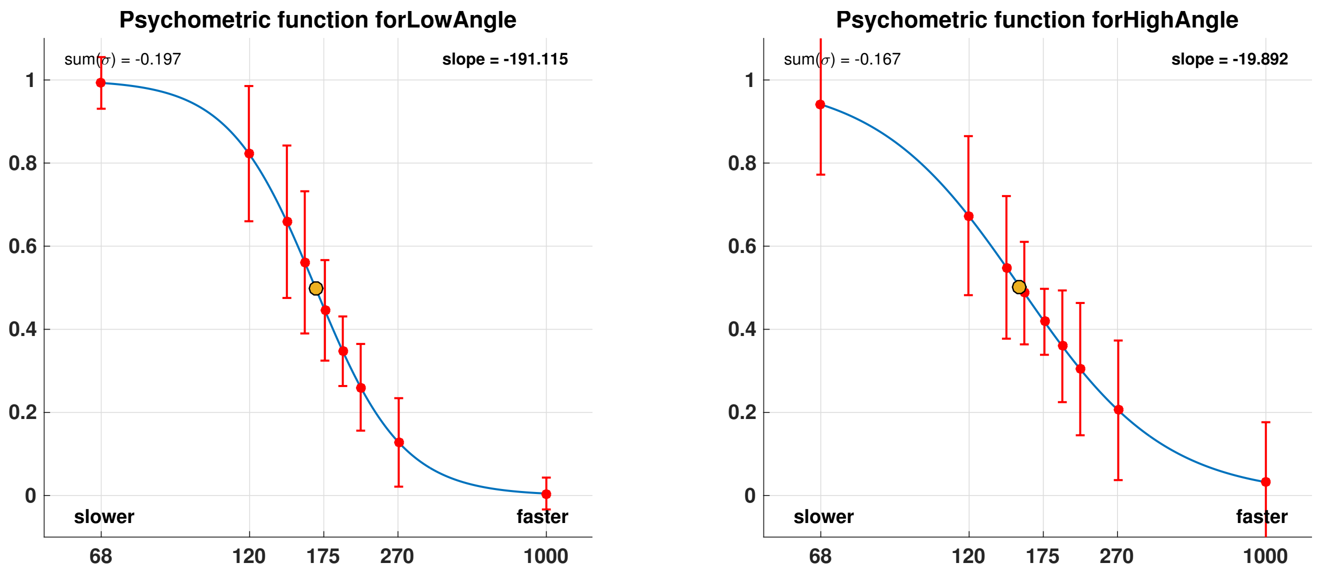

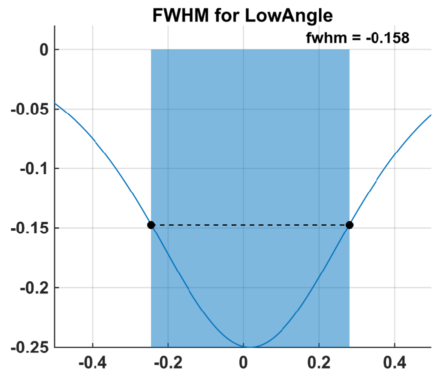
FWHM for LowAngle
fwhm = -0.158

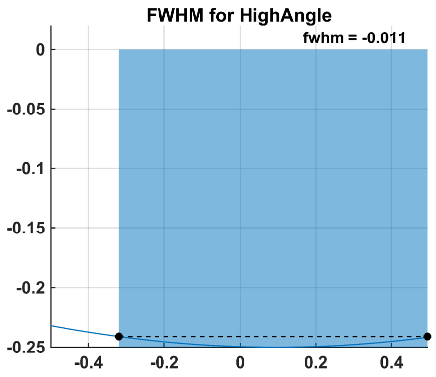
FWHM for HighAngle
fwhm = -0.011

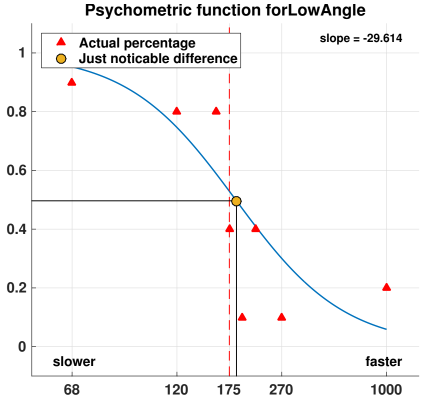
Psychometric function forLowAngle
Actual percentage
Just noticable difference
slope = -29.614
slower
faster
68
120
175
270
1000

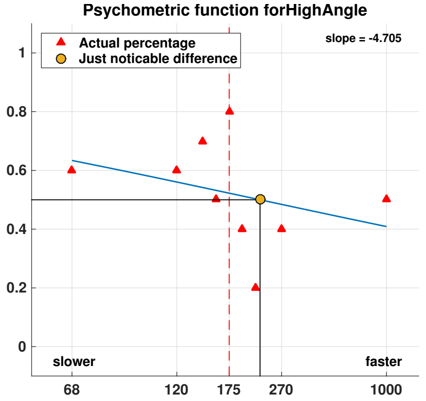
Psychometric function forHighAngle
Actual percentage
Just noticable difference
slope = -4.705
slower
faster
68
120
175
270
1000

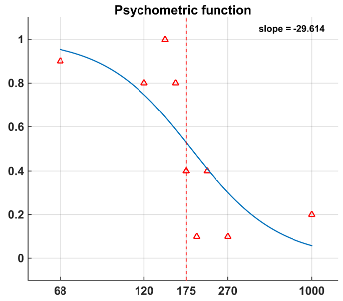
Psychometric function
slope = -29.614

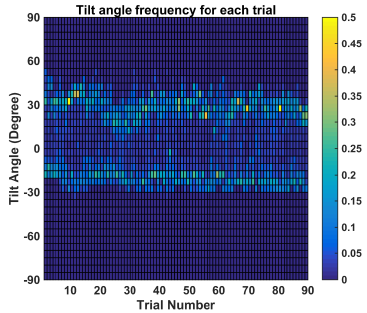
Tilt angle frequency for each trial
Tilt Angle (Degree)
Trial Number

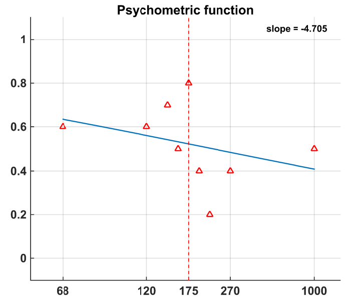
Psychometric function
slope = -4.705

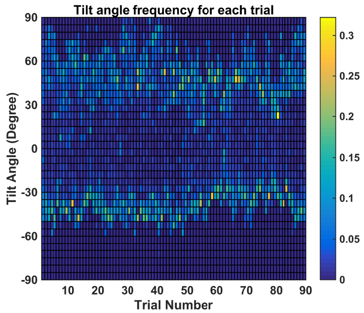
Tilt angle frequency for each trial
Tilt Angle (Degree)
Trial Number

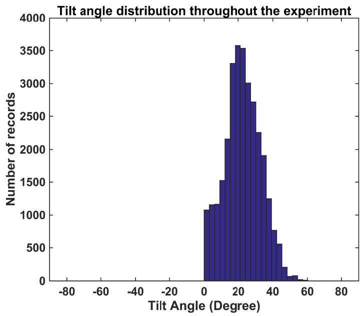
Tilt angle distribution throughout the experiment
Number of records
Tilt Angle (Degree)

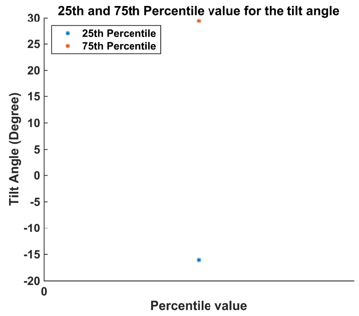
25th and 75th Percentile value for the tilt angle
25th Percentile
75th Percentile
Tilt Angle (Degree)
Percentile value

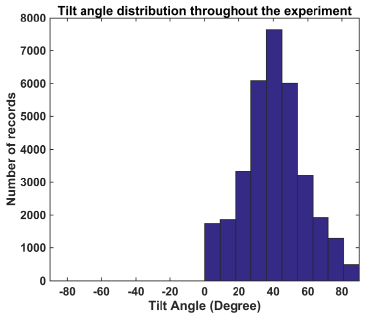
Tilt angle distribution throughout the experiment
Number of records
Tilt Angle (Degree)

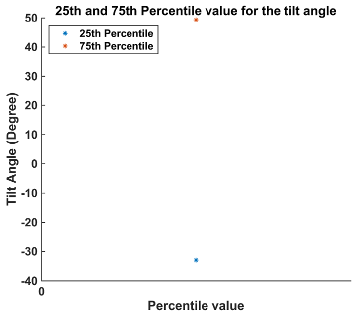
25th and 75th Percentile value for the tilt angle
25th Percentile
75th Percentile
Tilt Angle (Degree)
Percentile value

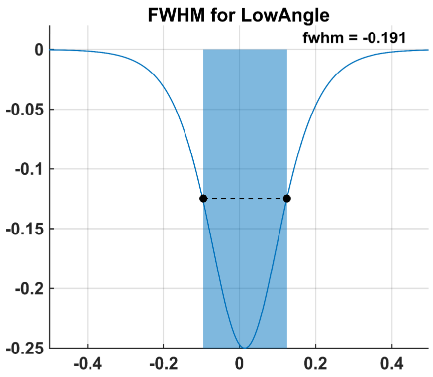
FWHM for LowAngle
fwhm = -0.191

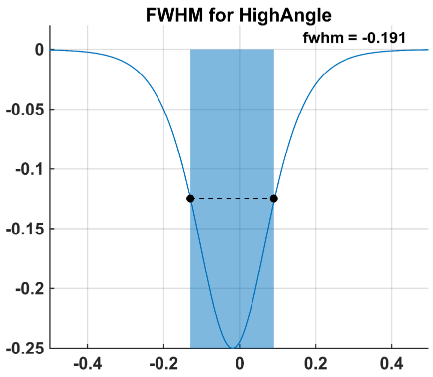
FWHM for HighAngle
fwhm = -0.191

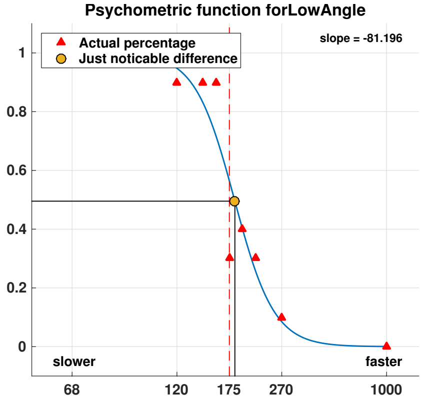
Psychometric function forLowAngle
Actual percentage
Just noticable difference
slope = -81.196
slower
faster

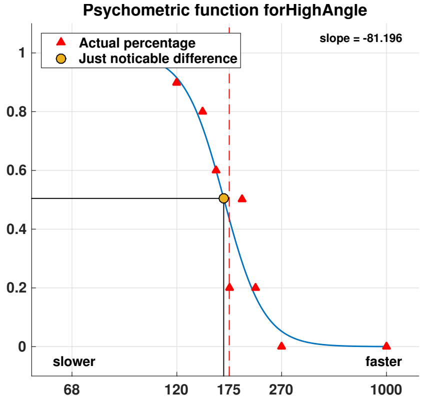
Psychometric function forHighAngle
Actual percentage
Just noticable difference
slope = -81.196
slower
faster

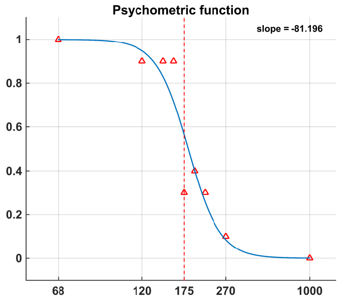
Psychometric function
slope = -81.196

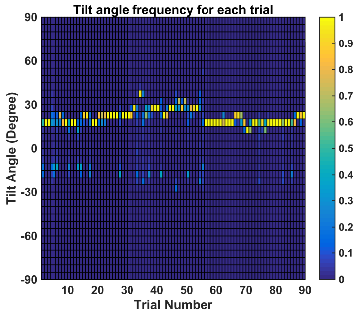
Tilt angle frequency for each trial
Tilt Angle (Degree)
Trial Number

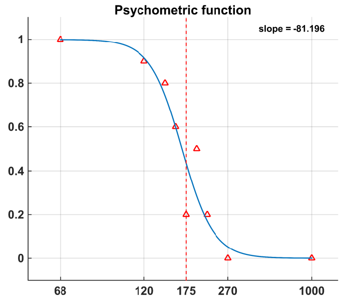
Psychometric function
slope = -81.196

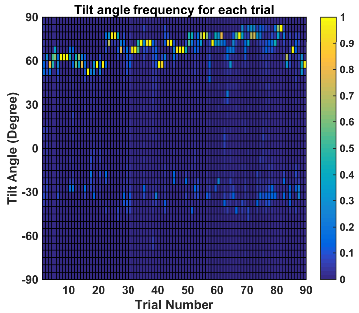
Tilt angle frequency for each trial
Tilt Angle (Degree)
Trial Number

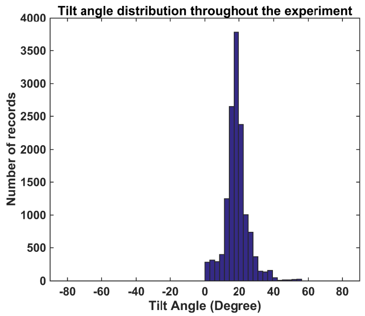
Tilt angle distribution throughout the experiment
Number of records
Tilt Angle (Degree)

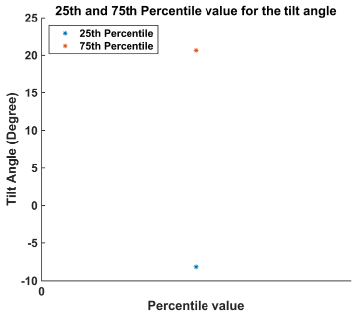
25th and 75th Percentile value for the tilt angle
25th Percentile
75th Percentile
Tilt Angle (Degree)
Percentile value

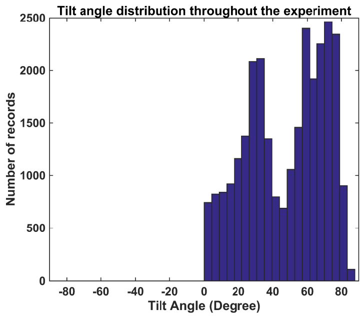
Tilt angle distribution throughout the experiment
Number of records
Tilt Angle (Degree)

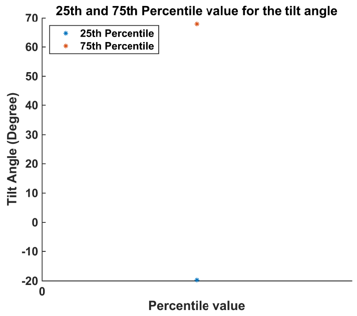
25th and 75th Percentile value for the tilt angle
25th Percentile
75th Percentile
Tilt Angle (Degree)
Percentile value

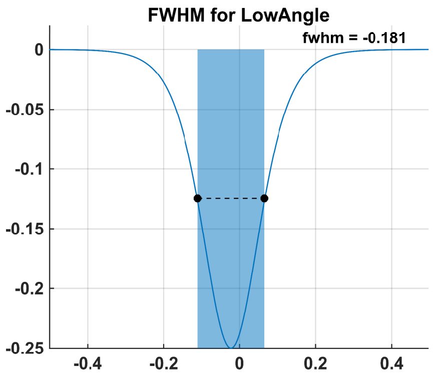
FWHM for LowAngle
fwhm = -0.181

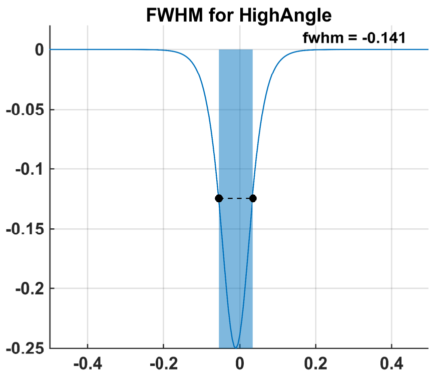
FWHM for HighAngle
fwhm = -0.141

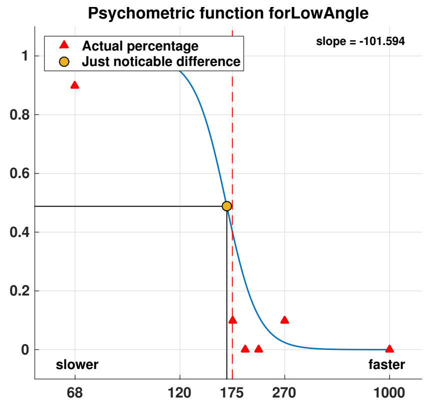
Psychometric function forLowAngle
Actual percentage
Just noticable difference
slope = -101.594
slower
faster
68
120
175
270
1000

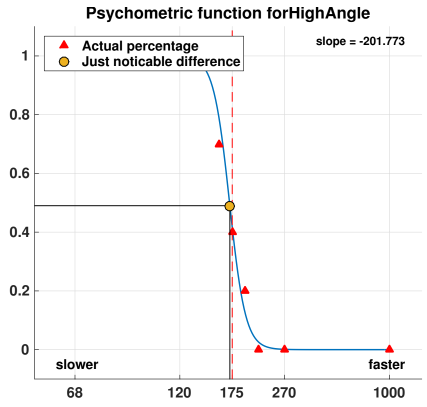
Psychometric function forHighAngle
Actual percentage
Just noticable difference
slope = -201.773
slower
faster
68
120
175
270
1000

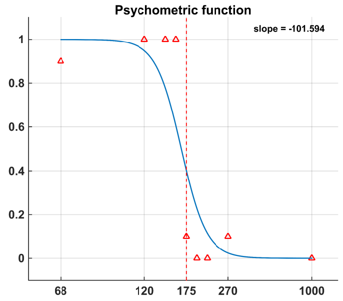
Psychometric function
slope = -101.594

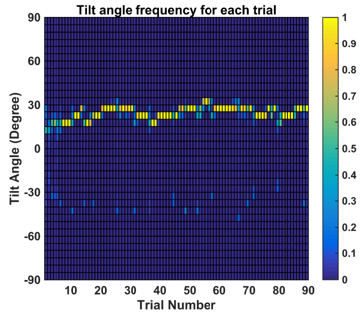
Tilt angle frequency for each trial
Tilt Angle (Degree)
Trial Number

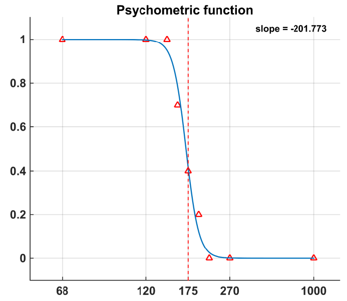
Psychometric function
slope = -201.773

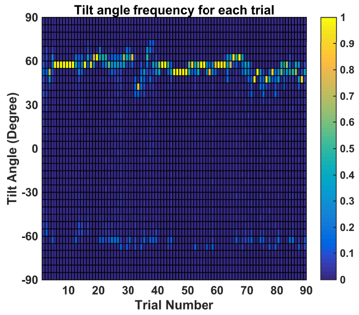
Tilt angle frequency for each trial
Tilt Angle (Degree)
Trial Number

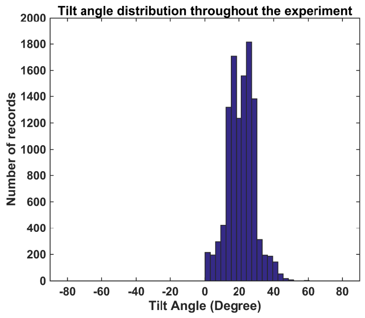
Tilt angle distribution throughout the experiment
Number of records
Tilt Angle (Degree)

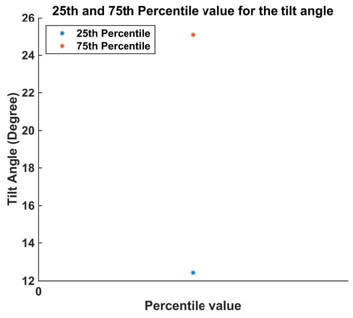
25th and 75th Percentile value for the tilt angle
25th Percentile
75th Percentile
Tilt Angle (Degree)
Percentile value

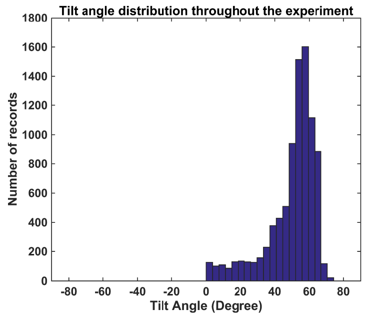
Tilt angle distribution throughout the experiment
Number of records
Tilt Angle (Degree)

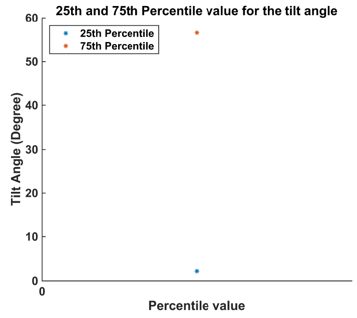
25th and 75th Percentile value for the tilt angle
25th Percentile
75th Percentile
Tilt Angle (Degree)
Percentile value

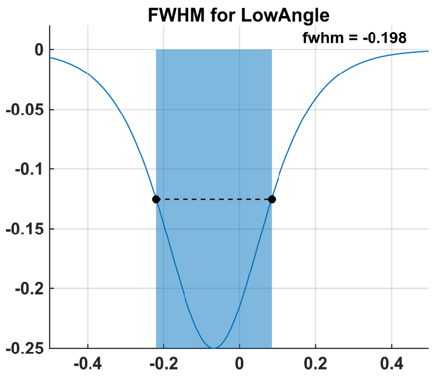
FWHM for LowAngle
fwhm = -0.198
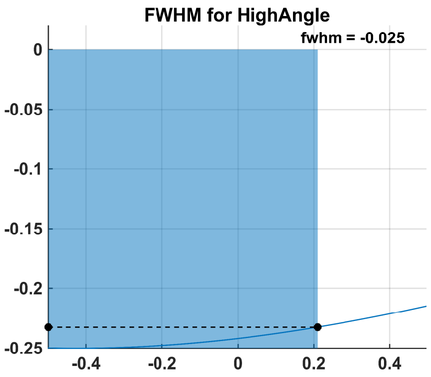
FWHM for HighAngle
fwhm = -0.025
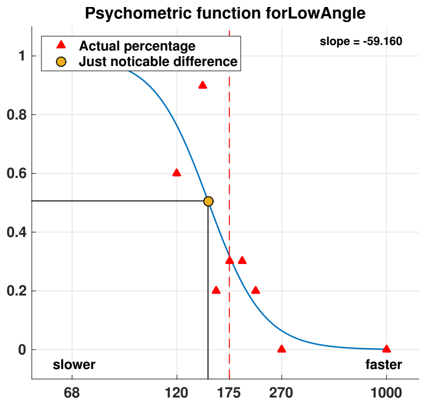
Psychometric function forLowAngle
Actual percentage
Just noticable difference
slope = -59.160
slower
faster
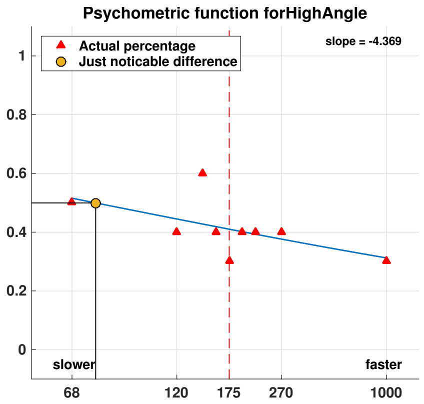
Psychometric function forHighAngle
Actual percentage
Just noticable difference
slope = -4.369
slower
faster
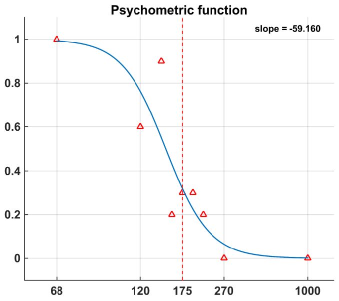
Psychometric function
slope = -59.160
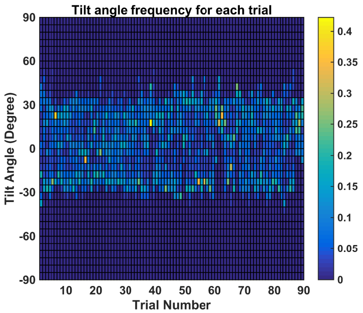
Tilt angle frequency for each trial
Tilt Angle (Degree)
Trial Number
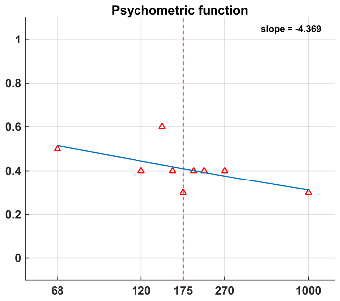
Psychometric function
slope = -4.369
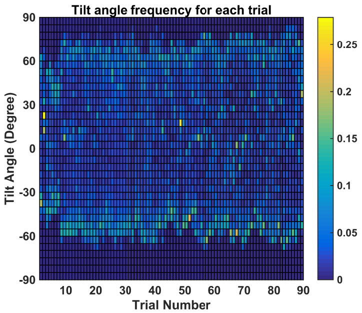
Tilt angle frequency for each trial
Tilt Angle (Degree)
Trial Number
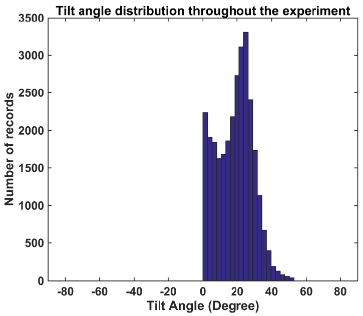
Tilt angle distribution throughout the experiment
Number of records
Tilt Angle (Degree)
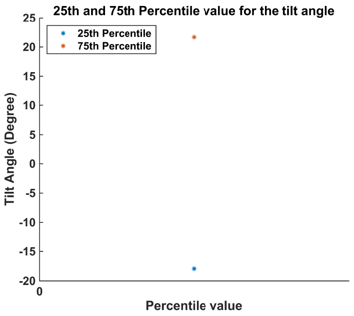
25th and 75th Percentile value for the tilt angle
25th Percentile
75th Percentile
Tilt Angle (Degree)
Percentile value
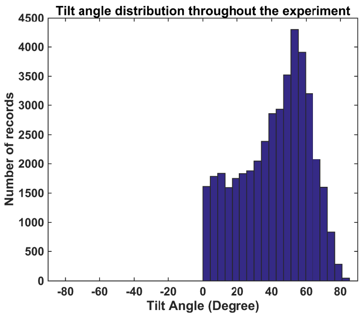
Tilt angle distribution throughout the experiment
Number of records
Tilt Angle (Degree)
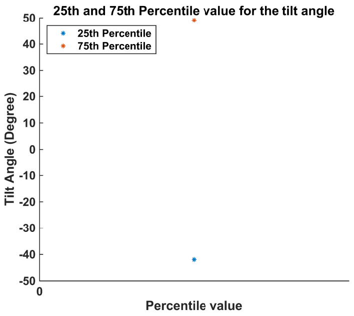
25th and 75th Percentile value for the tilt angle
25th Percentile
75th Percentile
Tilt Angle (Degree)
Percentile value

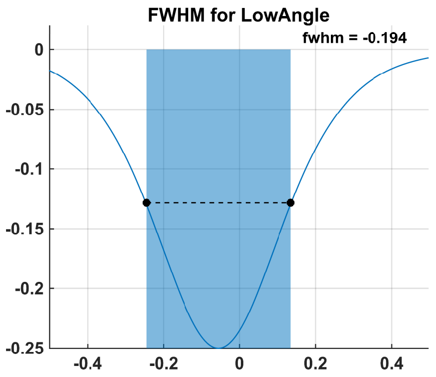
FWHM for LowAngle
fwhm = -0.194

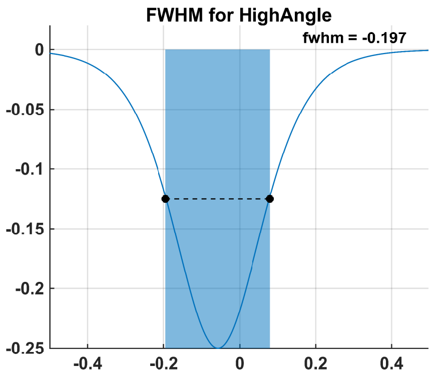
FWHM for HighAngle
fwhm = -0.197

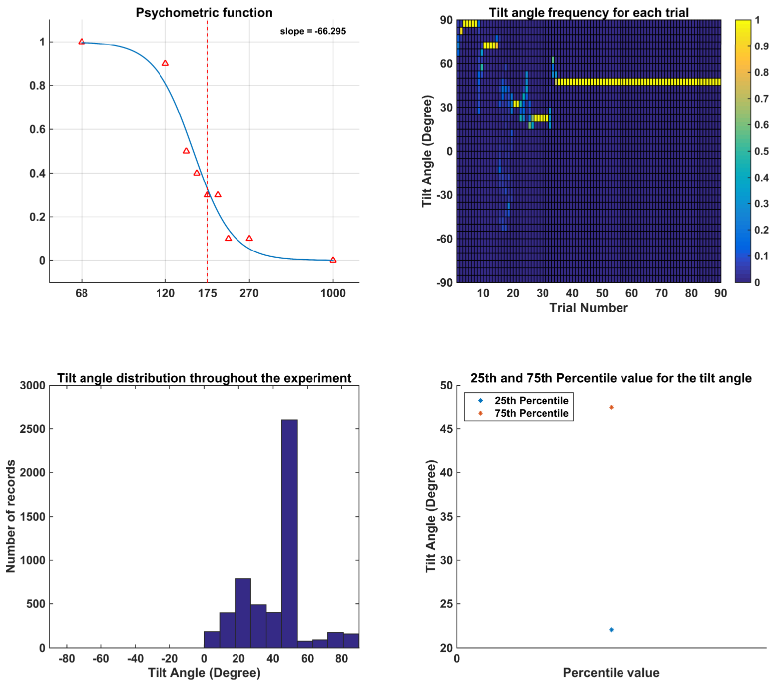
Psychometric function
slope = -66.295
Tilt angle frequency for each trial
Tilt Angle (Degree)
Trial Number
Tilt angle distribution throughout the experiment
Number of records
Tilt Angle (Degree)
25th and 75th Percentile value for the tilt angle
25th Percentile
75th Percentile
Tilt Angle (Degree)
Percentile value

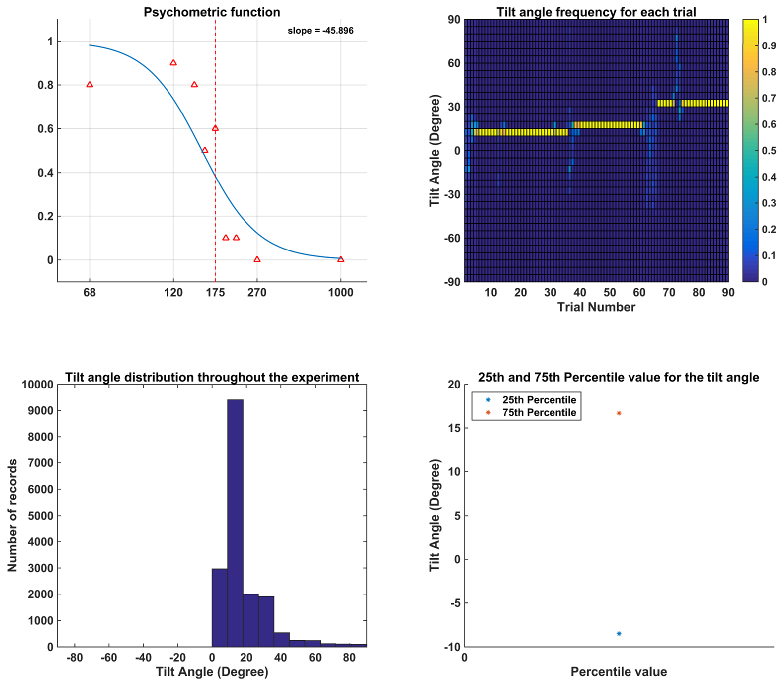
Psychometric function
slope = -45.896
Tilt angle frequency for each trial
Tilt Angle (Degree)
Trial Number
Tilt angle distribution throughout the experiment
Number of records
Tilt Angle (Degree)
25th and 75th Percentile value for the tilt angle
25th Percentile
75th Percentile
Tilt Angle (Degree)
Percentile value

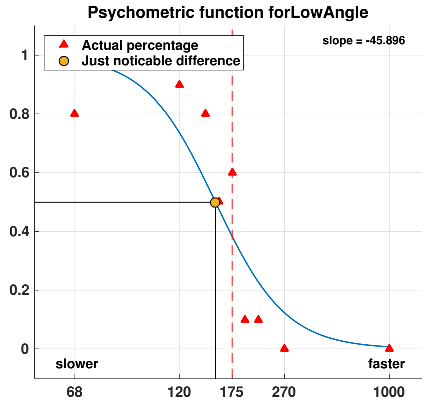
Psychometric function forLowAngle
Actual percentage
Just noticable difference
slope = -45.896
slower
faster

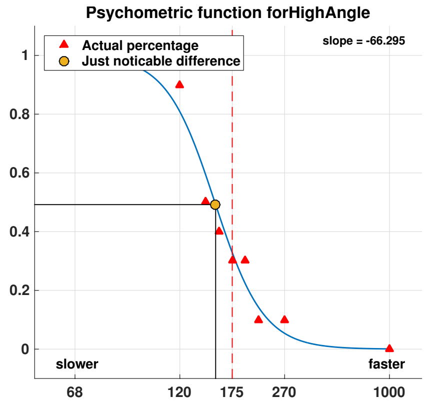
Psychometric function forHighAngle
Actual percentage
Just noticable difference
slope = -66.295
slower
faster

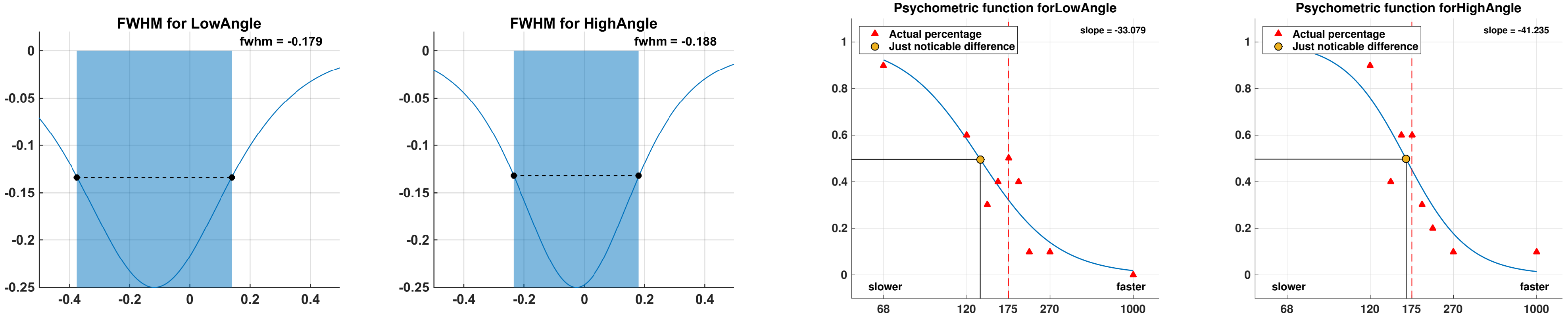
FWHM for LowAngle
fwhm = -0.179
FWHM for HighAngle
fwhm = -0.188
Psychometric function forLowAngle
Actual percentage
Just noticable difference
slope = -33.079
slower
faster
Psychometric function forHighAngle
Actual percentage
Just noticable difference
slope = -41.235
slower
faster

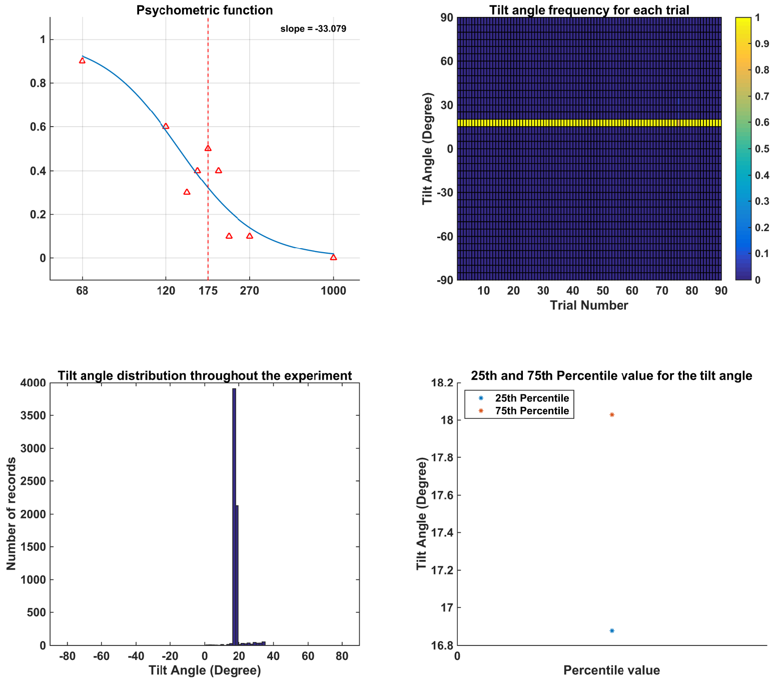
Psychometric function
slope = -33.079
Tilt angle frequency for each trial
Tilt Angle (Degree)
Trial Number
Tilt angle distribution throughout the experiment
Number of records
Tilt Angle (Degree)
25th and 75th Percentile value for the tilt angle
25th Percentile
75th Percentile
Tilt Angle (Degree)
Percentile value

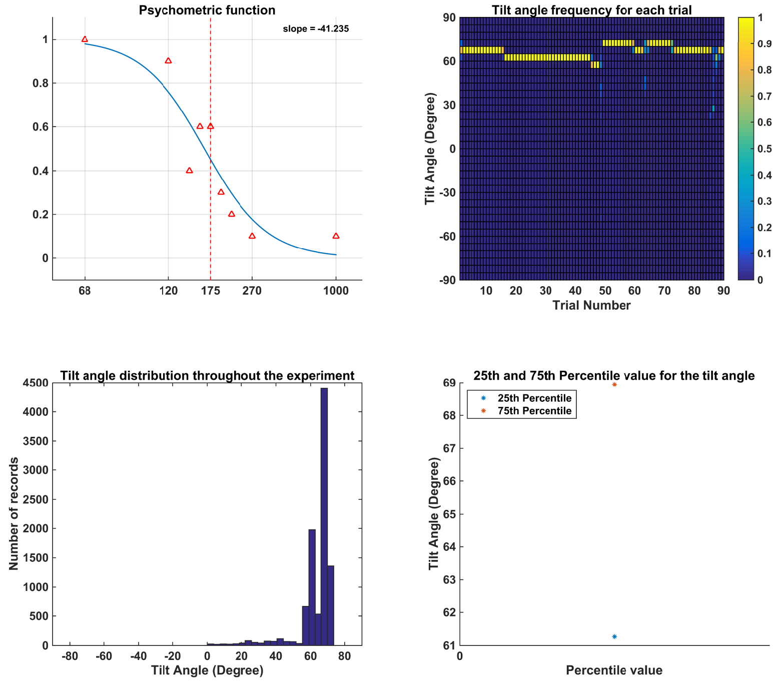
Psychometric function
slope = -41.235
Tilt angle frequency for each trial
Tilt Angle (Degree)
Trial Number
Tilt angle distribution throughout the experiment
Number of records
Tilt Angle (Degree)
25th and 75th Percentile value for the tilt angle
25th Percentile
75th Percentile
Tilt Angle (Degree)
Percentile value

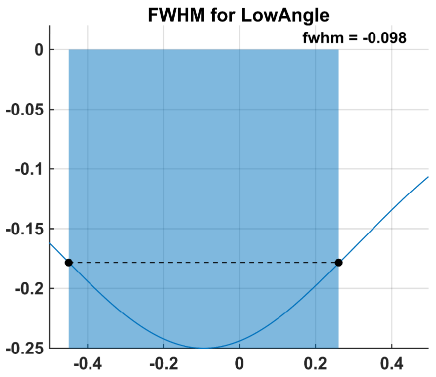
FWHM for LowAngle
fwhm = -0.098

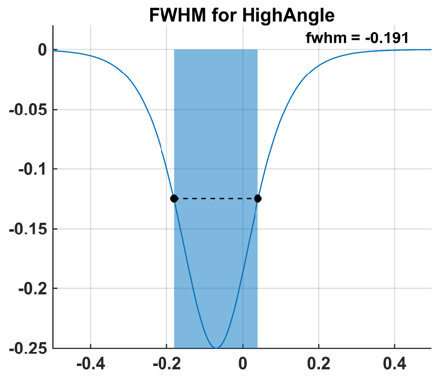
FWHM for HighAngle
fwhm = -0.191

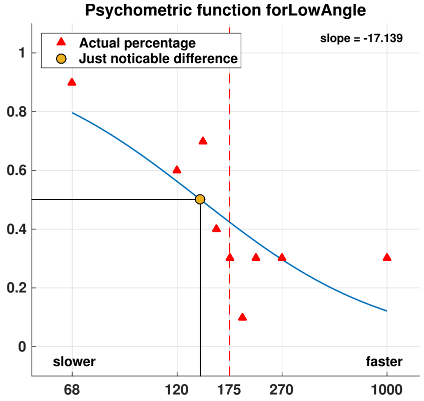
Psychometric function forLowAngle
Actual percentage
Just noticable difference
slope = -17.139
slower
faster

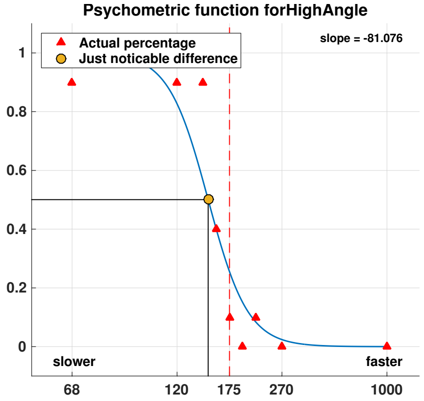
Psychometric function forHighAngle
Actual percentage
Just noticable difference
slope = -81.076
slower
faster

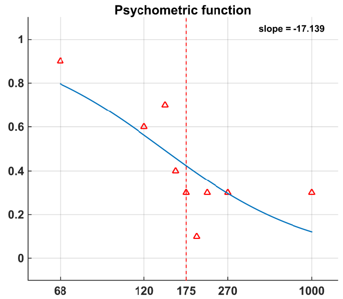
Psychometric function
slope = -17.139

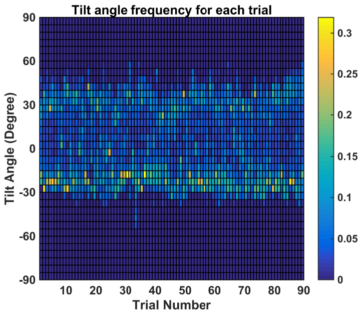
Tilt angle frequency for each trial
Tilt Angle (Degree)
Trial Number

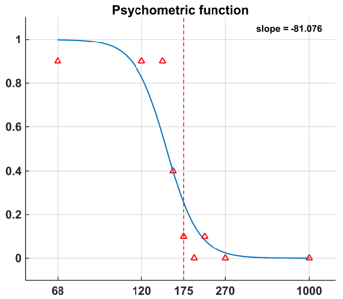
Psychometric function
slope = -81.076

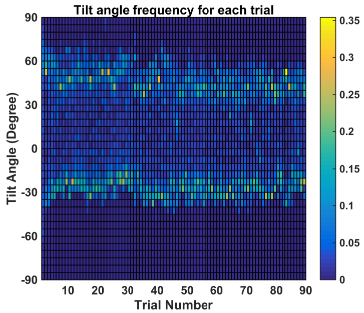
Tilt angle frequency for each trial
Tilt Angle (Degree)
Trial Number

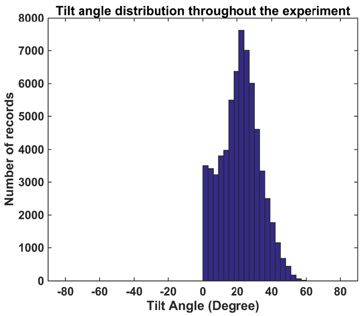
Tilt angle distribution throughout the experiment
Number of records
Tilt Angle (Degree)

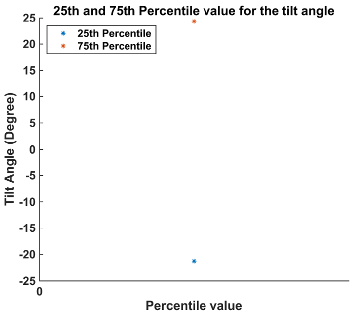
25th and 75th Percentile value for the tilt angle
25th Percentile
75th Percentile
Tilt Angle (Degree)
Percentile value

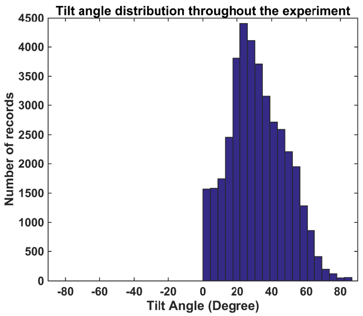
Tilt angle distribution throughout the experiment
Number of records
Tilt Angle (Degree)

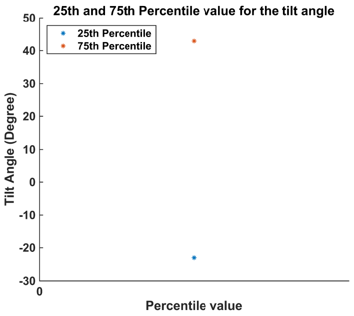
25th and 75th Percentile value for the tilt angle
25th Percentile
75th Percentile
Tilt Angle (Degree)
Percentile value

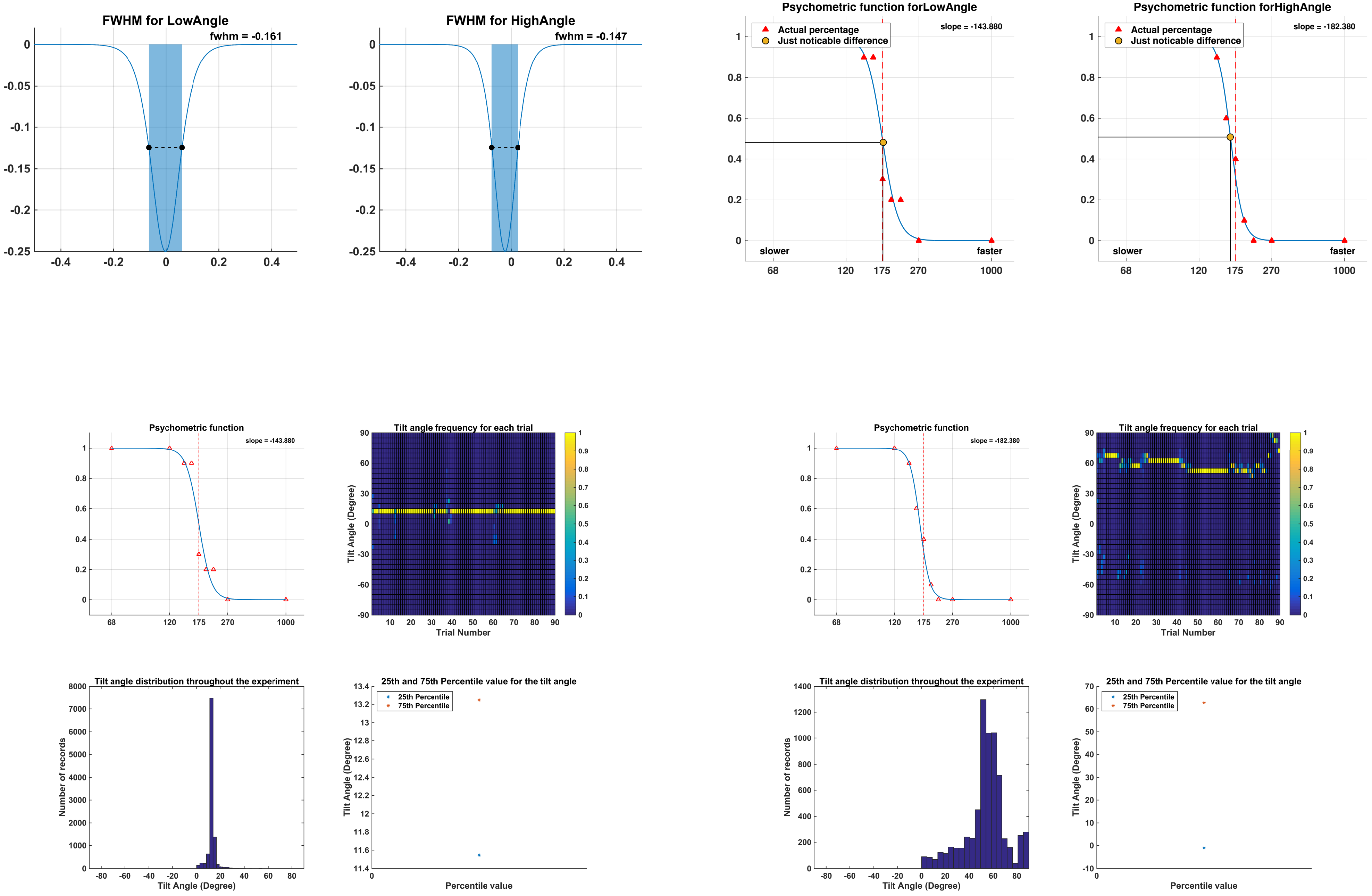

FWHM for LowAngle
fwhm = -0.161
FWHM for HighAngle
fwhm = -0.147
Psychometric function forLowAngle
slope = -143.880
Actual percentage
Just noticable difference
slower
faster
Psychometric function forHighAngle
slope = -182.380
Psychometric function
Tilt angle frequency for each trial
Tilt Angle (Degree)
Trial Number
Tilt angle distribution throughout the experiment
Number of records
25th and 75th Percentile value for the tilt angle
25th Percentile
75th Percentile
Percentile value

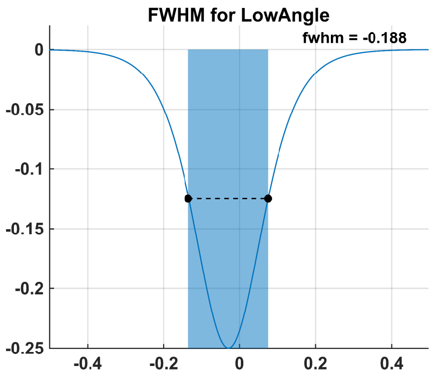
FWHM for LowAngle
fwhm = -0.188

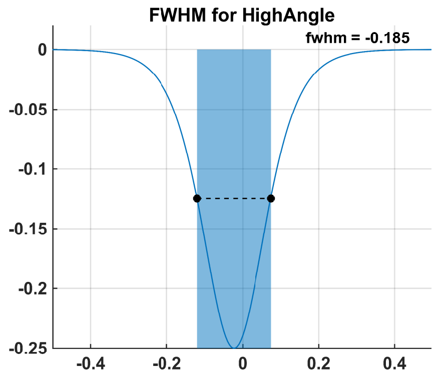
FWHM for HighAngle
fwhm = -0.185

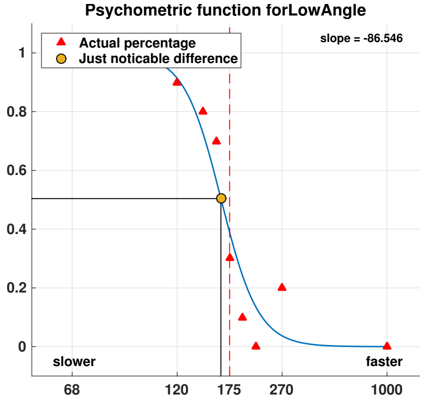
Psychometric function forLowAngle
Actual percentage
Just noticable difference
slope = -86.546
slower
faster

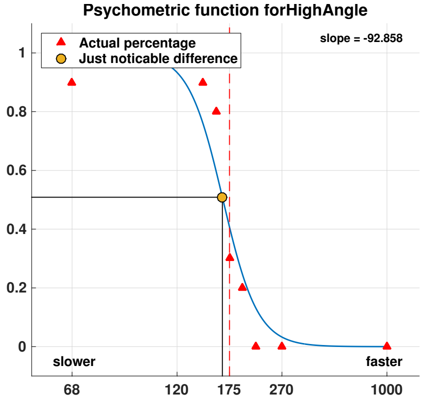
Psychometric function forHighAngle
Actual percentage
Just noticable difference
slope = -92.858
slower
faster

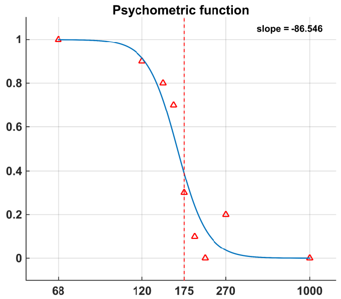
Psychometric function
slope = -86.546

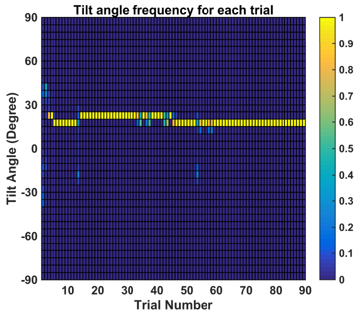
Tilt angle frequency for each trial
Tilt Angle (Degree)
Trial Number

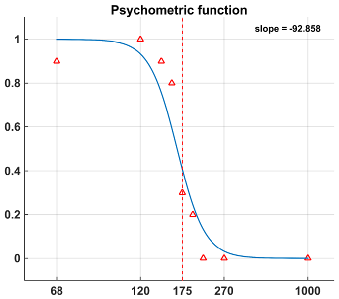
Psychometric function
slope = -92.858

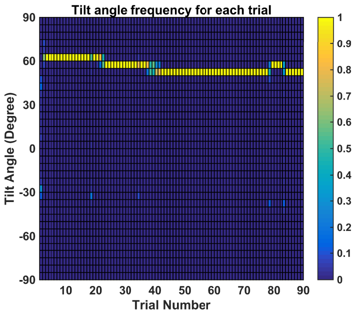
Tilt angle frequency for each trial
Tilt Angle (Degree)
Trial Number

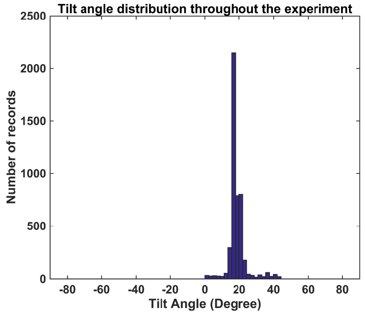
Tilt angle distribution throughout the experiment
Number of records
Tilt Angle (Degree)

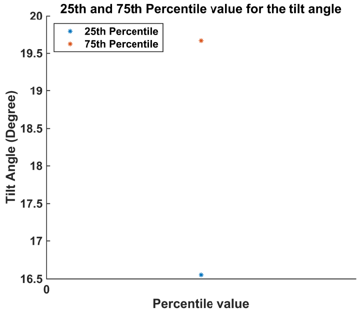
25th and 75th Percentile value for the tilt angle
25th Percentile
75th Percentile
Tilt Angle (Degree)
Percentile value

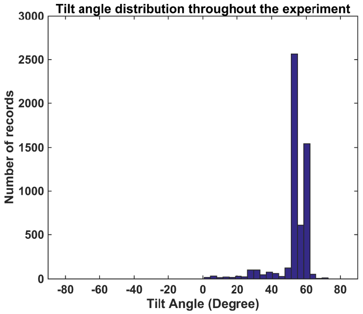
Tilt angle distribution throughout the experiment
Number of records
Tilt Angle (Degree)

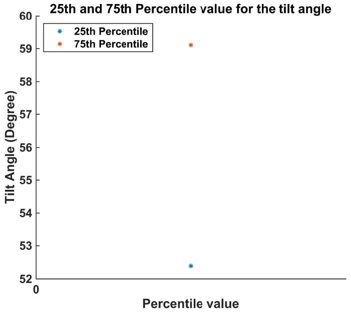
25th and 75th Percentile value for the tilt angle
25th Percentile
75th Percentile
Tilt Angle (Degree)
Percentile value

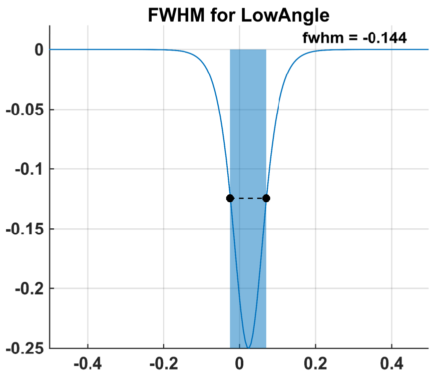
FWHM for LowAngle
fwhm = -0.144

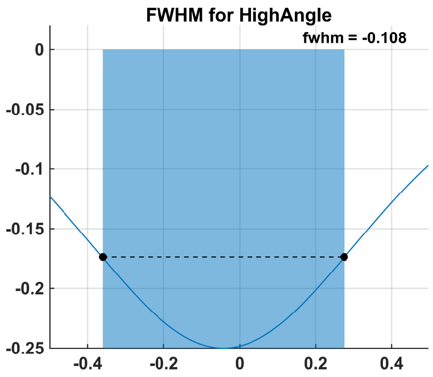
FWHM for HighAngle
fwhm = -0.108

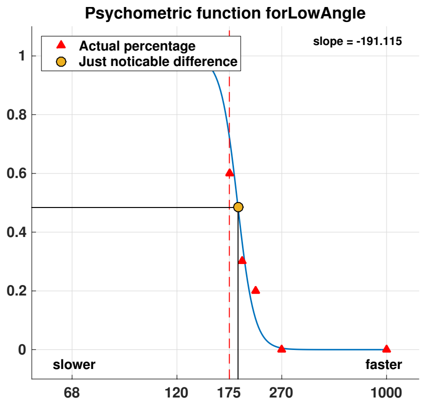
Psychometric function forLowAngle
Actual percentage
Just noticable difference
slope = -191.115
slower
faster

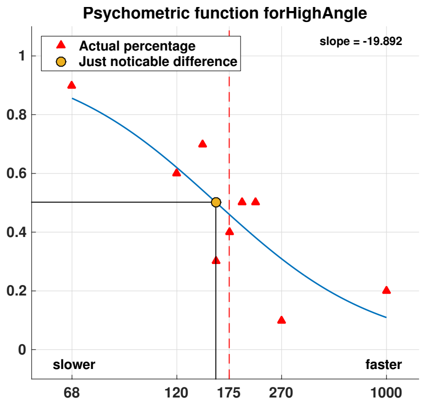
Psychometric function forHighAngle
Actual percentage
Just noticable difference
slope = -19.892
slower
faster

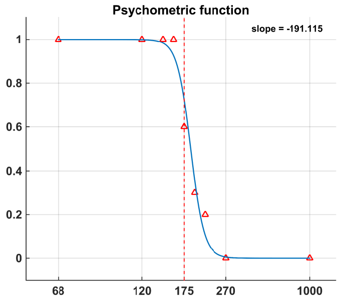
Psychometric function
slope = -191.115

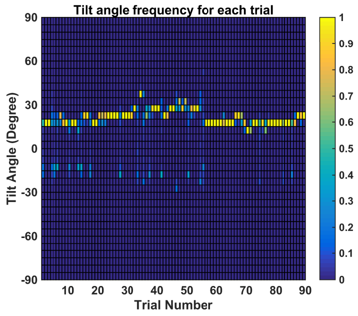
Tilt angle frequency for each trial
Tilt Angle (Degree)
Trial Number

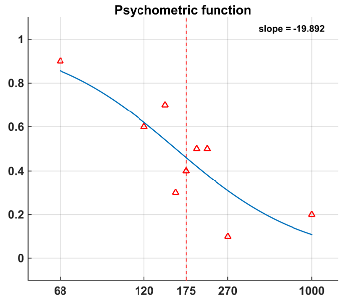
Psychometric function
slope = -19.892

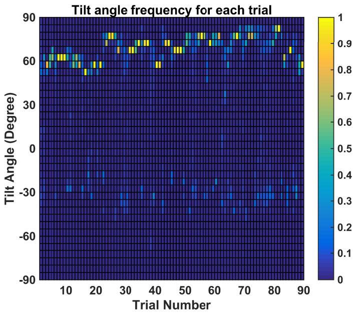
Tilt angle frequency for each trial
Tilt Angle (Degree)
Trial Number

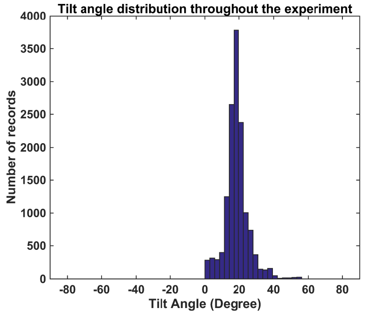
Tilt angle distribution throughout the experiment
Number of records
Tilt Angle (Degree)

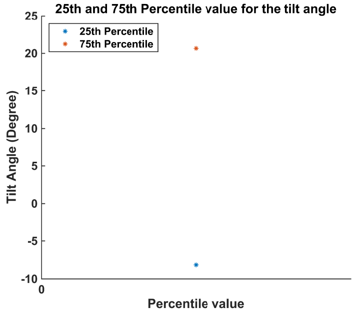
25th and 75th Percentile value for the tilt angle
25th Percentile
75th Percentile
Tilt Angle (Degree)
Percentile value

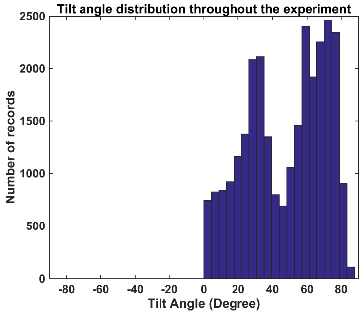
Tilt angle distribution throughout the experiment
Number of records
Tilt Angle (Degree)

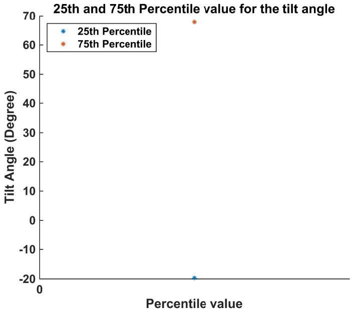
25th and 75th Percentile value for the tilt angle
25th Percentile
75th Percentile
Tilt Angle (Degree)
Percentile value

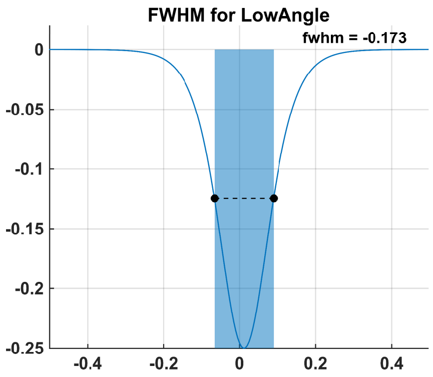
FWHM for LowAngle
fwhm = -0.173

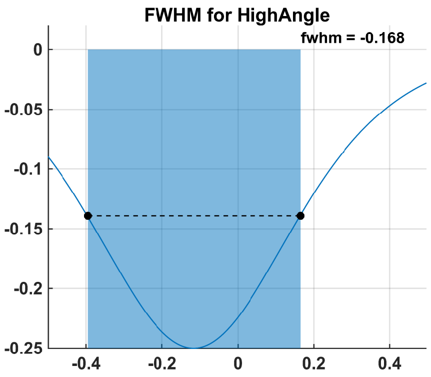
FWHM for HighAngle
fwhm = -0.168

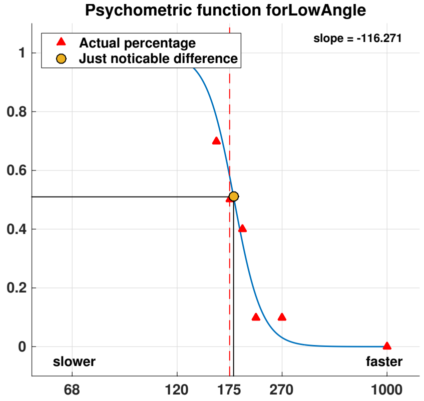
Psychometric function forLowAngle
Actual percentage
Just noticable difference
slope = -116.271
slower
faster

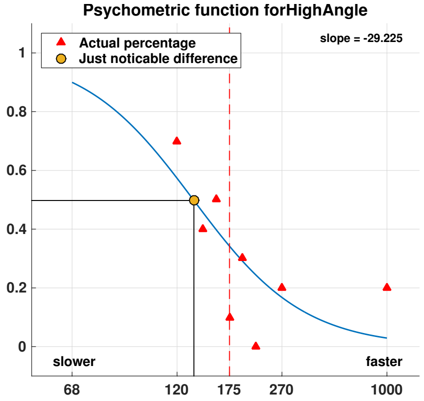
Psychometric function forHighAngle
Actual percentage
Just noticable difference
slope = -29.225
slower
faster

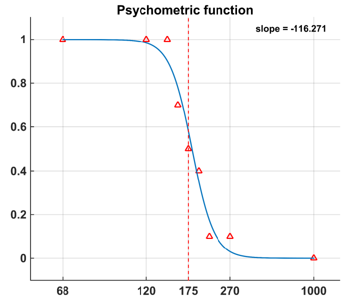
Psychometric function
slope = -116.271

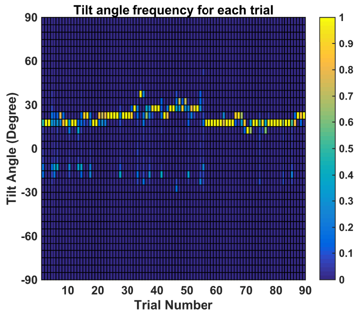
Tilt angle frequency for each trial
Tilt Angle (Degree)
Trial Number

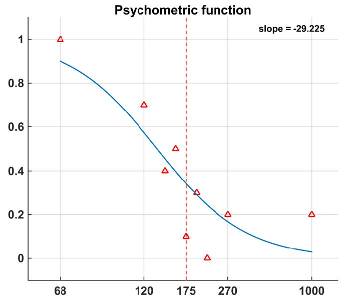
Psychometric function
slope = -29.225

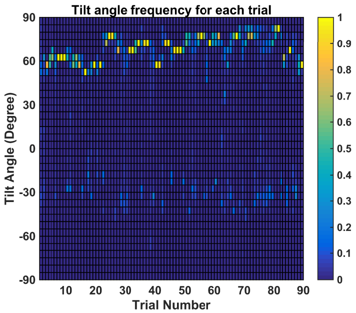
Tilt angle frequency for each trial
Tilt Angle (Degree)
Trial Number

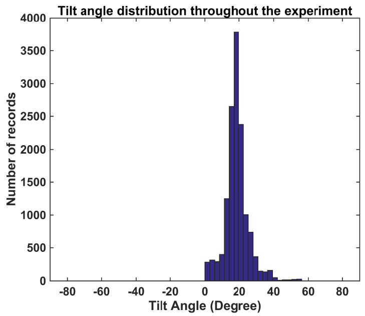
Tilt angle distribution throughout the experiment
Number of records
Tilt Angle (Degree)

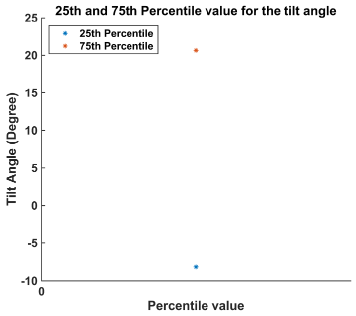
25th and 75th Percentile value for the tilt angle
25th Percentile
75th Percentile
Tilt Angle (Degree)
Percentile value

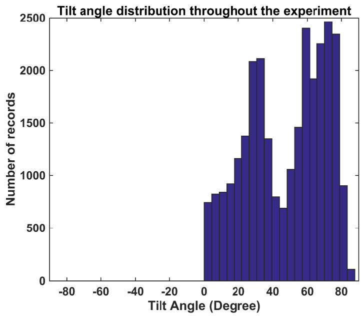
Tilt angle distribution throughout the experiment
Number of records
Tilt Angle (Degree)

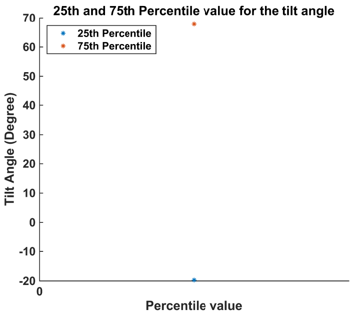
25th and 75th Percentile value for the tilt angle
25th Percentile
75th Percentile
Tilt Angle (Degree)
Percentile value

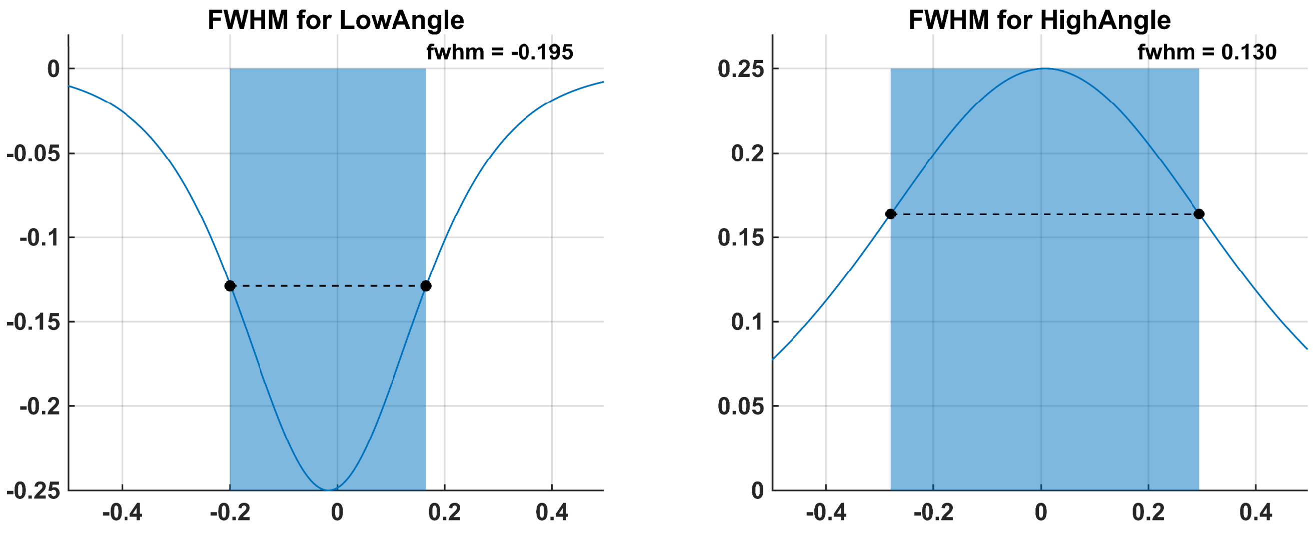

Individual participant data for Inverse group

Individual data for each participant from the Inverse group, namely the group which believes slower objects are heavier in both environments. This page is the group average. From page 36-56, each page corresponds to the data from each participant.

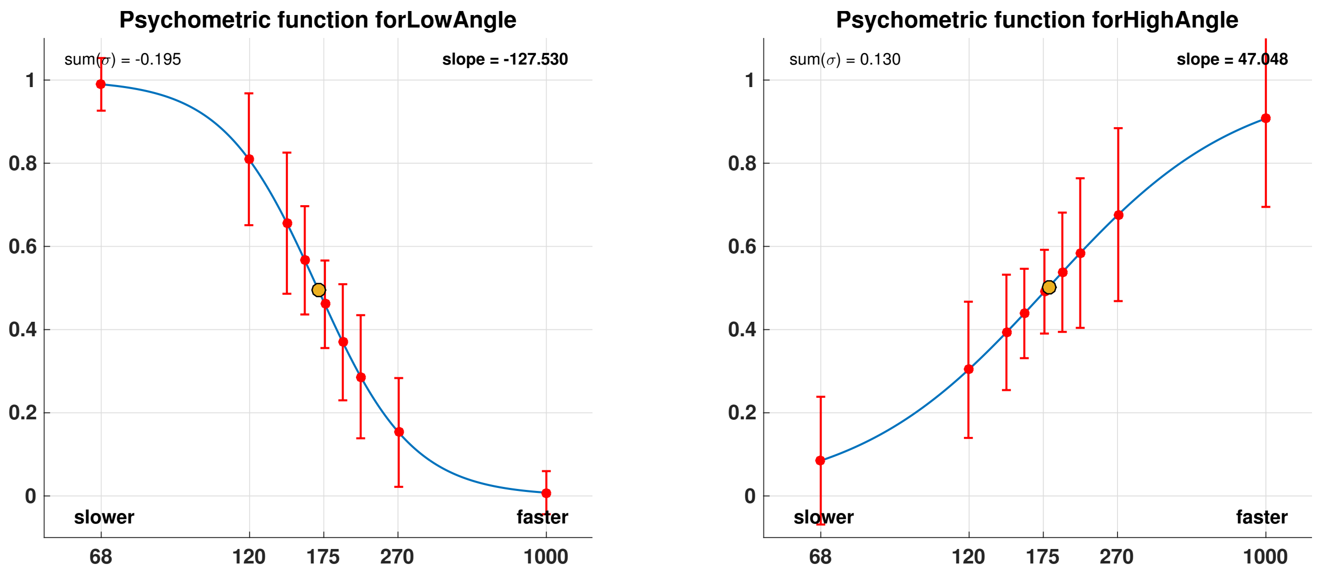

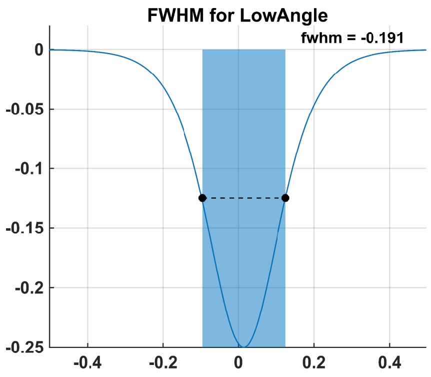
FWHM for LowAngle
fwhm = -0.191

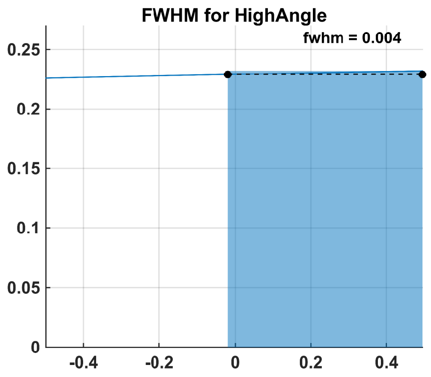
FWHM for HighAngle
fwhm = 0.004

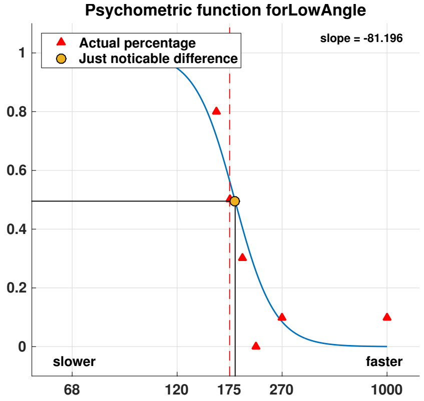
Psychometric function forLowAngle
Actual percentage
Just noticable difference
slope = -81.196
slower
faster

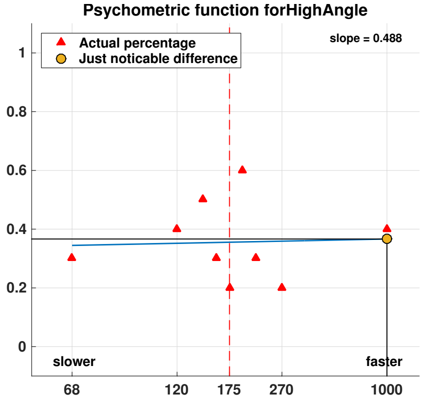
Psychometric function forHighAngle
Actual percentage
Just noticable difference
slope = 0.488
slower
faster

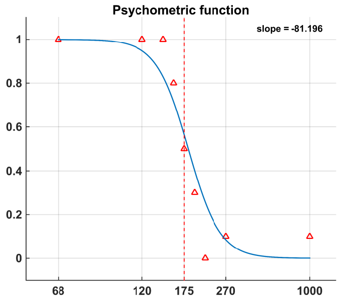
Psychometric function
slope = -81.196

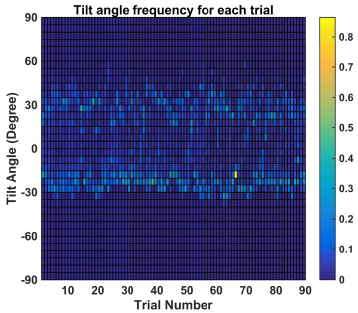
Tilt angle frequency for each trial
Tilt Angle (Degree)
Trial Number

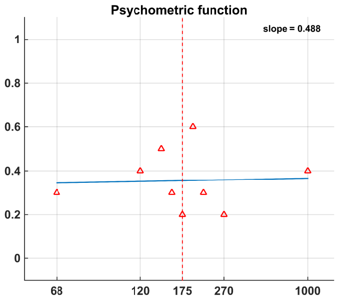
Psychometric function
slope = 0.488

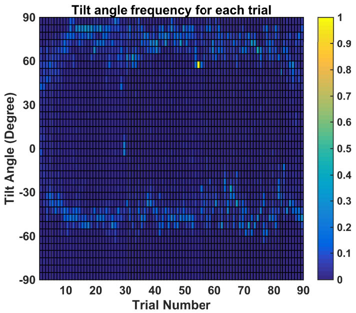
Tilt angle frequency for each trial
Tilt Angle (Degree)
Trial Number

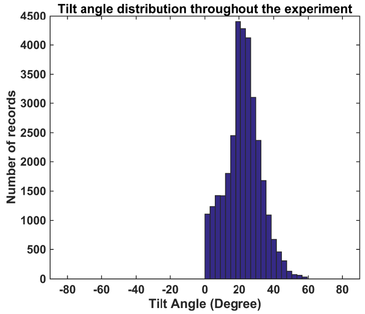
Tilt angle distribution throughout the experiment
Number of records
Tilt Angle (Degree)

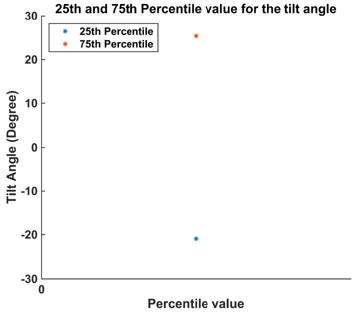
25th and 75th Percentile value for the tilt angle
25th Percentile
75th Percentile
Tilt Angle (Degree)
Percentile value

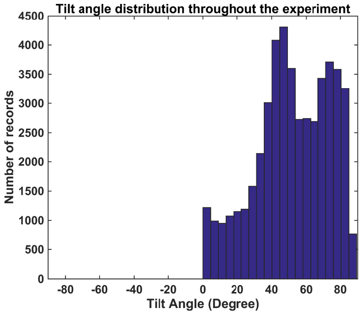
Tilt angle distribution throughout the experiment
Number of records
Tilt Angle (Degree)

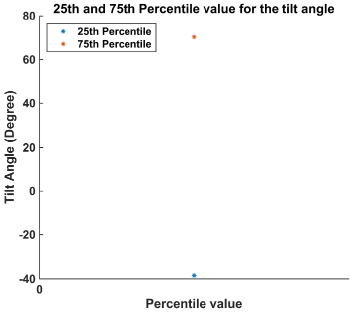
25th and 75th Percentile value for the tilt angle
25th Percentile
75th Percentile
Tilt Angle (Degree)
Percentile value

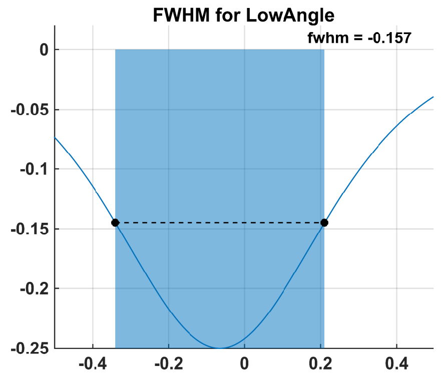
FWHM for LowAngle
fwhm = -0.157

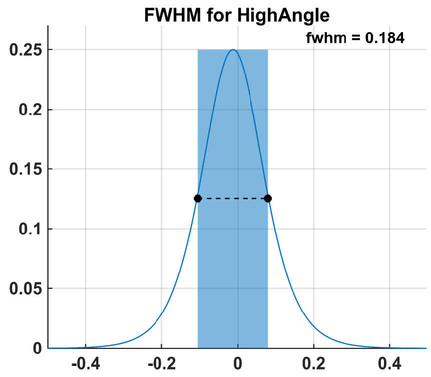
FWHM for HighAngle
fwhm = 0.184

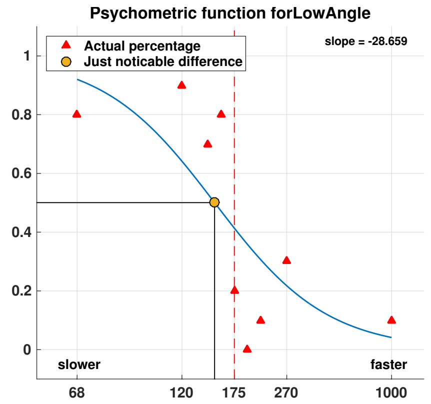
Psychometric function forLowAngle
Actual percentage
Just noticable difference
slope = -28.659
slower
faster

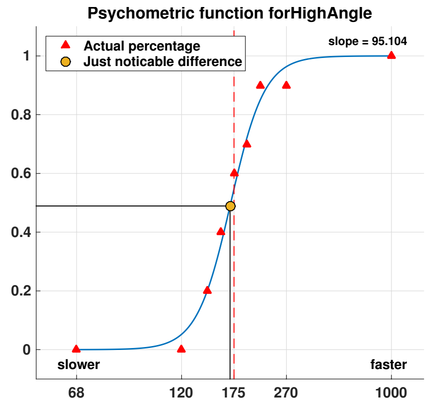
Psychometric function forHighAngle
Actual percentage
Just noticable difference
slope = 95.104
slower
faster

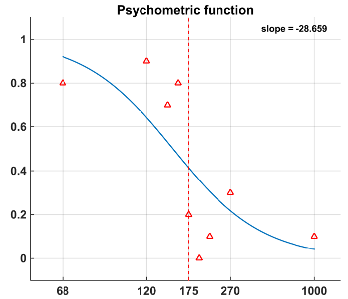
Psychometric function
slope = -28.659

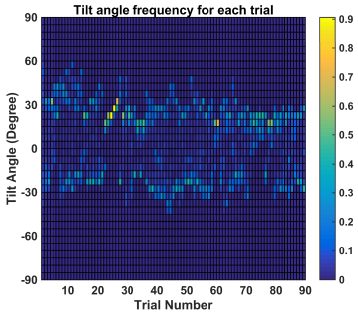
Tilt angle frequency for each trial
Tilt Angle (Degree)
Trial Number

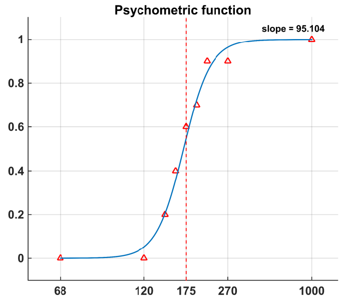
Psychometric function
slope = 95.104

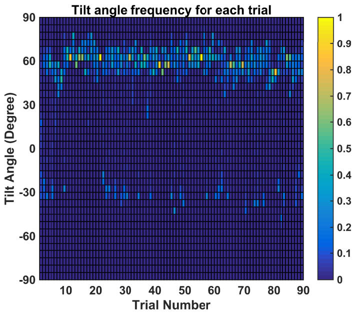
Tilt angle frequency for each trial
Tilt Angle (Degree)
Trial Number

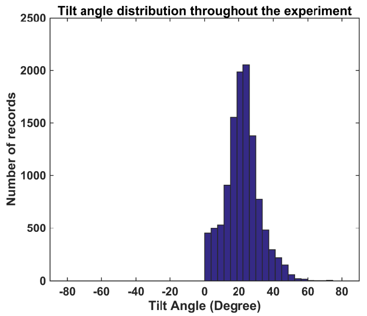
Tilt angle distribution throughout the experiment
Number of records
Tilt Angle (Degree)

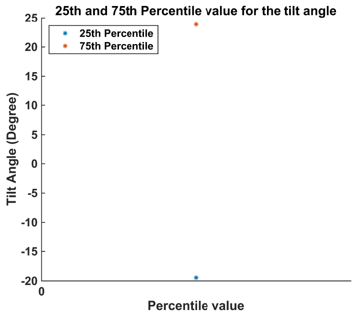
25th and 75th Percentile value for the tilt angle
25th Percentile
75th Percentile
Tilt Angle (Degree)
Percentile value

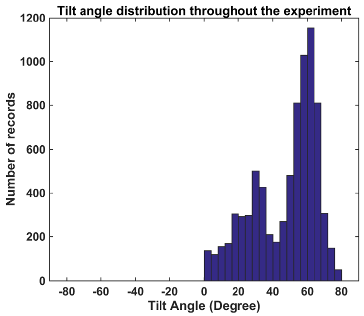
Tilt angle distribution throughout the experiment
Number of records
Tilt Angle (Degree)

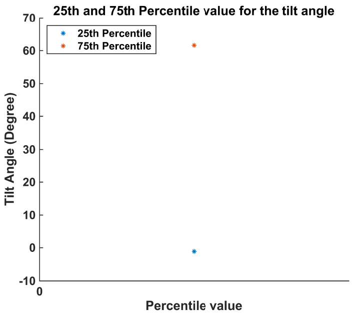
25th and 75th Percentile value for the tilt angle
25th Percentile
75th Percentile
Tilt Angle (Degree)
Percentile value

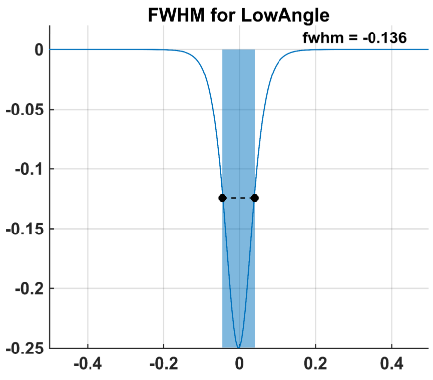
FWHM for LowAngle
fwhm = -0.136

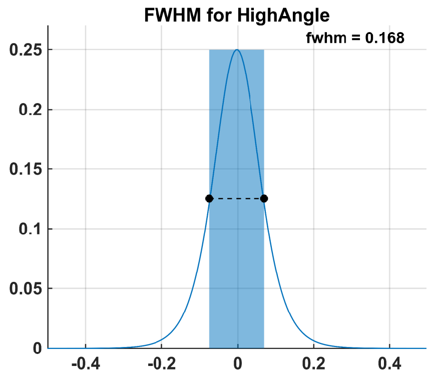
FWHM for HighAngle
fwhm = 0.168

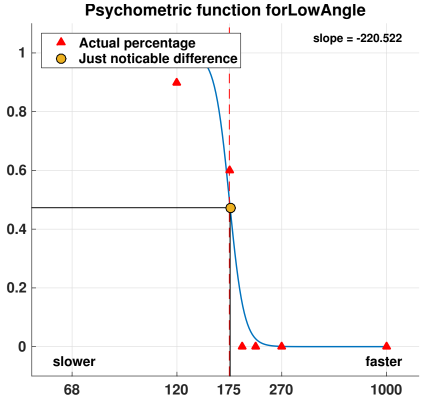
Psychometric function forLowAngle
Actual percentage
Just noticable difference
slope = -220.522
slower
faster

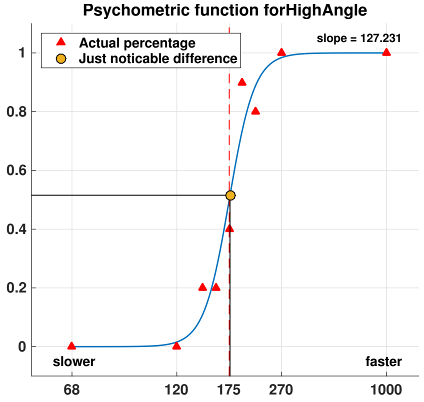
Psychometric function forHighAngle
Actual percentage
Just noticable difference
slope = 127.231
slower
faster

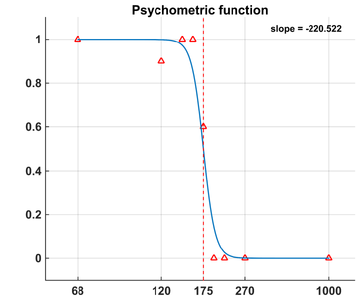
Psychometric function
slope = -220.522

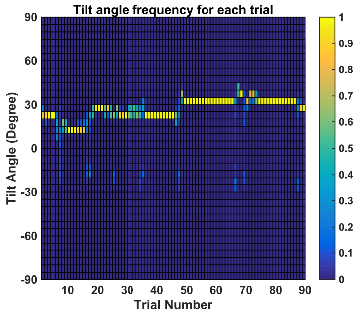
Tilt angle frequency for each trial
Tilt Angle (Degree)
Trial Number

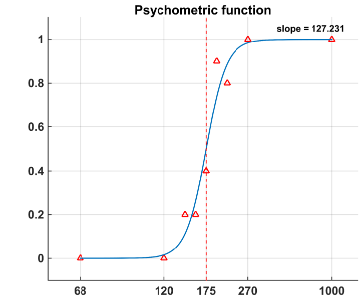
Psychometric function
slope = 127.231

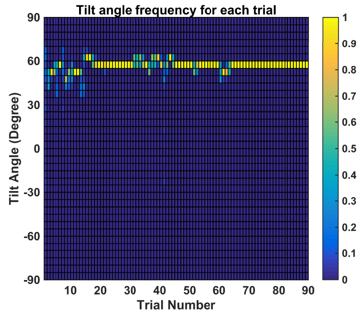
Tilt angle frequency for each trial
Tilt Angle (Degree)
Trial Number

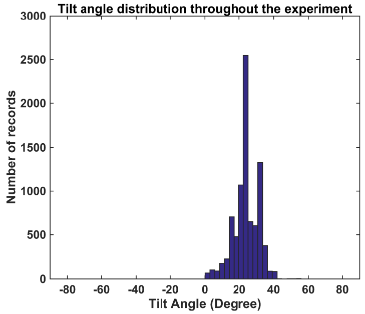
Tilt angle distribution throughout the experiment
Number of records
Tilt Angle (Degree)

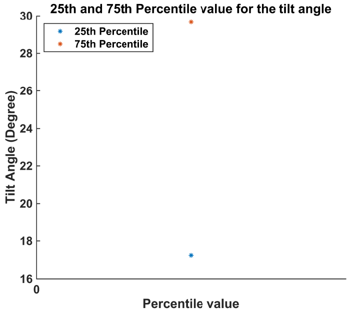
25th and 75th Percentile value for the tilt angle
25th Percentile
75th Percentile
Tilt Angle (Degree)
Percentile value

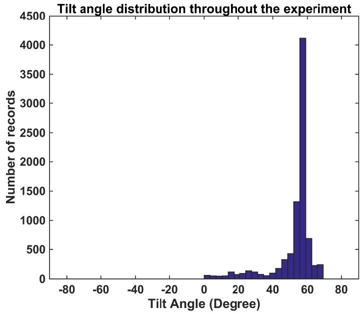
Tilt angle distribution throughout the experiment
Number of records
Tilt Angle (Degree)

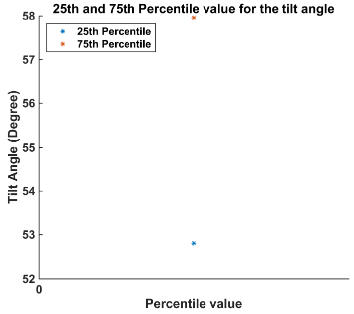
25th and 75th Percentile value for the tilt angle
25th Percentile
75th Percentile
Tilt Angle (Degree)
Percentile value

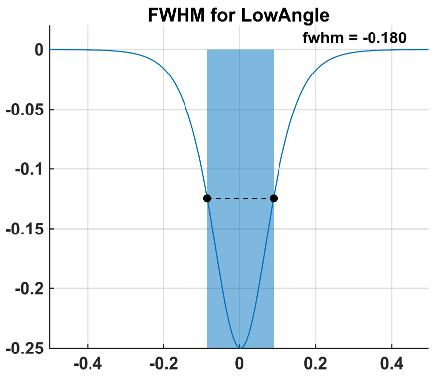
FWHM for LowAngle
fwhm = -0.180

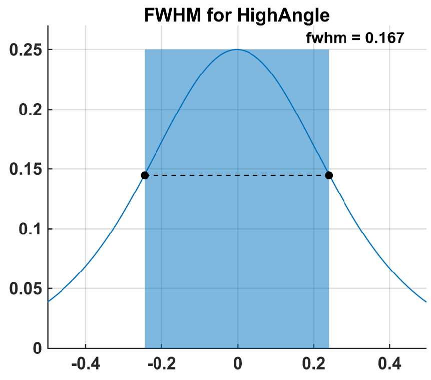
FWHM for HighAngle
fwhm = 0.167

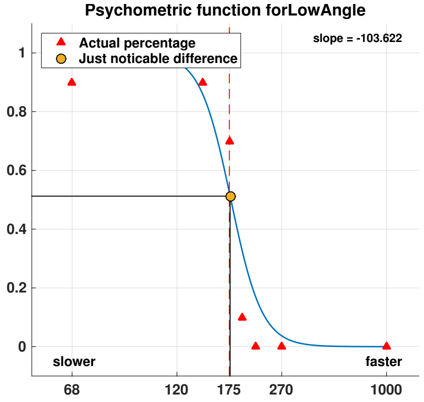
Psychometric function forLowAngle
Actual percentage
Just noticable difference
slope = -103.622
slower
faster

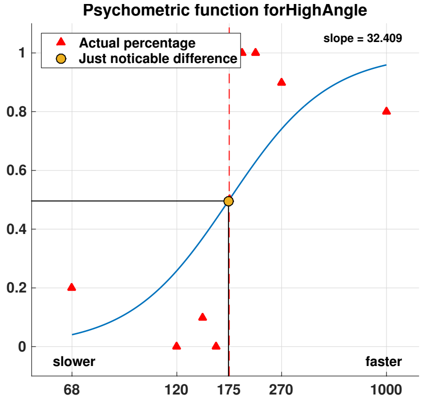
Psychometric function forHighAngle
Actual percentage
Just noticable difference
slope = 32.409
slower
faster

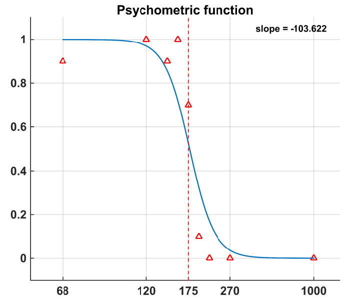
Psychometric function
slope = -103.622

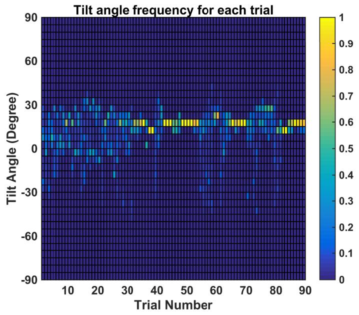
Tilt angle frequency for each trial
Tilt Angle (Degree)
Trial Number

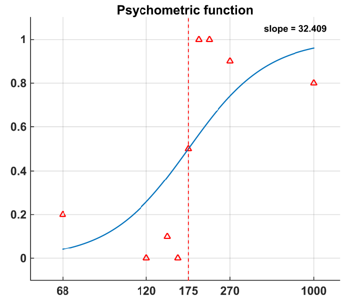
Psychometric function
slope = 32.409

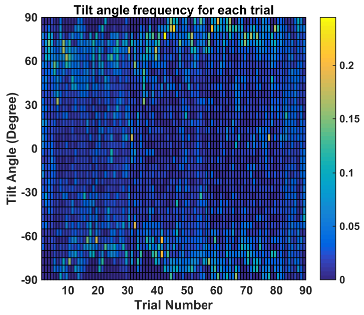
Tilt angle frequency for each trial
Tilt Angle (Degree)
Trial Number

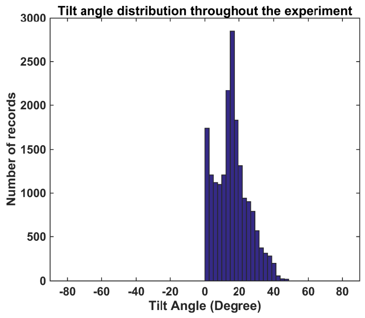
Tilt angle distribution throughout the experiment
Number of records
Tilt Angle (Degree)

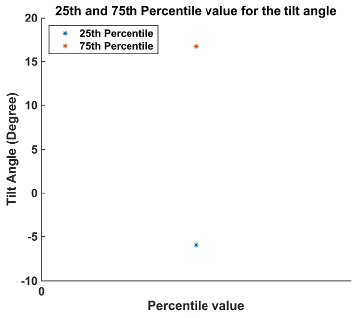
25th and 75th Percentile value for the tilt angle
25th Percentile
75th Percentile
Tilt Angle (Degree)
Percentile value

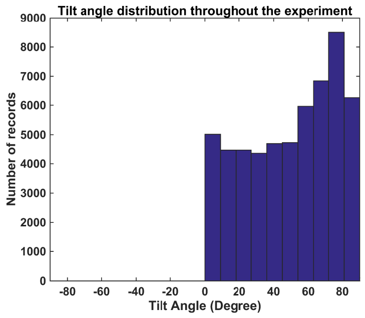
Tilt angle distribution throughout the experiment
Number of records
Tilt Angle (Degree)

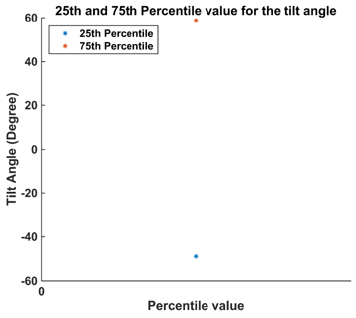
25th and 75th Percentile value for the tilt angle
25th Percentile
75th Percentile
Tilt Angle (Degree)
Percentile value

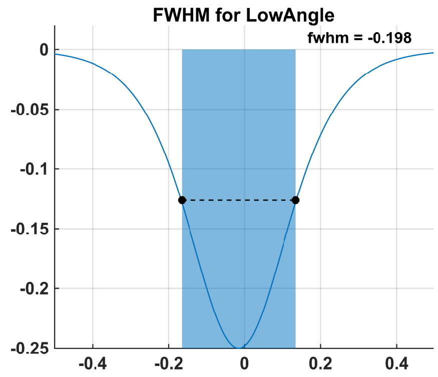
FWHM for LowAngle
fwhm = -0.198

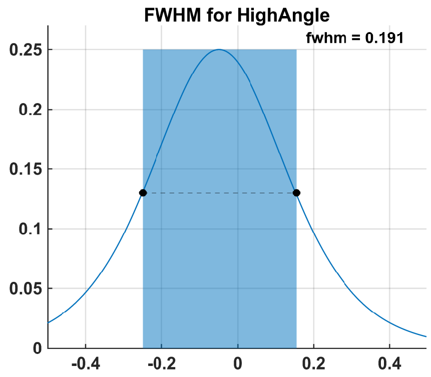
FWHM for HighAngle
fwhm = 0.191

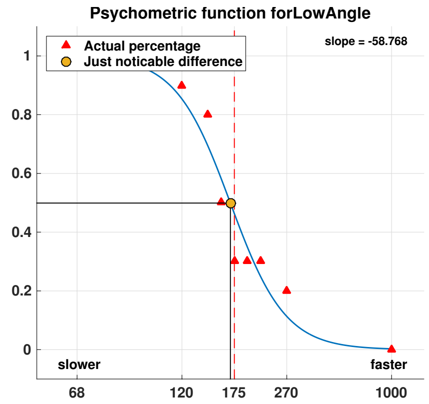
Psychometric function forLowAngle
Actual percentage
Just noticable difference
slope = -58.768
slower
faster

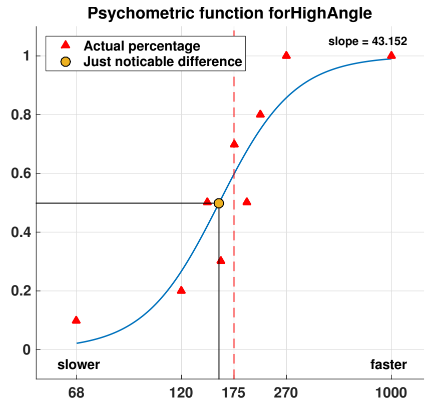
Psychometric function forHighAngle
Actual percentage
Just noticable difference
slope = 43.152
slower
faster

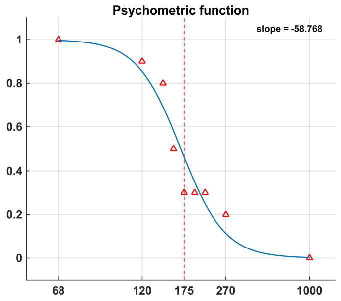
Psychometric function
slope = -58.768

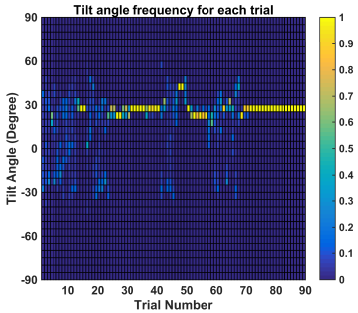
Tilt angle frequency for each trial
Tilt Angle (Degree)
Trial Number

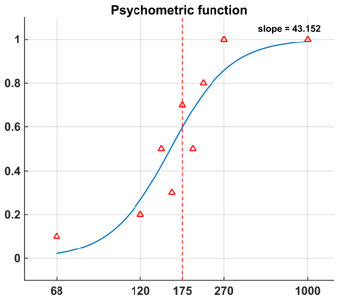
Psychometric function
slope = 43.152

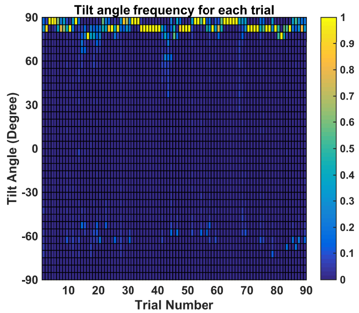
Tilt angle frequency for each trial
Tilt Angle (Degree)
Trial Number

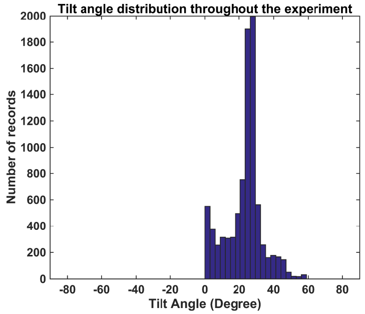
Tilt angle distribution throughout the experiment
Number of records
Tilt Angle (Degree)

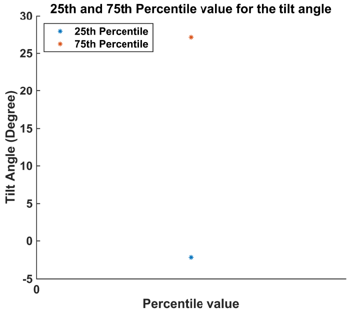
25th and 75th Percentile value for the tilt angle
25th Percentile
75th Percentile
Tilt Angle (Degree)
Percentile value

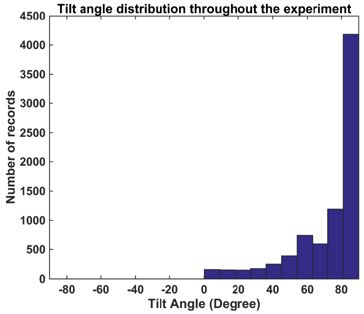
Tilt angle distribution throughout the experiment
Number of records
Tilt Angle (Degree)

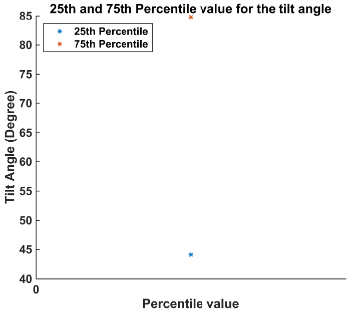
25th and 75th Percentile value for the tilt angle
25th Percentile
75th Percentile
Tilt Angle (Degree)
Percentile value

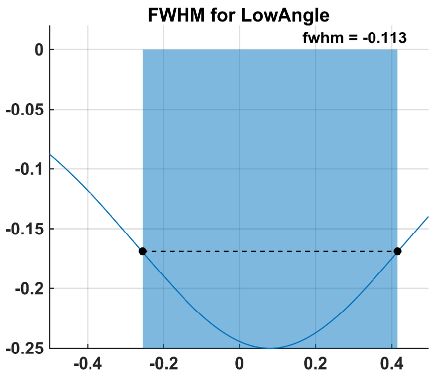
FWHM for LowAngle
fwhm = -0.113
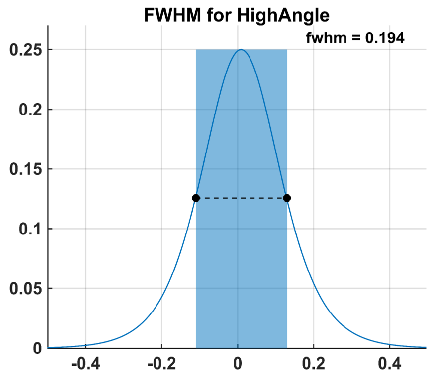
FWHM for HighAngle
fwhm = 0.194
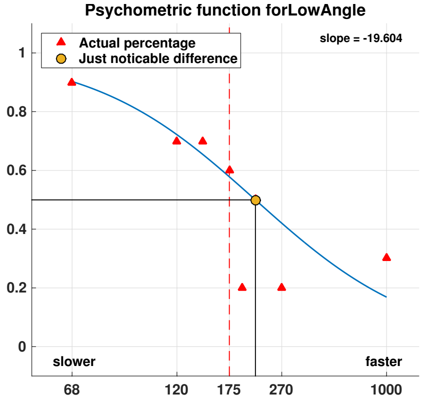
Psychometric function forLowAngle
Actual percentage
Just noticable difference
slope = -19.604
slower
faster
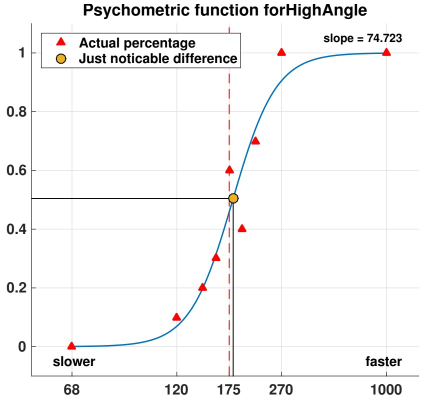
Psychometric function forHighAngle
Actual percentage
Just noticable difference
slope = 74.723
slower
faster
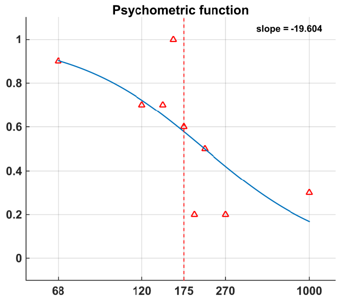
Psychometric function
slope = -19.604
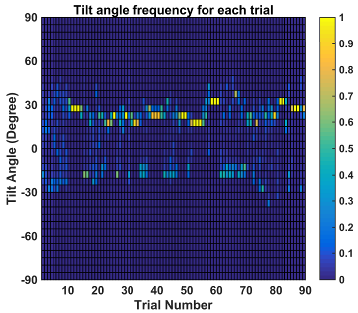
Tilt angle frequency for each trial
Tilt Angle (Degree)
Trial Number
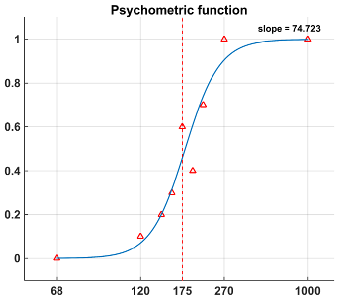
Psychometric function
slope = 74.723
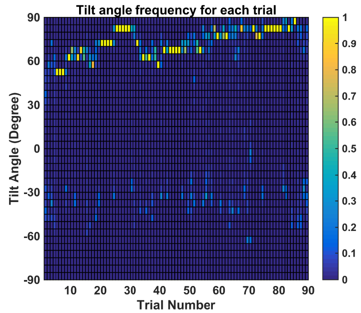
Tilt angle frequency for each trial
Tilt Angle (Degree)
Trial Number
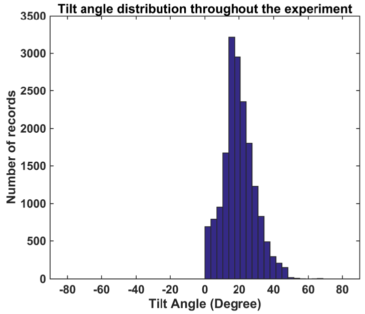
Tilt angle distribution throughout the experiment
Number of records
Tilt Angle (Degree)
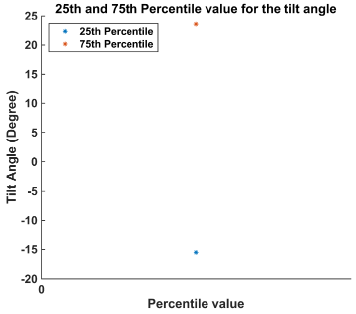
25th and 75th Percentile value for the tilt angle
25th Percentile
75th Percentile
Tilt Angle (Degree)
Percentile value
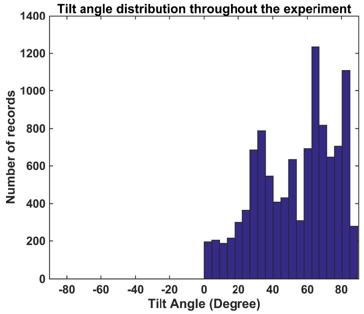
Tilt angle distribution throughout the experiment
Number of records
Tilt Angle (Degree)
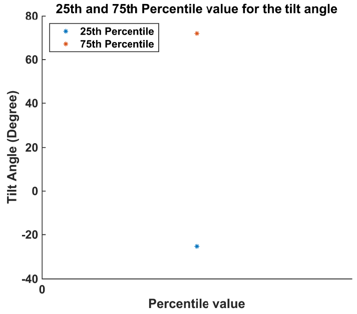
25th and 75th Percentile value for the tilt angle
25th Percentile
75th Percentile
Tilt Angle (Degree)
Percentile value

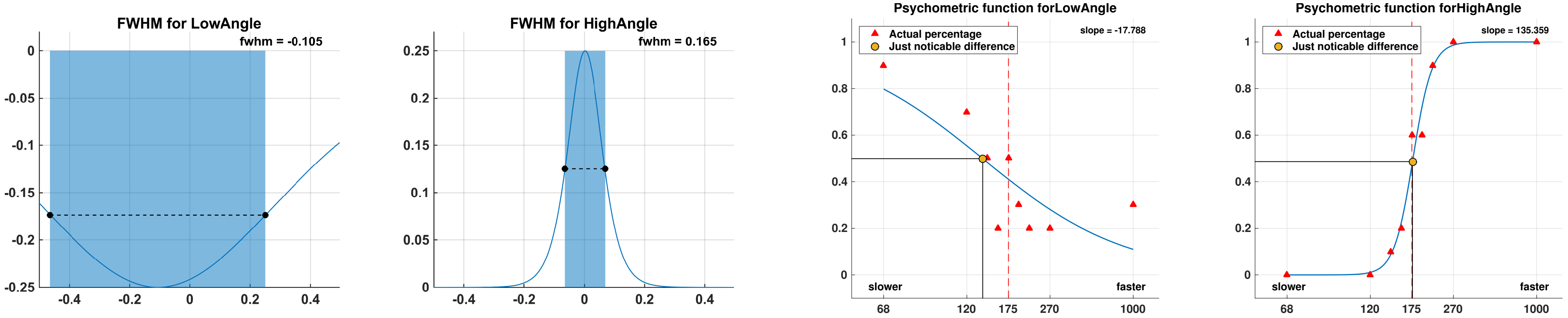
FWHM for LowAngle
fwhm = -0.105
FWHM for HighAngle
fwhm = 0.165
Psychometric function forLowAngle
Actual percentage
Just noticable difference
slope = -17.788
slower
faster
Psychometric function forHighAngle
Actual percentage
Just noticable difference
slope = 135.359
slower
faster

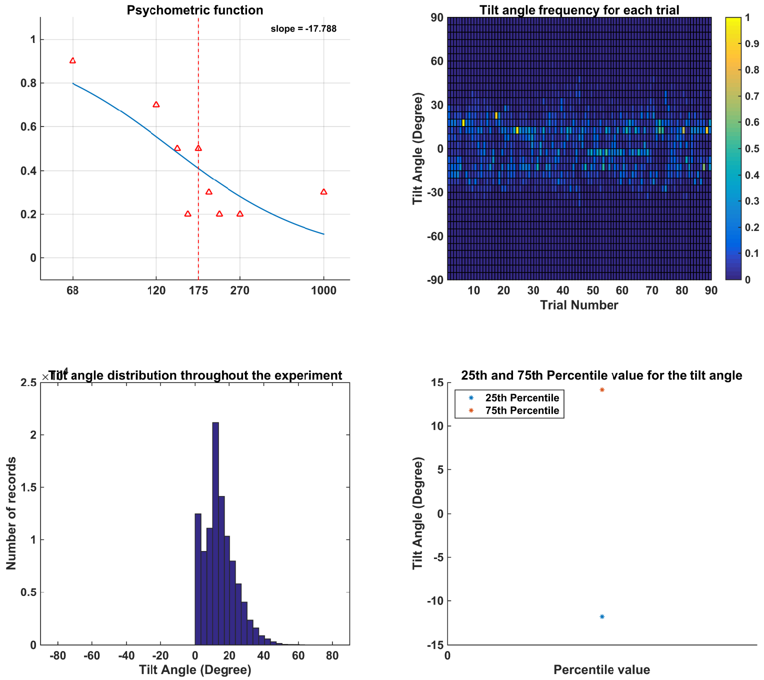
Psychometric function
slope = -17.788
Tilt angle frequency for each trial
Tilt Angle (Degree)
Trial Number
Tilt angle distribution throughout the experiment
Number of records
Tilt Angle (Degree)
25th and 75th Percentile value for the tilt angle
25th Percentile
75th Percentile
Tilt Angle (Degree)
Percentile value

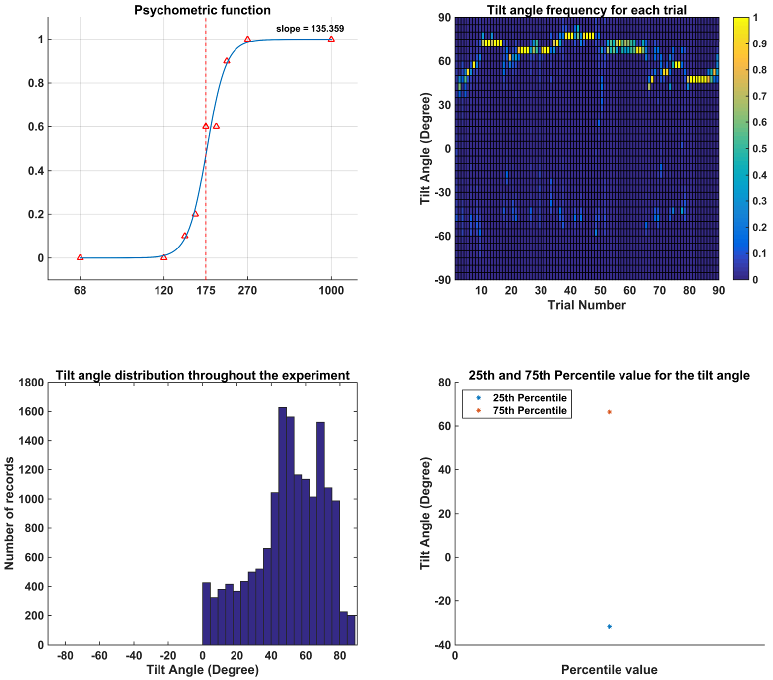
Psychometric function
slope = 135.359
Tilt angle frequency for each trial
Tilt Angle (Degree)
Trial Number
Tilt angle distribution throughout the experiment
Number of records
Tilt Angle (Degree)
25th and 75th Percentile value for the tilt angle
25th Percentile
75th Percentile
Tilt Angle (Degree)
Percentile value

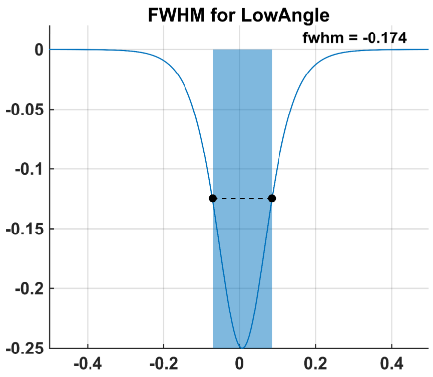
FWHM for LowAngle
fwhm = -0.174

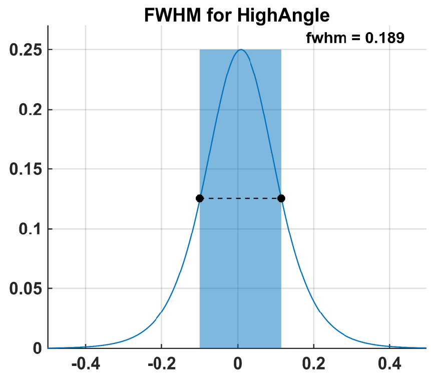
FWHM for HighAngle
fwhm = 0.189

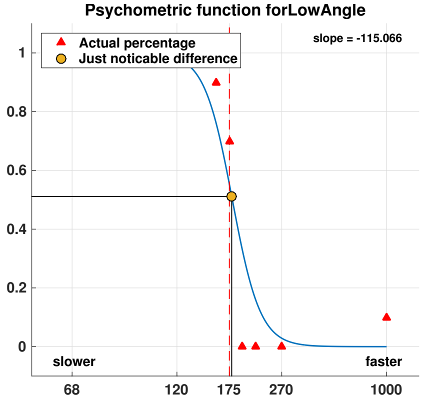
Psychometric function forLowAngle
Actual percentage
Just noticable difference
slope = -115.066
slower
faster

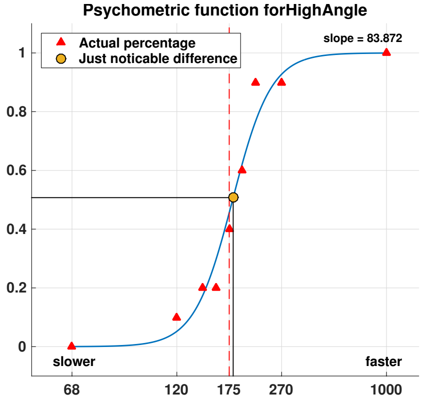
Psychometric function forHighAngle
Actual percentage
Just noticable difference
slope = 83.872
slower
faster

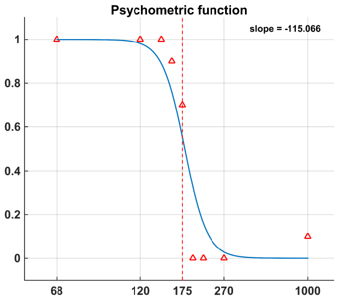
Psychometric function
slope = -115.066

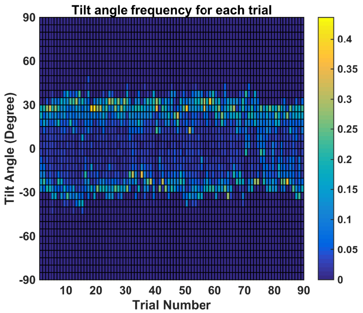
Tilt angle frequency for each trial
Tilt Angle (Degree)
Trial Number

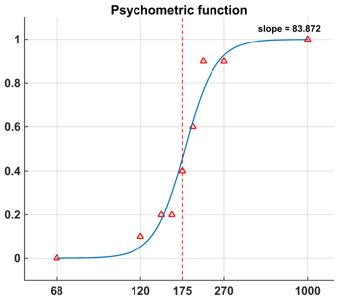
Psychometric function
slope = 83.872

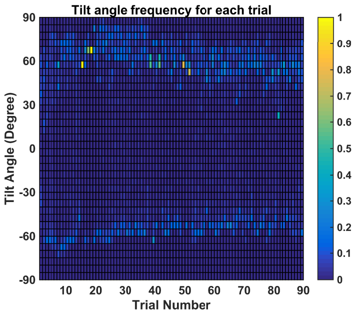
Tilt angle frequency for each trial
Tilt Angle (Degree)
Trial Number

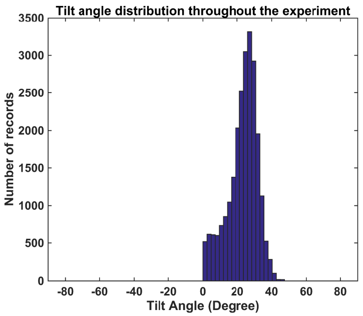
Tilt angle distribution throughout the experiment
Number of records
Tilt Angle (Degree)

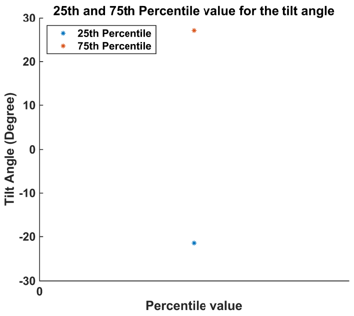
25th and 75th Percentile value for the tilt angle
25th Percentile
75th Percentile
Tilt Angle (Degree)
Percentile value

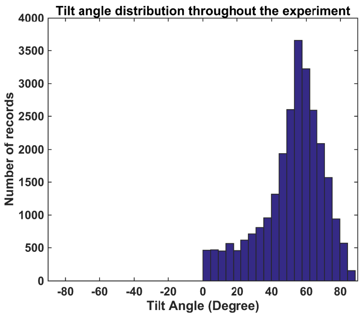
Tilt angle distribution throughout the experiment
Number of records
Tilt Angle (Degree)

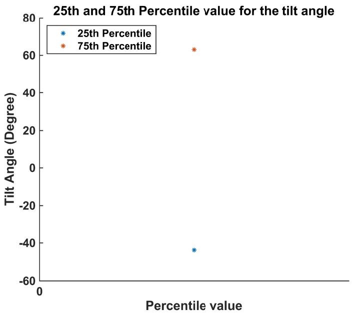
25th and 75th Percentile value for the tilt angle
25th Percentile
75th Percentile
Tilt Angle (Degree)
Percentile value

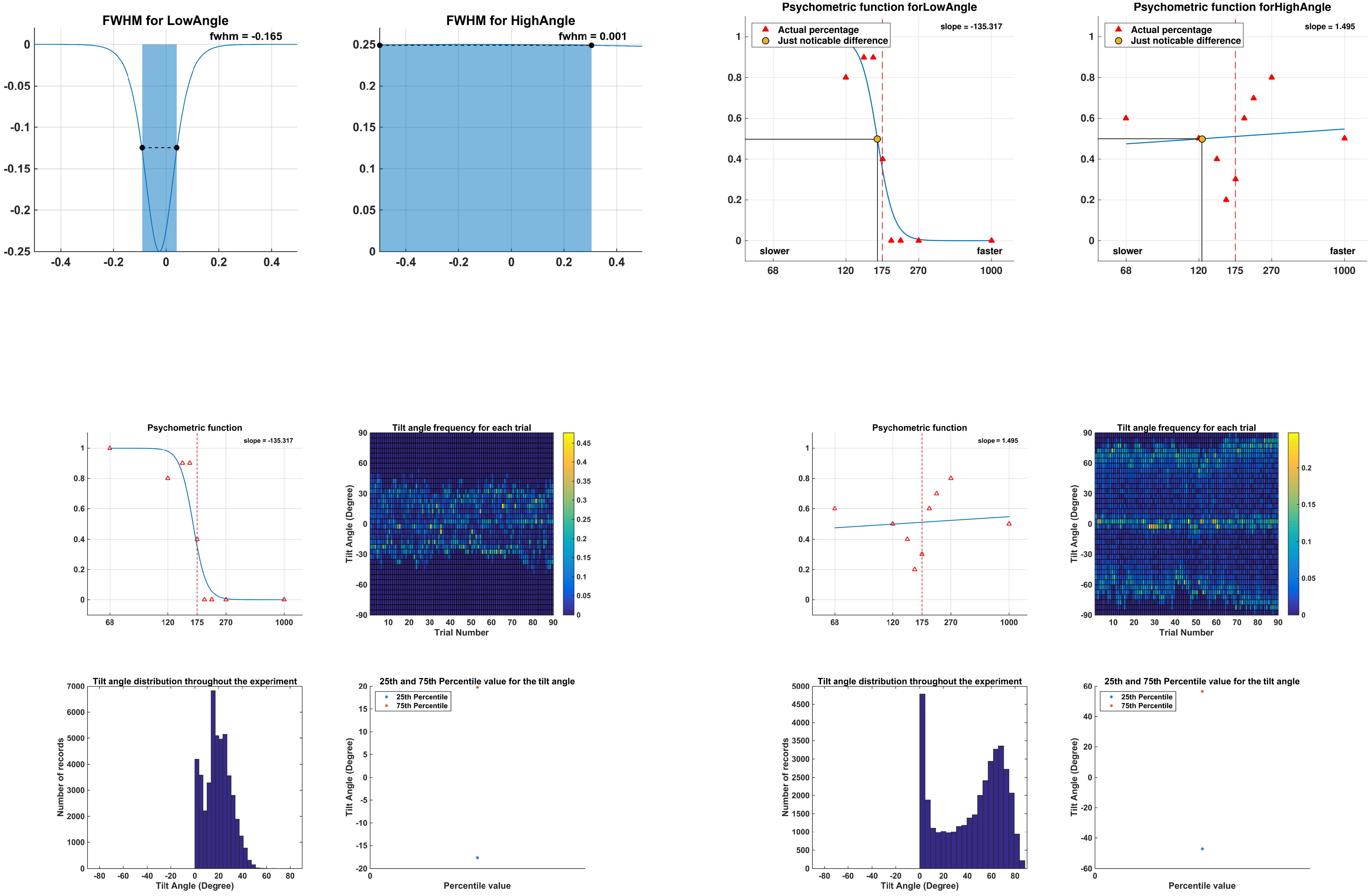

FWHM for LowAngle
fwhm = -0.165
FWHM for HighAngle
fwhm = 0.001
Psychometric function forLowAngle
Actual percentage
Just noticable difference
slope = -135.317
slower
faster
Psychometric function forHighAngle
Actual percentage
Just noticable difference
slope = 1.495
slower
faster
Psychometric function
slope = -135.317
Tilt angle frequency for each trial
Tilt Angle (Degree)
Trial Number
Psychometric function
slope = 1.495
Tilt angle frequency for each trial
Tilt Angle (Degree)
Trial Number
Tilt angle distribution throughout the experiment
Number of records
Tilt Angle (Degree)
25th and 75th Percentile value for the tilt angle
25th Percentile
75th Percentile
Tilt Angle (Degree)
Percentile value
Tilt angle distribution throughout the experiment
Number of records
Tilt Angle (Degree)
25th and 75th Percentile value for the tilt angle
25th Percentile
75th Percentile
Tilt Angle (Degree)
Percentile value

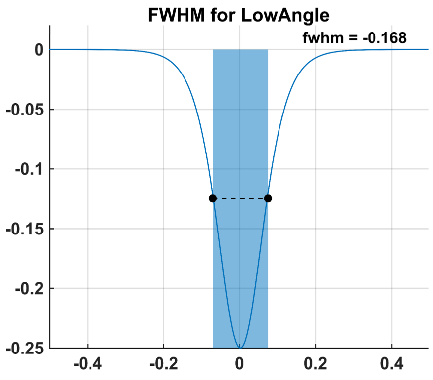
FWHM for LowAngle
fwhm = -0.168

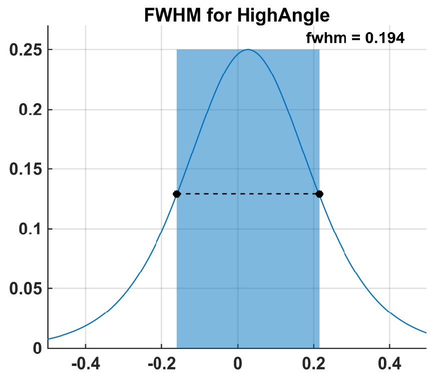
FWHM for HighAngle
fwhm = 0.194

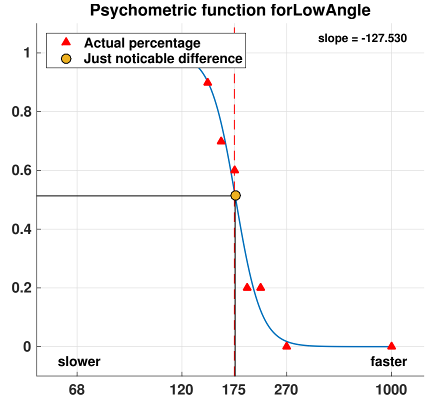
Psychometric function forLowAngle
Actual percentage
Just noticable difference
slope = -127.530
slower
faster

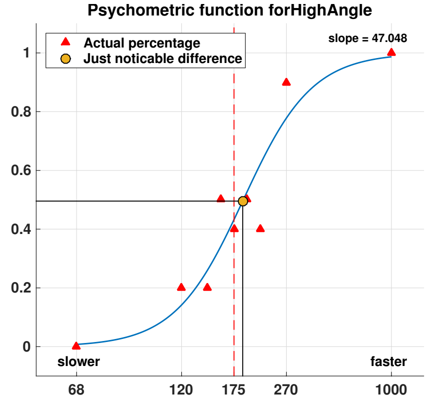
Psychometric function forHighAngle
Actual percentage
Just noticable difference
slope = 47.048
slower
faster

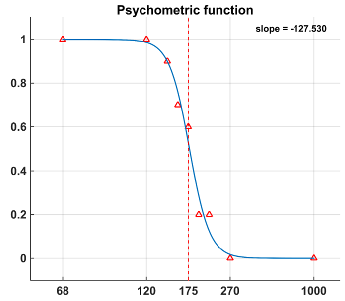
Psychometric function
slope = -127.530

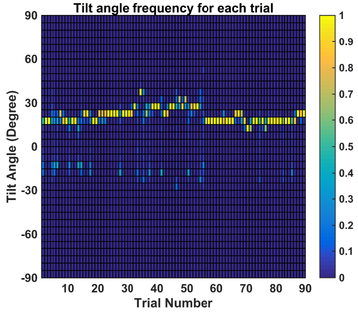
Tilt angle frequency for each trial
Tilt Angle (Degree)
Trial Number

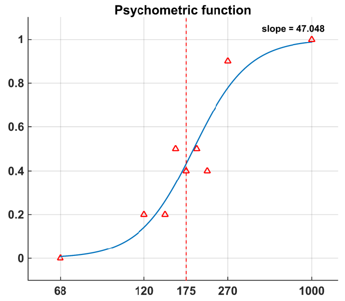
Psychometric function
slope = 47.048

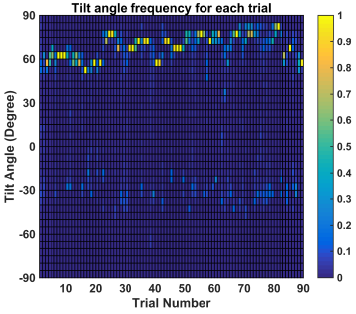
Tilt angle frequency for each trial
Tilt Angle (Degree)
Trial Number

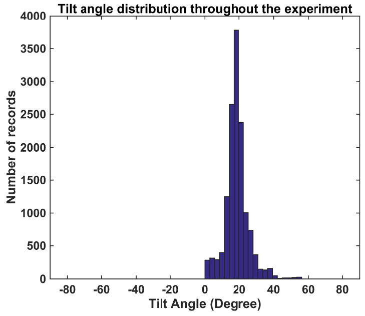
Tilt angle distribution throughout the experiment
Number of records
Tilt Angle (Degree)

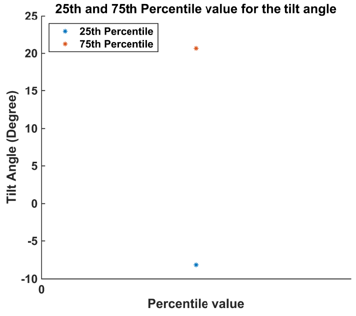
25th and 75th Percentile value for the tilt angle
25th Percentile
75th Percentile
Tilt Angle (Degree)
Percentile value

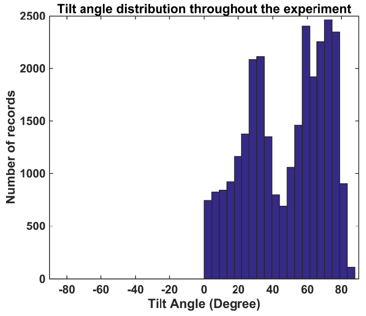
Tilt angle distribution throughout the experiment
Number of records
Tilt Angle (Degree)

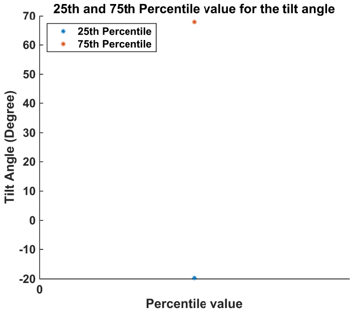
25th and 75th Percentile value for the tilt angle
25th Percentile
75th Percentile
Tilt Angle (Degree)
Percentile value

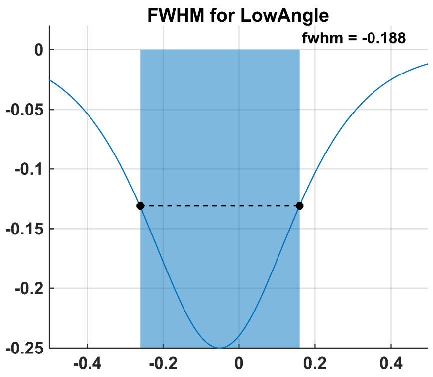
FWHM for LowAngle
fwhm = -0.188

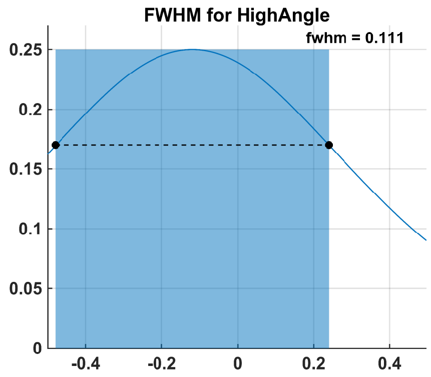
FWHM for HighAngle
fwhm = 0.111

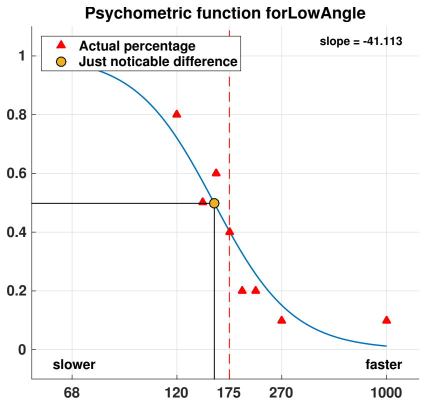
Psychometric function forLowAngle
Actual percentage
Just noticable difference
slope = -41.113
slower
faster

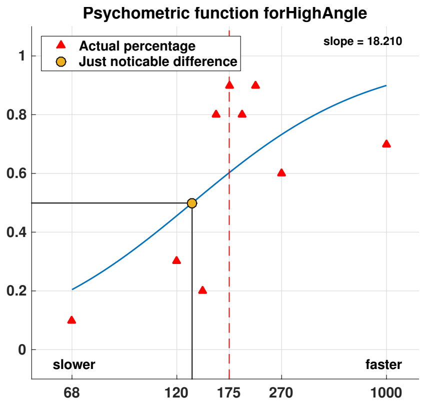
Psychometric function forHighAngle
Actual percentage
Just noticable difference
slope = 18.210
slower
faster

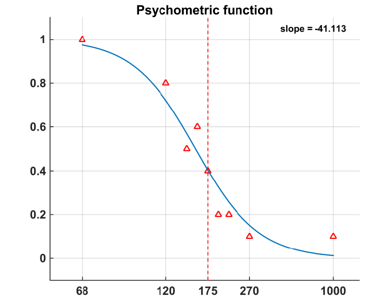
Psychometric function
slope = -41.113

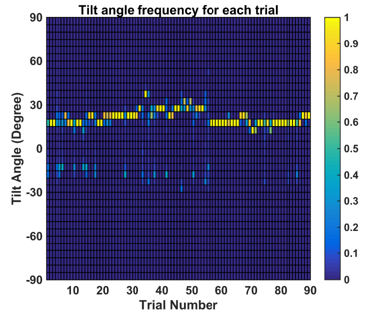
Tilt angle frequency for each trial
Tilt Angle (Degree)
Trial Number

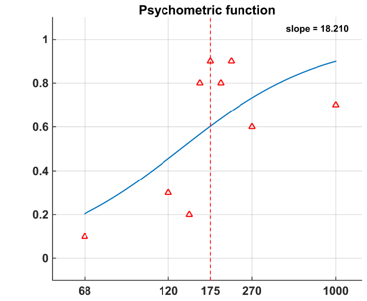
Psychometric function
slope = 18.210

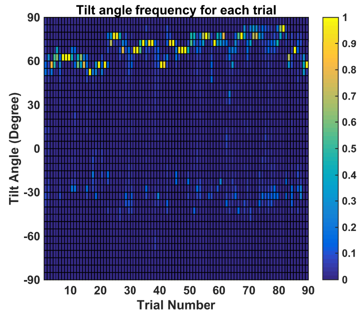
Tilt angle frequency for each trial
Tilt Angle (Degree)
Trial Number

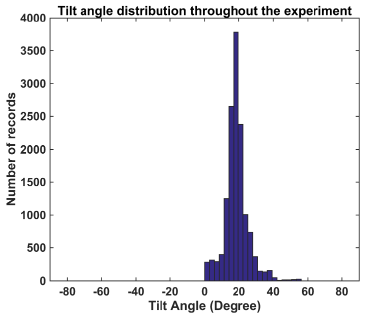
Tilt angle distribution throughout the experiment
Number of records
Tilt Angle (Degree)

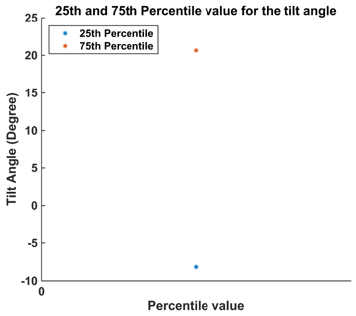
25th and 75th Percentile value for the tilt angle
25th Percentile
75th Percentile
Tilt Angle (Degree)
Percentile value

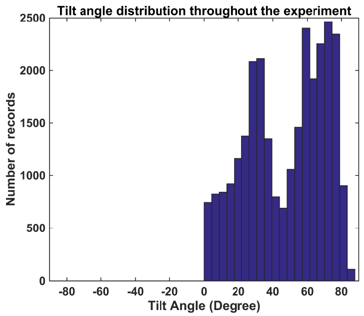
Tilt angle distribution throughout the experiment
Number of records
Tilt Angle (Degree)

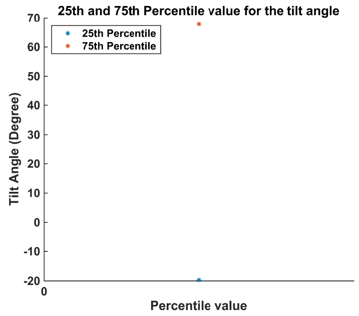
25th and 75th Percentile value for the tilt angle
25th Percentile
75th Percentile
Tilt Angle (Degree)
Percentile value

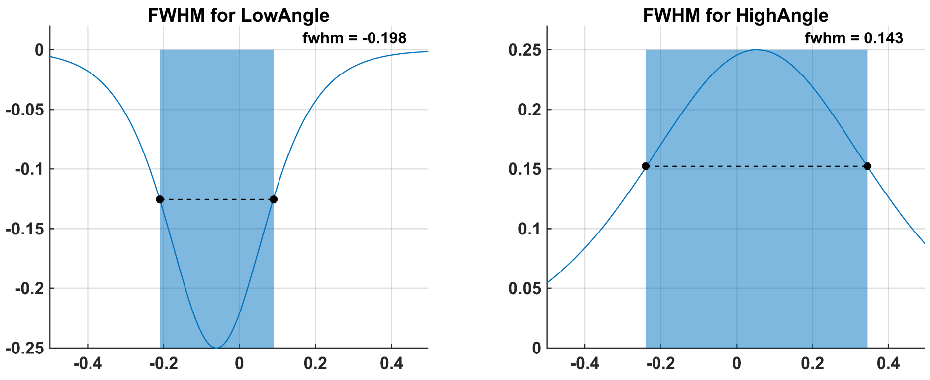
FWHM for LowAngle
fwhm = -0.198
FWHM for HighAngle
fwhm = 0.143

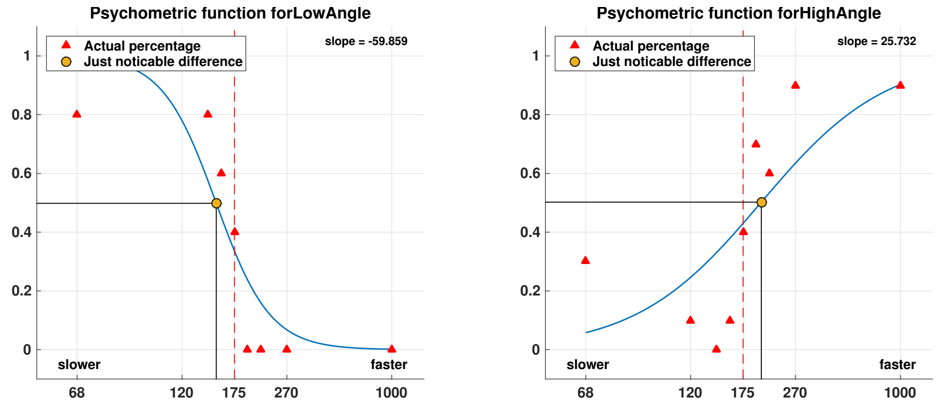
Psychometric function forLowAngle
Actual percentage
Just noticable difference
slope = -59.859
slower
faster
Psychometric function forHighAngle
Actual percentage
Just noticable difference
slope = 25.732
slower
faster

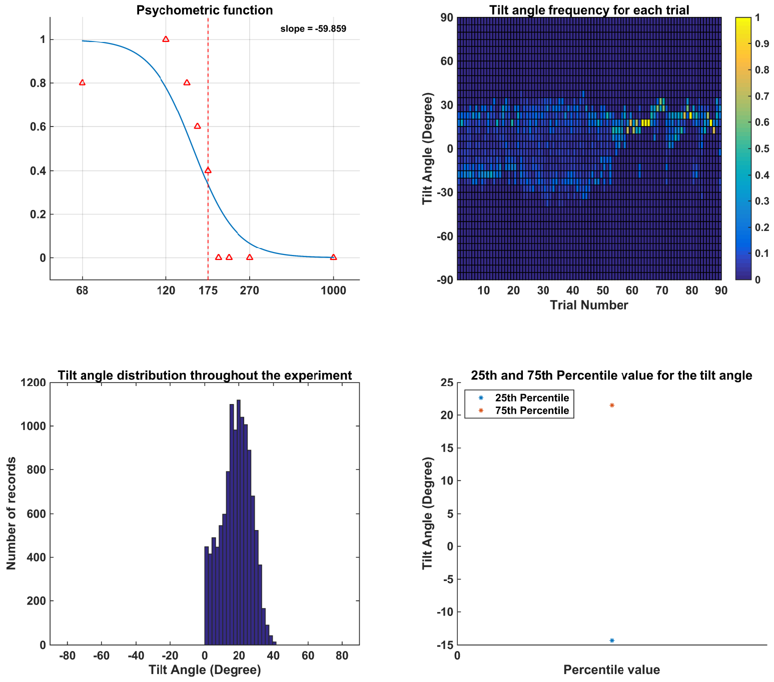
Psychometric function
slope = -59.859
Tilt angle frequency for each trial
Tilt Angle (Degree)
Trial Number
Tilt angle distribution throughout the experiment
Number of records
Tilt Angle (Degree)
25th and 75th Percentile value for the tilt angle
25th Percentile
75th Percentile
Tilt Angle (Degree)
Percentile value

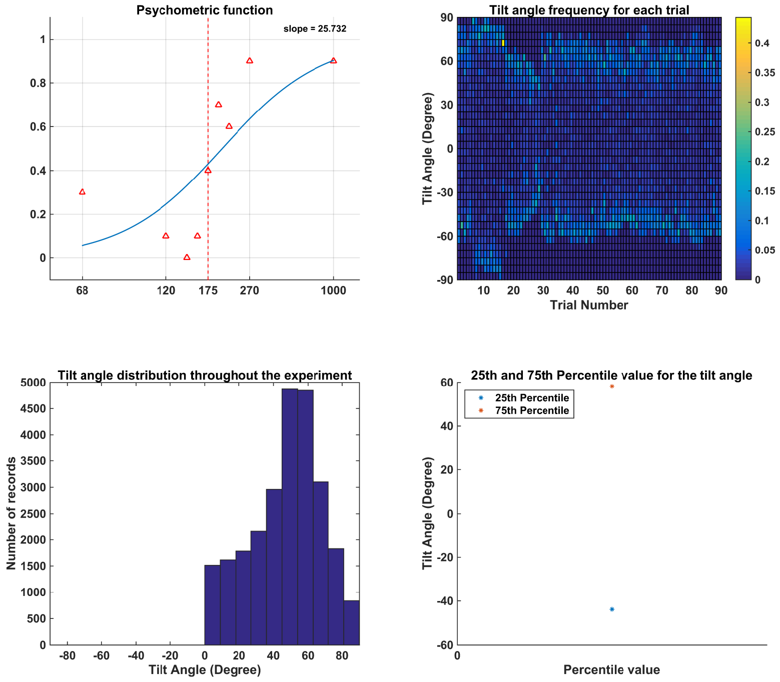
Psychometric function
slope = 25.732
Tilt angle frequency for each trial
Tilt Angle (Degree)
Trial Number
Tilt angle distribution throughout the experiment
Number of records
Tilt Angle (Degree)
25th and 75th Percentile value for the tilt angle
25th Percentile
75th Percentile
Tilt Angle (Degree)
Percentile value

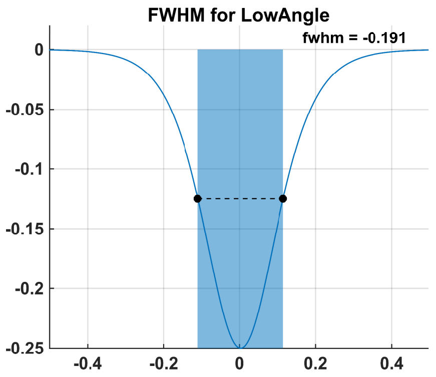
FWHM for LowAngle
fwhm = -0.191

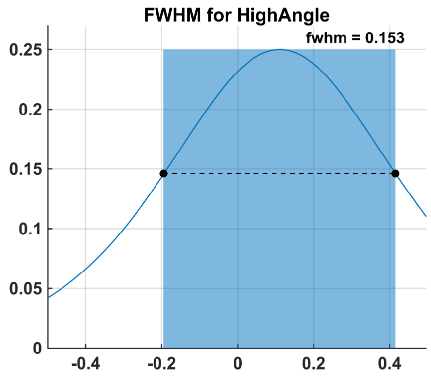
FWHM for HighAngle
fwhm = 0.153

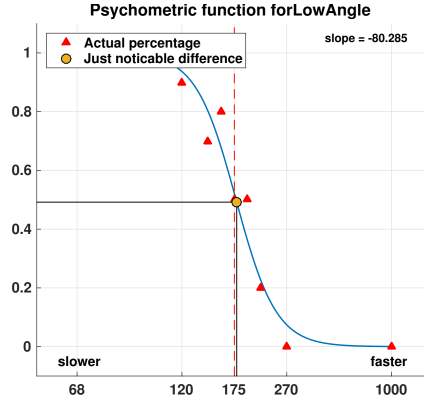
Psychometric function forLowAngle
Actual percentage
Just noticable difference
slope = -80.285
slower
faster

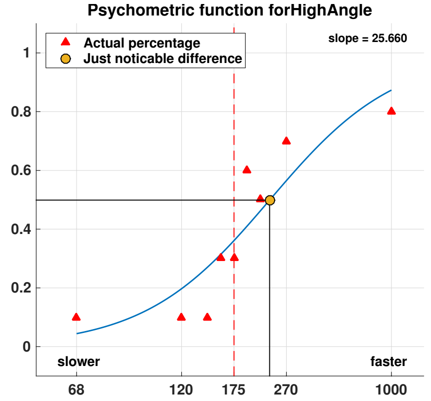
Psychometric function forHighAngle
Actual percentage
Just noticable difference
slope = 25.660
slower
faster

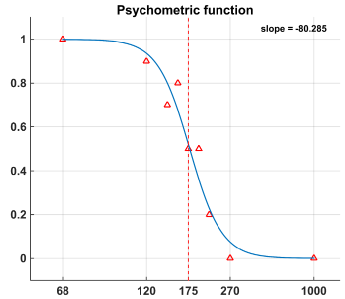
Psychometric function
slope = -80.285

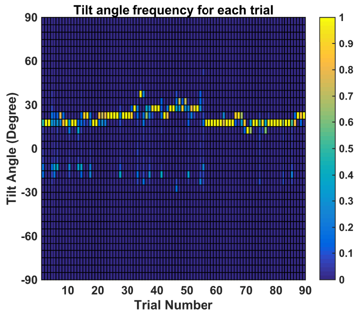
Tilt angle frequency for each trial
Tilt Angle (Degree)
Trial Number

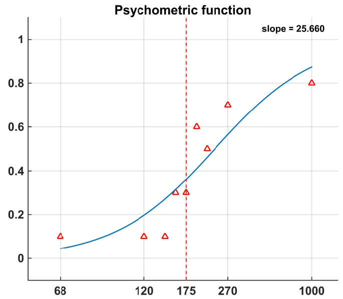
Psychometric function
slope = 25.660

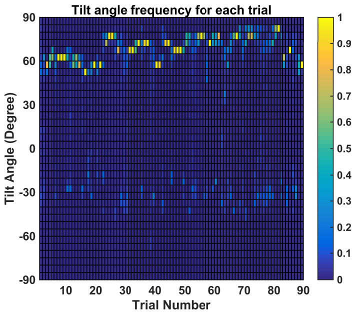
Tilt angle frequency for each trial
Tilt Angle (Degree)
Trial Number

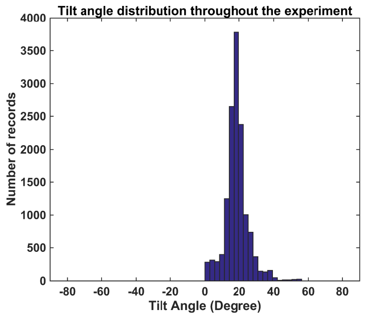
Tilt angle distribution throughout the experiment
Number of records
Tilt Angle (Degree)

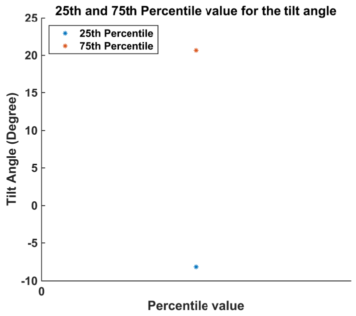
25th and 75th Percentile value for the tilt angle
25th Percentile
75th Percentile
Tilt Angle (Degree)
Percentile value

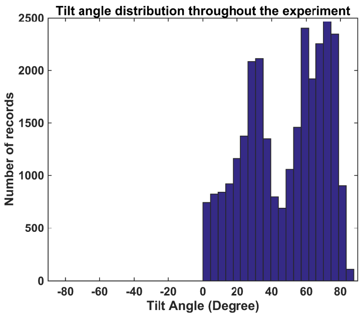
Tilt angle distribution throughout the experiment
Number of records
Tilt Angle (Degree)

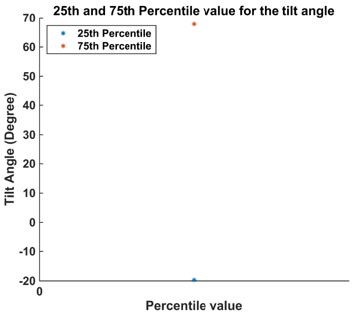
25th and 75th Percentile value for the tilt angle
25th Percentile
75th Percentile
Tilt Angle (Degree)
Percentile value

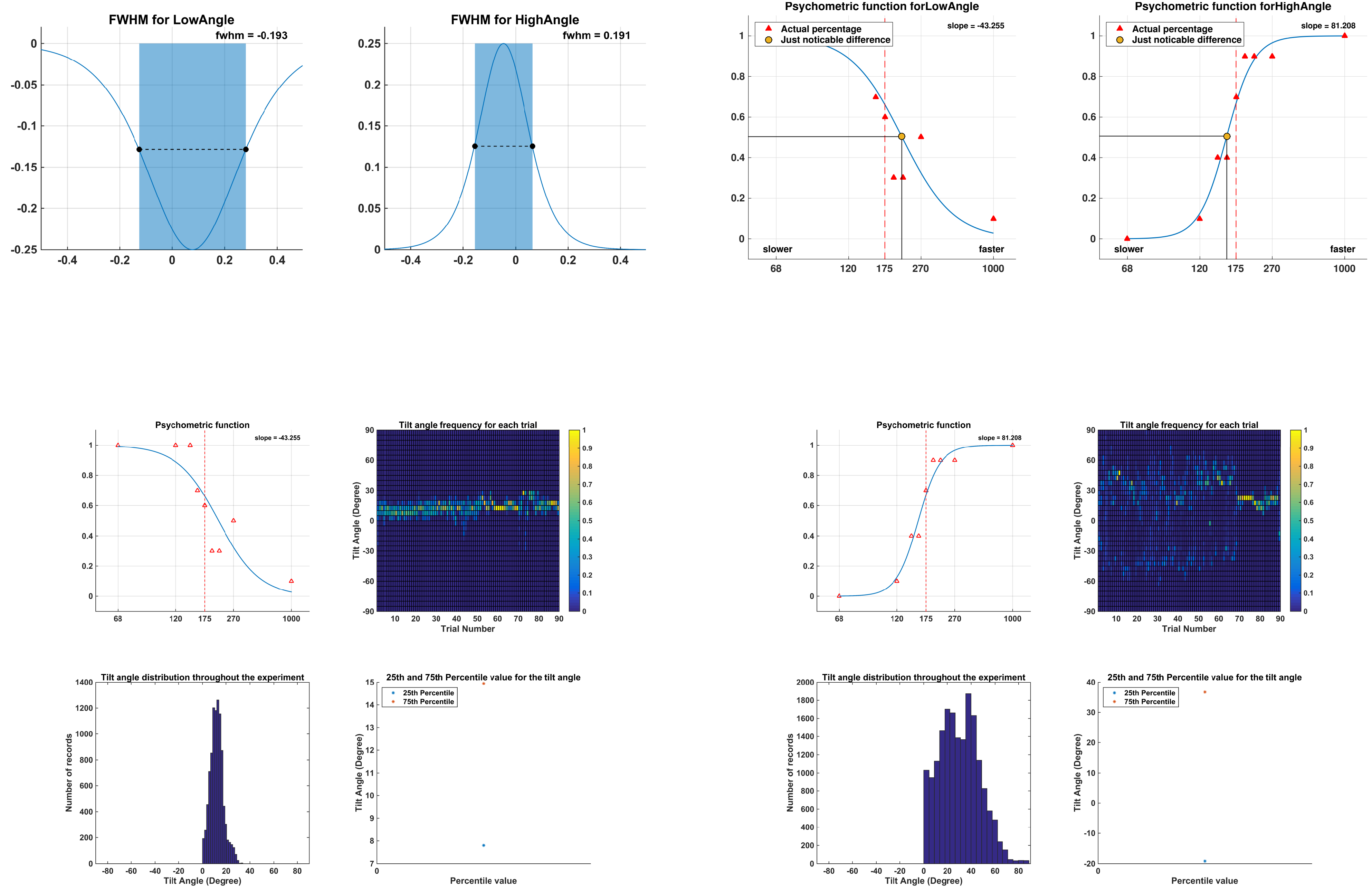

FWHM for LowAngle
fwhm = -0.193
FWHM for HighAngle
fwhm = 0.191
Psychometric function forLowAngle
Actual percentage
Just noticable difference
slope = -43.255
slower
faster
Psychometric function forHighAngle
Actual percentage
Just noticable difference
slope = 81.208
slower
faster
Psychometric function
slope = -43.255
Tilt angle frequency for each trial
Tilt Angle (Degree)
Trial Number
Psychometric function
slope = 81.208
Tilt angle frequency for each trial
Tilt Angle (Degree)
Trial Number
Tilt angle distribution throughout the experiment
Number of records
Tilt Angle (Degree)
25th and 75th Percentile value for the tilt angle
25th Percentile
75th Percentile
Tilt Angle (Degree)
Percentile value
Tilt angle distribution throughout the experiment
Number of records
Tilt Angle (Degree)
25th and 75th Percentile value for the tilt angle
25th Percentile
75th Percentile
Tilt Angle (Degree)
Percentile value

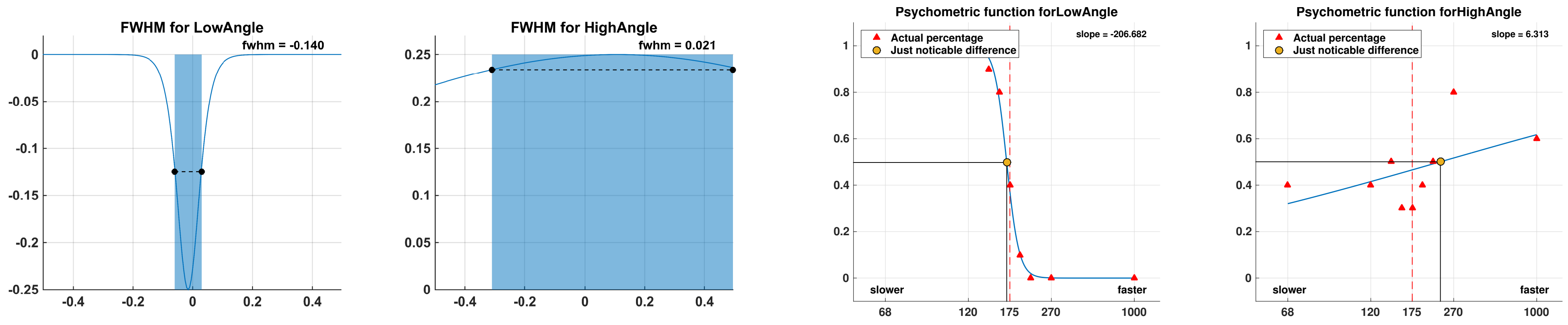
FWHM for LowAngle
fwhm = -0.140
FWHM for HighAngle
fwhm = 0.021
Psychometric function forLowAngle
Actual percentage
Just noticable difference
slope = -206.682
slower
faster
Psychometric function forHighAngle
slope = 6.313

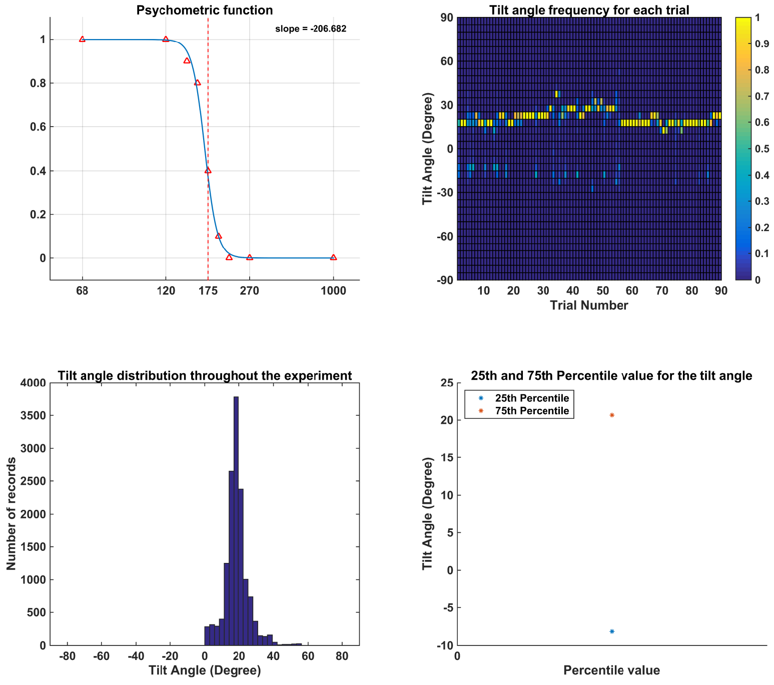
Psychometric function
slope = -206.682
Tilt angle frequency for each trial
Tilt Angle (Degree)
Trial Number
Tilt angle distribution throughout the experiment
Number of records
Tilt Angle (Degree)
25th and 75th Percentile value for the tilt angle
25th Percentile
75th Percentile
Percentile value

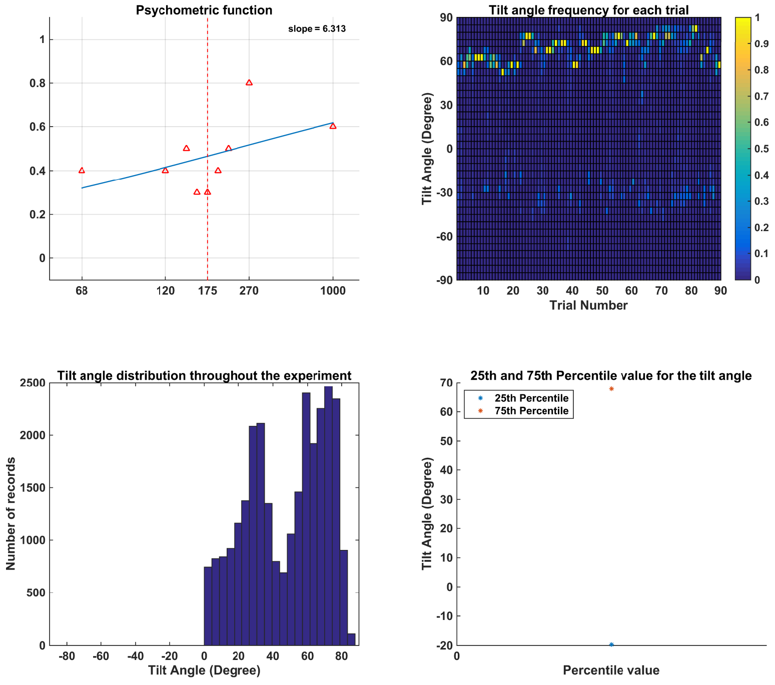
Psychometric function
slope = 6.313
Tilt angle frequency for each trial
Tilt Angle (Degree)
Trial Number
Tilt angle distribution throughout the experiment
Number of records
Tilt Angle (Degree)
25th and 75th Percentile value for the tilt angle
25th Percentile
75th Percentile
Percentile value

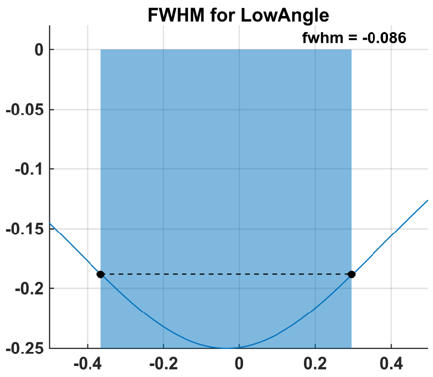
FWHM for LowAngle
fwhm = -0.086

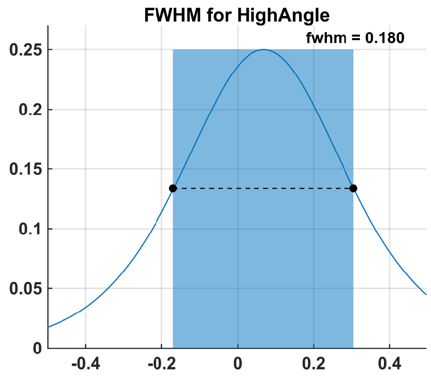
FWHM for HighAngle
fwhm = 0.180

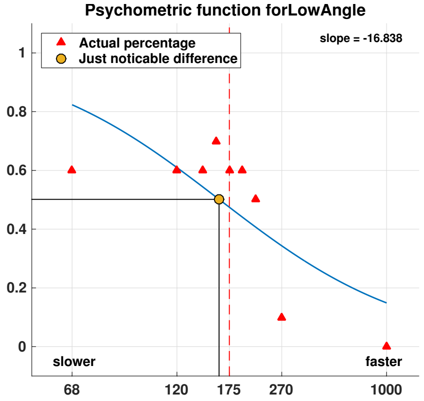
Psychometric function forLowAngle
Actual percentage
Just noticable difference
slope = -16.838
slower
faster

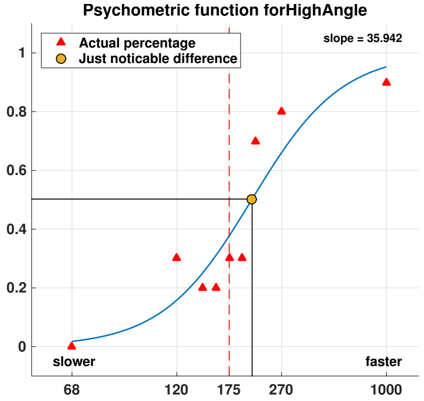
Psychometric function forHighAngle
Actual percentage
Just noticable difference
slope = 35.942
slower
faster

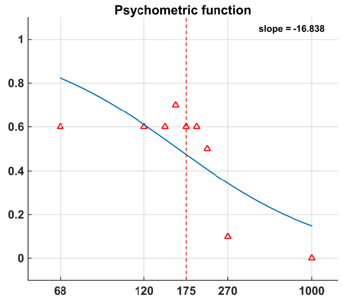
Psychometric function
slope = -16.838

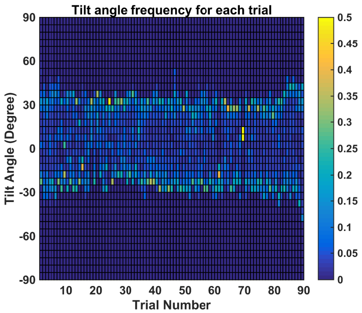
Tilt angle frequency for each trial
Tilt Angle (Degree)
Trial Number

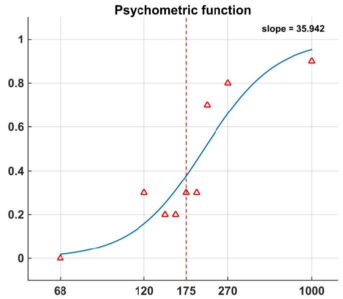
Psychometric function
slope = 35.942

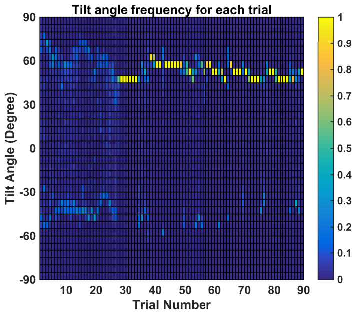
Tilt angle frequency for each trial
Tilt Angle (Degree)
Trial Number

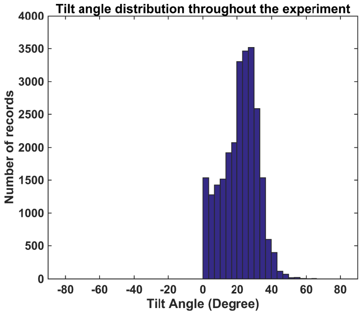
Tilt angle distribution throughout the experiment
Number of records
Tilt Angle (Degree)

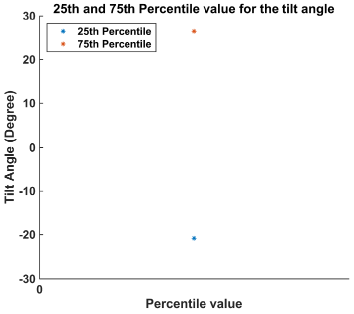
25th and 75th Percentile value for the tilt angle
25th Percentile
75th Percentile
Tilt Angle (Degree)
Percentile value

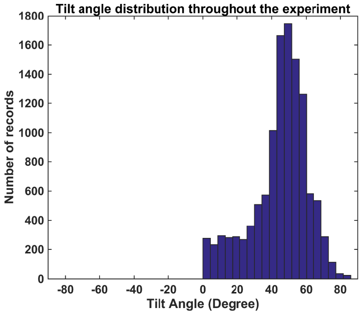
Tilt angle distribution throughout the experiment
Number of records
Tilt Angle (Degree)

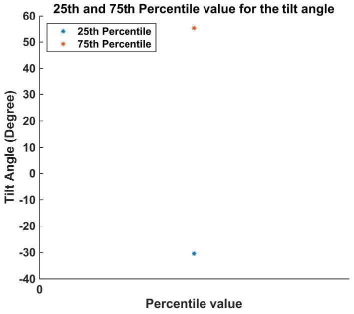
25th and 75th Percentile value for the tilt angle
25th Percentile
75th Percentile
Tilt Angle (Degree)
Percentile value

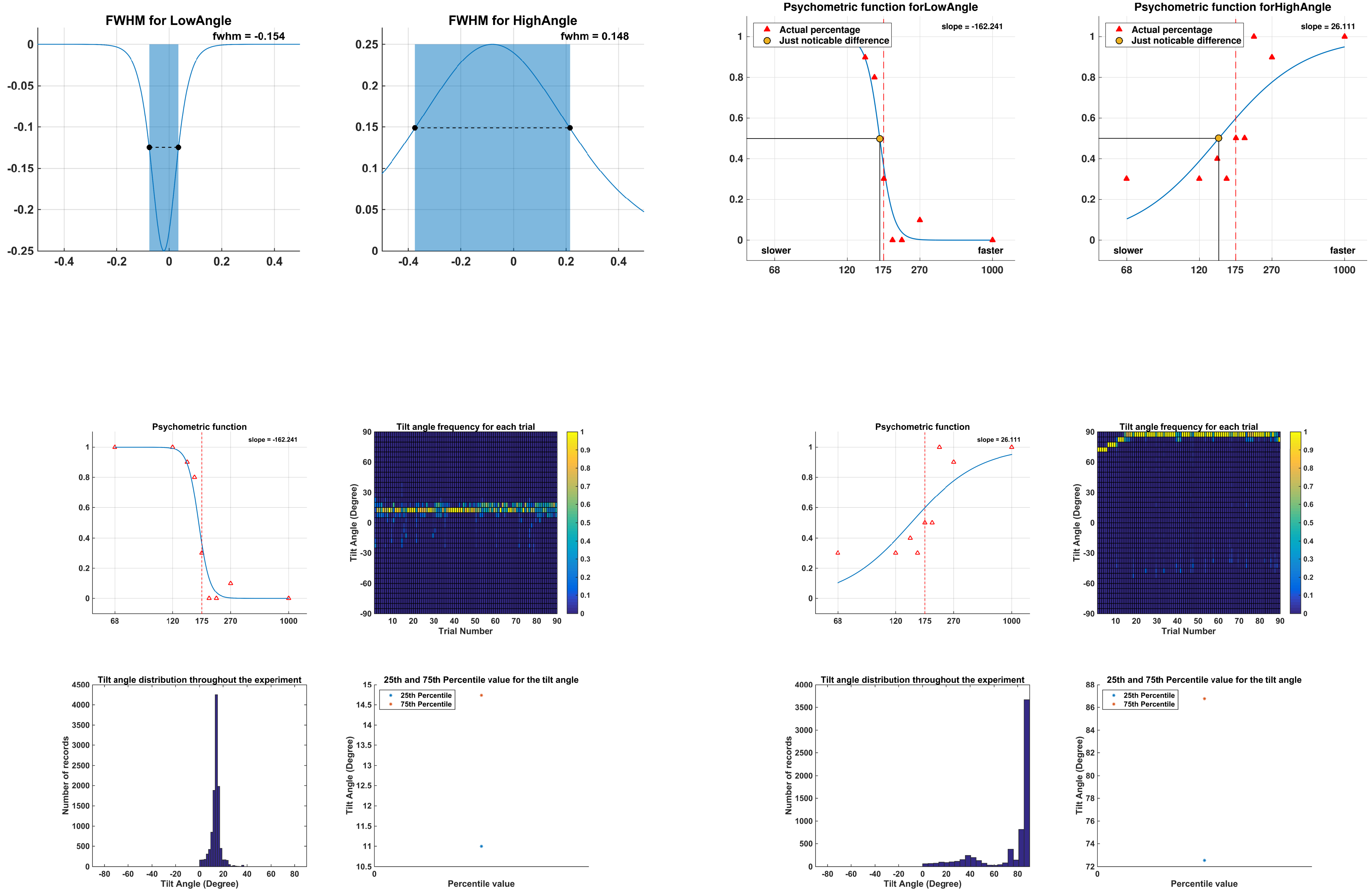
FWHM for LowAngle
fwhm = -0.154
FWHM for HighAngle
fwhm = 0.148
Psychometric function forLowAngle
slope = -162.241
Actual percentage
Just noticable difference
slower
faster
Psychometric function forHighAngle
slope = 26.111
Psychometric function
slope = -162.241
Tilt angle frequency for each trial
Tilt Angle (Degree)
Trial Number
Psychometric function
slope = 26.111
Tilt angle frequency for each trial
Tilt angle distribution throughout the experiment
Number of records
Tilt Angle (Degree)
25th and 75th Percentile value for the tilt angle
25th Percentile
75th Percentile
Percentile value
Tilt angle distribution throughout the experiment
25th and 75th Percentile value for the tilt angle

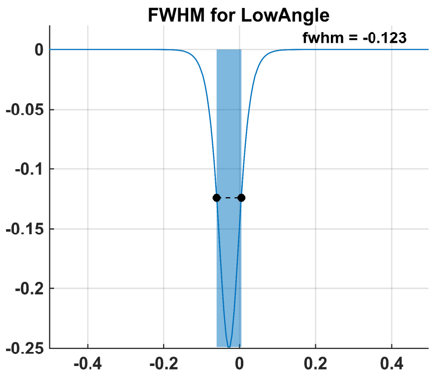
FWHM for LowAngle
fwhm = -0.123

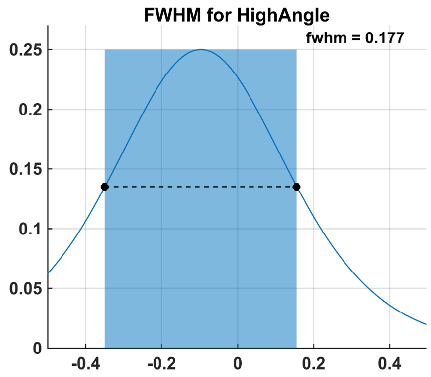
FWHM for HighAngle
fwhm = 0.177

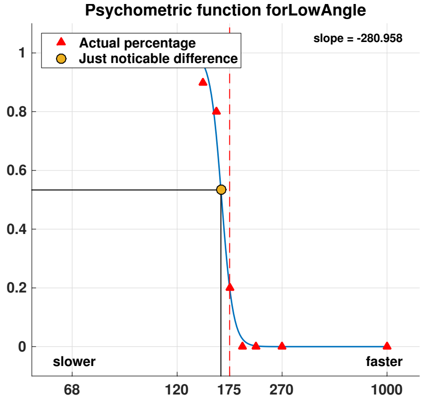
Psychometric function forLowAngle
Actual percentage
Just noticable difference
slope = -280.958
slower
faster

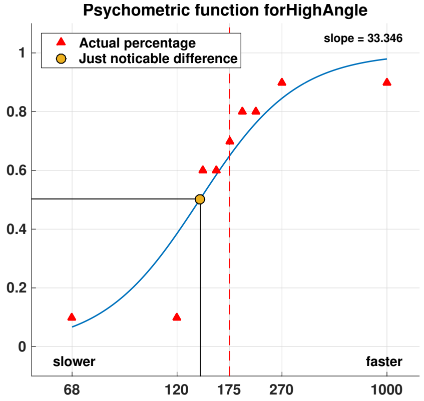
Psychometric function forHighAngle
Actual percentage
Just noticable difference
slope = 33.346
slower
faster

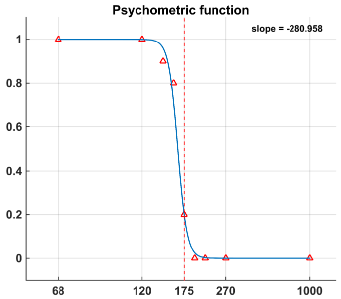
Psychometric function
slope = -280.958

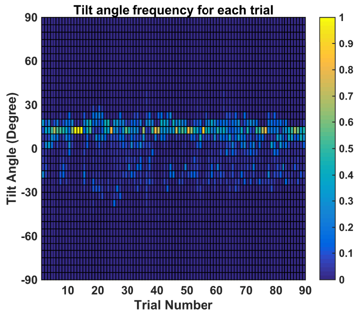
Tilt angle frequency for each trial
Tilt Angle (Degree)
Trial Number

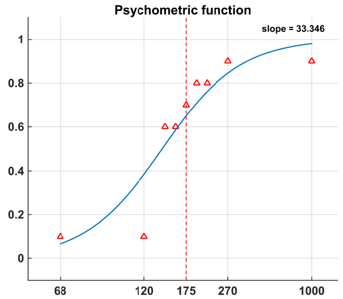
Psychometric function
slope = 33.346

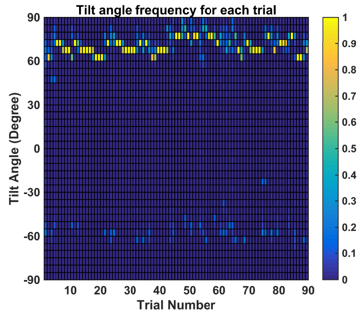
Tilt angle frequency for each trial
Tilt Angle (Degree)
Trial Number

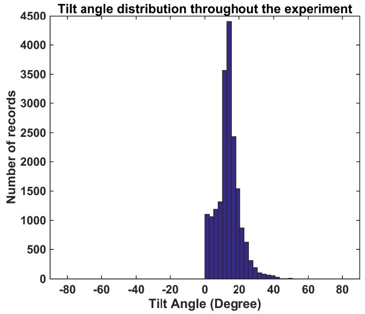
Tilt angle distribution throughout the experiment
Number of records
Tilt Angle (Degree)

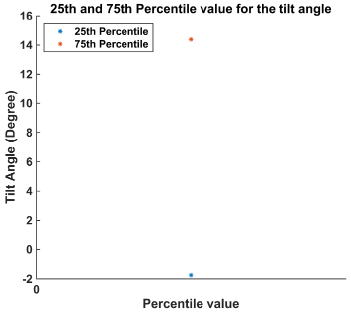
25th and 75th Percentile value for the tilt angle
25th Percentile
75th Percentile
Tilt Angle (Degree)
Percentile value

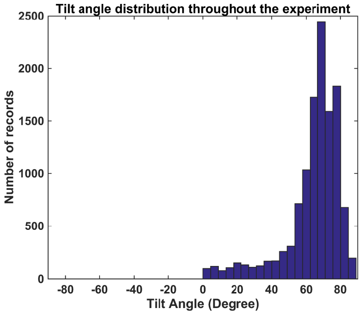
Tilt angle distribution throughout the experiment
Number of records
Tilt Angle (Degree)

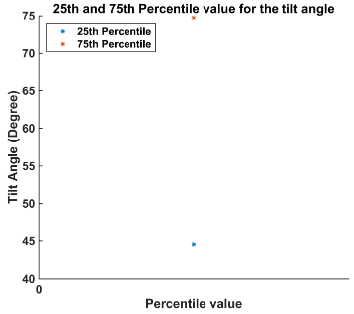
25th and 75th Percentile value for the tilt angle
25th Percentile
75th Percentile
Tilt Angle (Degree)
Percentile value

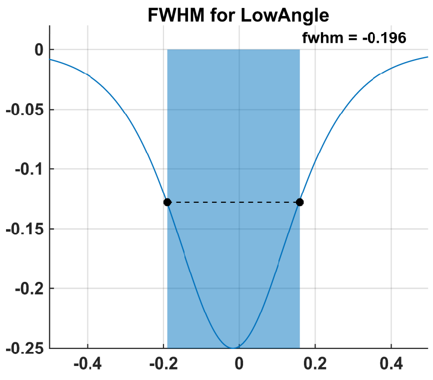
FWHM for LowAngle
fwhm = -0.196

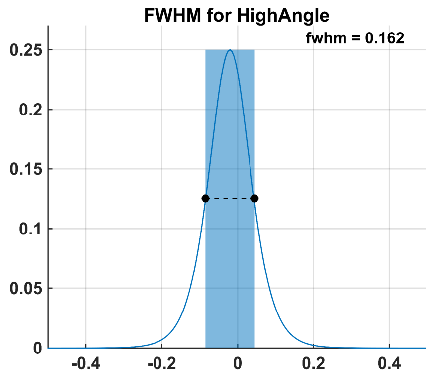
FWHM for HighAngle
fwhm = 0.162

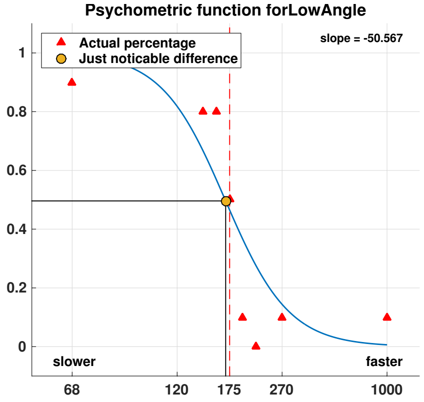
Psychometric function forLowAngle
Actual percentage
Just noticable difference
slope = -50.567
slower
faster

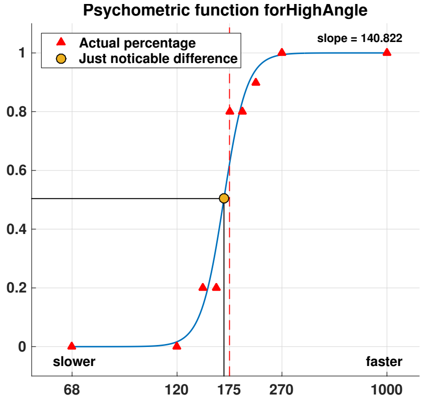
Psychometric function forHighAngle
Actual percentage
Just noticable difference
slope = 140.822
slower
faster

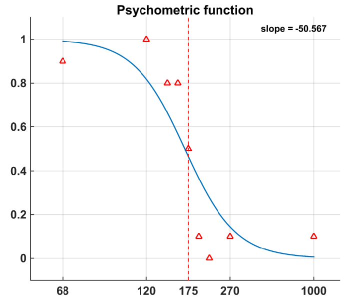
Psychometric function
slope = -50.567

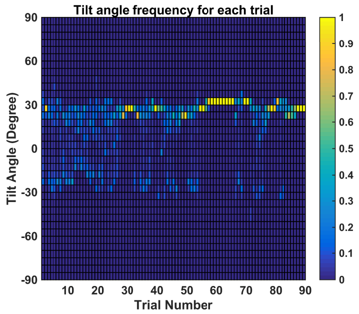
Tilt angle frequency for each trial
Tilt Angle (Degree)
Trial Number

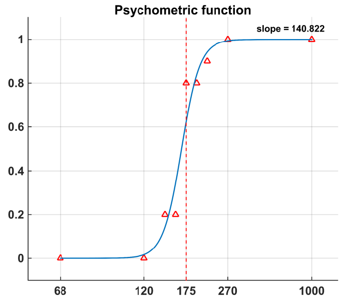
Psychometric function
slope = 140.822

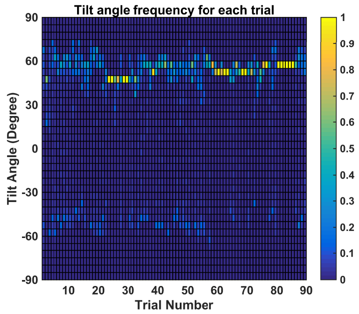
Tilt angle frequency for each trial
Tilt Angle (Degree)
Trial Number

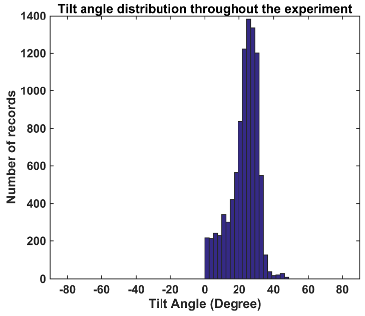
Tilt angle distribution throughout the experiment
Number of records
Tilt Angle (Degree)

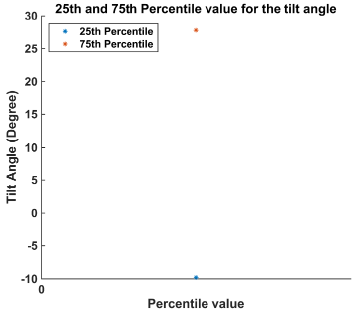
25th and 75th Percentile value for the tilt angle
25th Percentile
75th Percentile
Tilt Angle (Degree)
Percentile value

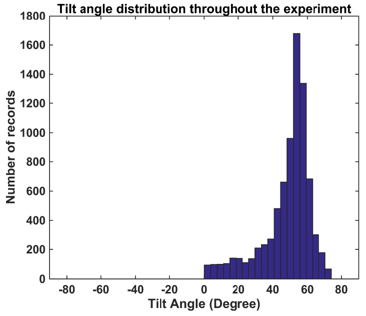
Tilt angle distribution throughout the experiment
Number of records
Tilt Angle (Degree)

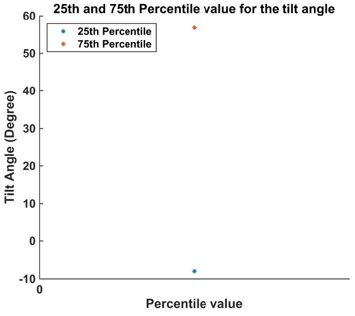
25th and 75th Percentile value for the tilt angle
25th Percentile
75th Percentile
Tilt Angle (Degree)
Percentile value

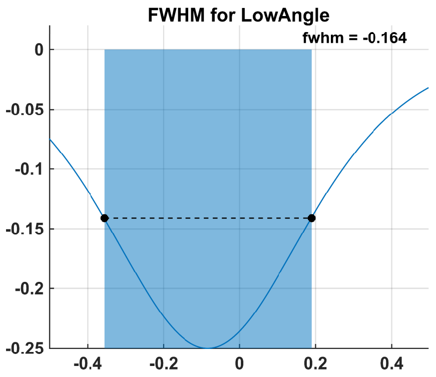
FWHM for LowAngle
fwhm = -0.164

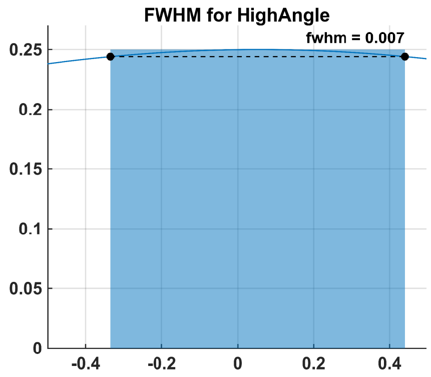
FWHM for HighAngle
fwhm = 0.007

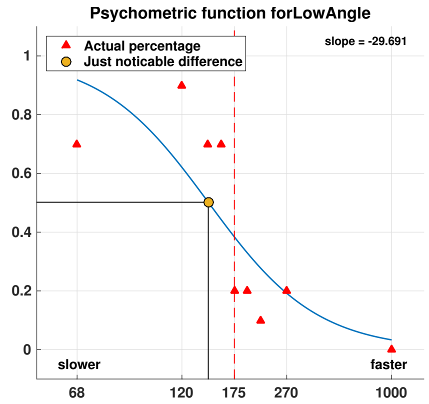
Psychometric function forLowAngle
Actual percentage
Just noticable difference
slope = -29.691
slower
faster

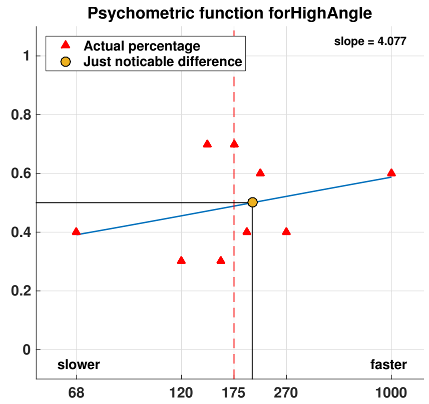
Psychometric function forHighAngle
Actual percentage
Just noticable difference
slope = 4.077
slower
faster

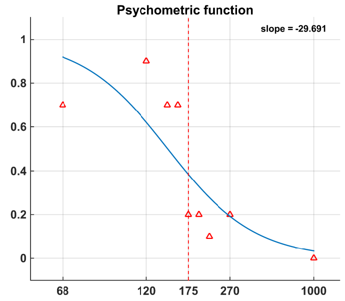
Psychometric function
slope = -29.691

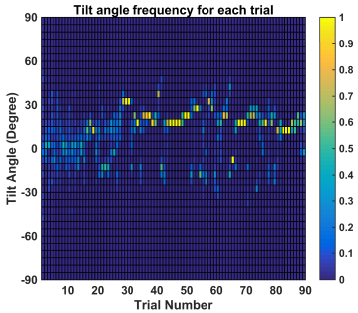
Tilt angle frequency for each trial
Tilt Angle (Degree)
Trial Number

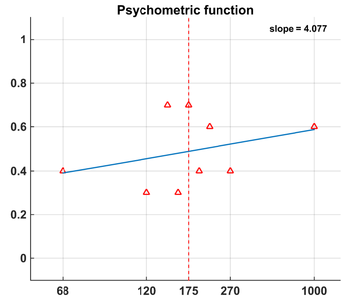
Psychometric function
slope = 4.077

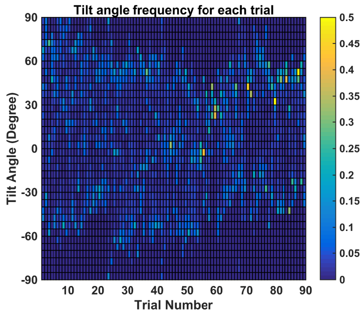
Tilt angle frequency for each trial
Tilt Angle (Degree)
Trial Number

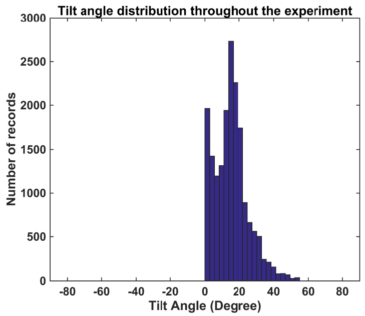
Tilt angle distribution throughout the experiment
Number of records
Tilt Angle (Degree)

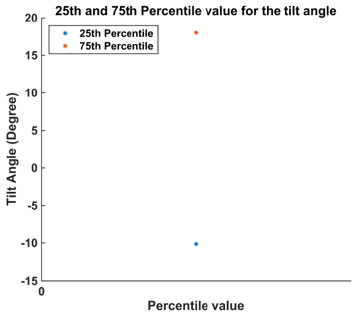
25th and 75th Percentile value for the tilt angle
25th Percentile
75th Percentile
Tilt Angle (Degree)
Percentile value

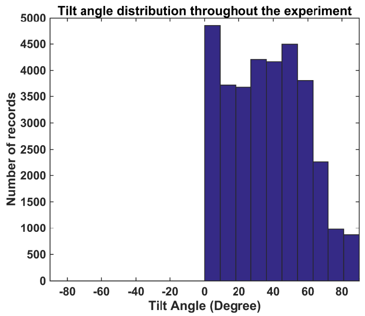
Tilt angle distribution throughout the experiment
Number of records
Tilt Angle (Degree)

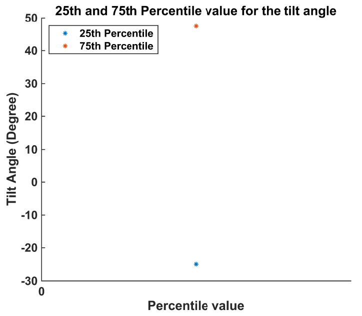
25th and 75th Percentile value for the tilt angle
25th Percentile
75th Percentile
Tilt Angle (Degree)
Percentile value

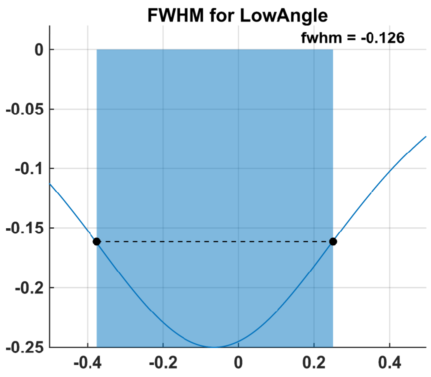
FWHM for LowAngle
fwhm = -0.126

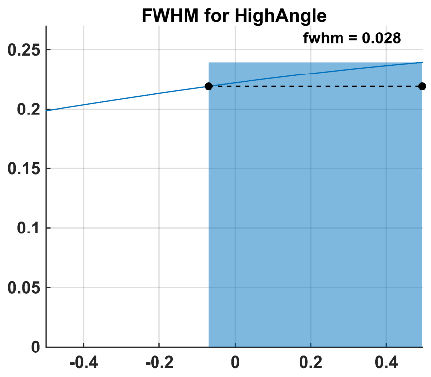
FWHM for HighAngle
fwhm = 0.028

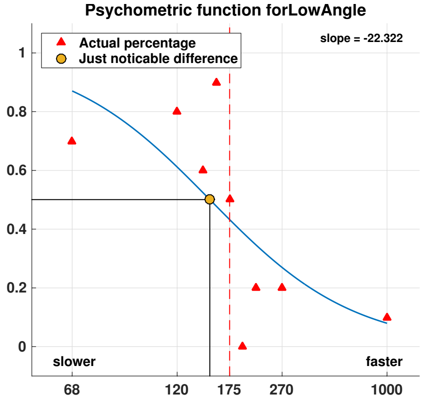
Psychometric function forLowAngle
Actual percentage
Just noticable difference
slope = -22.322
slower
faster

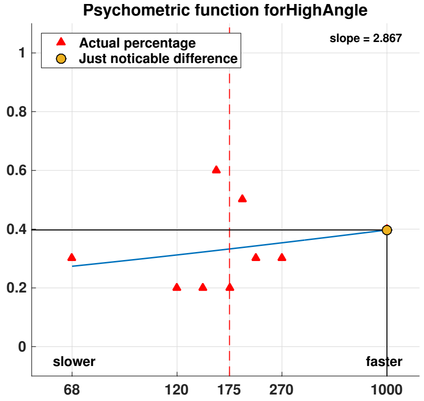
Psychometric function forHighAngle
Actual percentage
Just noticable difference
slope = 2.867
slower
faster

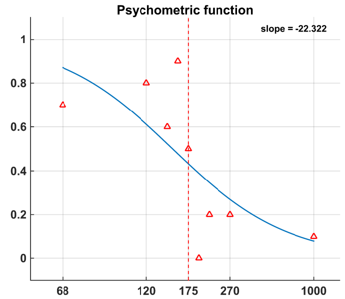
Psychometric function
slope = -22.322

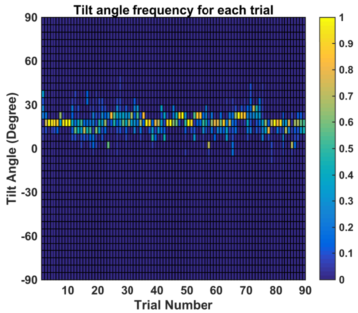
Tilt angle frequency for each trial
Tilt Angle (Degree)
Trial Number

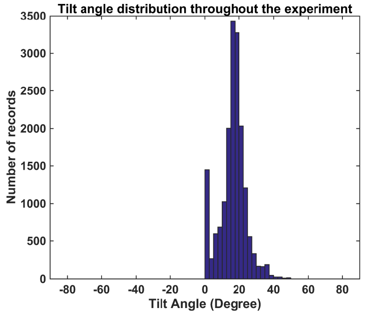
Tilt angle distribution throughout the experiment
Number of records
Tilt Angle (Degree)

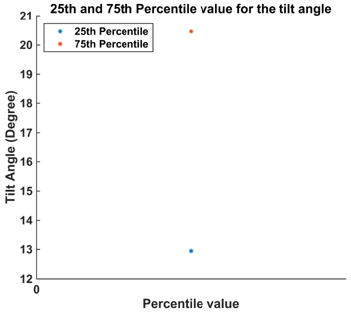
25th and 75th Percentile value for the tilt angle
25th Percentile
75th Percentile
Tilt Angle (Degree)
Percentile value

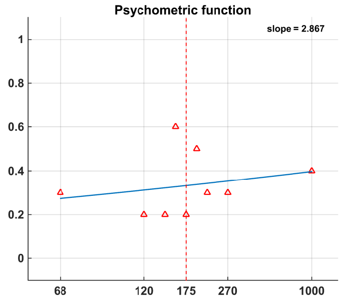
Psychometric function
slope = 2.867

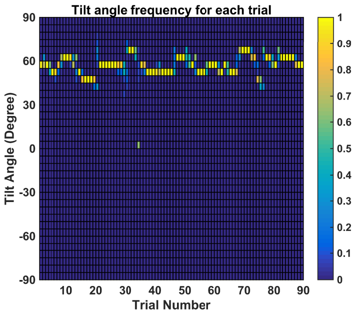
Tilt angle frequency for each trial
Tilt Angle (Degree)
Trial Number

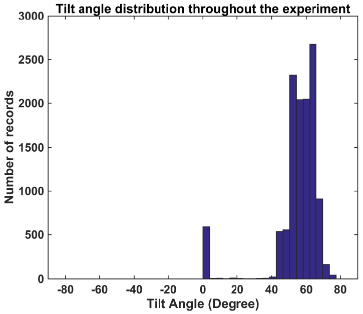
Tilt angle distribution throughout the experiment
Number of records
Tilt Angle (Degree)

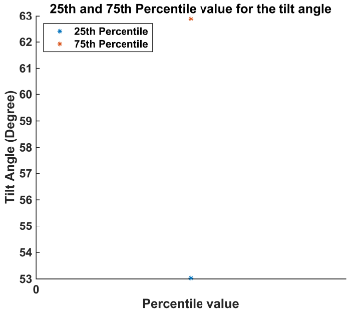
25th and 75th Percentile value for the tilt angle
25th Percentile
75th Percentile
Tilt Angle (Degree)
Percentile value
